# Supplementary material for: Improving physical function of patients following intensive care unit admission (EMPRESS): protocol of a randomised controlled feasibility trial
Source: BMJ Open. 2022 Apr 15;12(4):e055285. doi: 10.1136/bmjopen-2021-055285 (PMC9014051; doi:10.1136/bmjopen-2021-055285)
Supplement: Supplementary data [file bmjopen-2021-055285supp001.pdf]

Site specific header to be inserted here

EMPRESS.

A feasibility study of early mobilisation in Critical Care.

Patient Information Sheet

Version 2: 29th January 2019

Introduction

Study Title: EMPRESS: A study of very early mobilisation in Critical Care.

Invitation:

Your consultee has agreed on your behalf, to your participation on a research study. We would like to invite you to confirm whether you wish to continue or withdraw your participation from this research study.

This hospital is taking part in a national research study to investigate whether starting rehabilitation in the Intensive Care Unit, as soon as possible, will improve patient's long-term physical ability and quality of life.

When patients are sedated in Intensive Care, muscle wasting and weakness can occur very quickly and this can take a long time to recover from. Because we feel that it may be important to deliver rehabilitation physiotherapy as early as possible, it was agreed by your doctor and / or your relative/ friend that you could be involved in this study. This research has been approved by Hampshire Research Ethics Committee (IRAS number: 250165).

This patient information sheet provides information about the study to help you decide if you would like to continue to participate in it. It is important that you understand why the research is being done and what it involves.

Knowing what is involved will help you decide if you want to continue to take part in the research, so this Information Sheet explains the tests and treatments involved.

- Part 1 tells you about why we are doing this study and what will happen to you if you continue to take part.
- Part 2 gives you more detailed information about how we will run the study.

If you have no objection to continue taking part, we will ask you to read and sign a form that records your permission, called the consent declaration. We'll then give you a copy to keep. We will keep you fully informed during the study so you can let us know if you have any concerns or if you would prefer to be withdrawn. Taking part in this research is entirely voluntary. If you decide not to continue, you will still be offered the best possible standard of care.

Please ask us if there is anything that is not clear or if you would like more information. Take time to decide whether or not you wish to take part.

Cusack R, *et al. BMJ Open* 2022; 12:e055285. doi: 10.1136/bmjopen-2021-055285

PART ONE: Why are we doing this study and what will happen to you?

What is the purpose of this study?

We know that when patients are very unwell and need sedation in the Intensive Care Unit, they can lose muscle strength and size very quickly. It is normal to offer rehabilitation, but this often starts after the patient has woken up. By this time the muscles have already been affected. Previous studies have shown that this can take many months to recover from and may affect a patient's quality of life after leaving hospital.

In Southampton Hospital, researchers and physiotherapists started performing rehabilitation exercises much earlier than usual, even while the patient was sedated. They showed that this method reduced the patient's time on the ventilator and reduced the amount of time that they needed to be in Intensive Care.

We are now trying to discover whether this method will work in a number of different hospitals in the UK. We will also do some tests to see whether the patients who have this type of rehabilitation are stronger and able to engage in physical activity more easily, when they leave hospital and 3 months later.

Why have you been chosen?

You were enrolled in this study because during your admission to the Intensive Care you needed a ventilator (a machine to help you breathe) and sedation was needed to help keep you calm and comfortable. The treating doctor and physiotherapist thought that either very early rehabilitation or standard rehabilitation would be equally suitable. We may have given you very early physiotherapy already in the intensive care unit, because we are testing such early rehabilitation, but we would like to ask for your permission to continue.

Do I have to take part?

No. It is up to you to decide whether or not you would like to continue to take part. If you do, you will be given this information sheet to keep and be asked to sign the consent form. You are still free to withdraw at any time without giving a reason. The decision to withdraw at any time, or a decision not to take part, will not affect the standard of care you receive.

What will happen to me if I take part?

Participation in the study began in the Intensive Care Unit. The final tests will take place 3 months after you leave hospital.

In the Intensive Care Unit: Your treating doctor has assessed you to be eligible to take part in this study: EMPRESS. You were randomly allocated (like the flip of a coin) to receive either of the following:

- **Standard physiotherapy:** All patients on the trial will receive their normal physiotherapy. This will normally include activities to assist in keeping your airway

clear and activities to maintain limb flexibility. These will not be affected by being on the trial.

OR

- **Standard physiotherapy, as above, plus an extra 2 sessions of 30 minutes of rehabilitation from Monday to Friday.** For patients receiving extra, early rehabilitation, in addition to your normal physiotherapy, you have been using a cycle machine that is designed to work, in the bed, even with sedated patients. As you wake up you have started to pedal for yourself, do some more bed-based exercises and finally get out of bed and start moving. All of these sessions have been and will continue to be run by a well-trained physiotherapist and the bedside nurses. We have already tested this method in University Hospital Southampton and it has reduced the length of time on the ventilator and ICU stay. During these sessions, you have been and will continue to be very carefully monitored for your own safety and the safety of lines, tubes and catheters.

  These exercises will continue for a maximum of 28 days or less if you leave the Intensive care unit before then.

BOTH GROUPS

- **Additional assessments:** So that we can test whether our new method works, patients on the trial will undertake some extra assessments. These include a simple test of grip strength by using a hand held pressure monitor; a test of arm and leg strength, ability to stand and step and mobility and walking tests. There will also be quality of life and health questionnaires.

There was a 50/50 chance of being allocated to either group. Neither you nor your doctor can decide which. No samples of blood are required for this research study.

In the hospital ward: When you have been discharged to a normal hospital ward, you won't receive any extra physiotherapy. Just before you go home, you will be tested again for muscle strength and mobility, including how far you can walk in 6 minutes. These tests will be supervised by a trained and experienced physiotherapist

Following discharge from hospital: Regardless of which group you were allocated to, after going home, you should follow the advice given to you by your doctors and physiotherapists. We have designed our study so that this will not affect our results.

You will be contacted by one of the critical care research team 3 months after you have been discharged home. We will arrange to see you for approximately one hour. During this visit we will test your walking speed, strength and agility. We will also ask for some questionnaires to be completed, which will assess how you feel about your quality of life and recovery.

The researchers would also like to have access to your medical record to obtain information relevant to the study. This information would be anonymised and kept confidential.

If you have any questions regarding the trial procedures, please don't hesitate to ask the intensive care or research doctors, physiotherapists and nurses.

What do I have to do?

It is important to tell the doctor and the research staff about any treatments or medications you may have been taking, including over-the-counter medications, vitamins or herbal remedies, acupuncture or other alternative treatments. We would also like to know about any medical conditions which may affect the exercise.

Please let us know if you are involved in any other studies at this time.

What are the alternatives to participation?

Participation in this research is not the only option. You may decide to receive only standard or usual care. This is absolutely fine. Please feel free to discuss these options with your doctor before deciding whether or not to continue to take part in this research project.

What are the possible disadvantages of taking part?

Early mobility within ICU is safe. Potential risks may include, but not be limited to blood pressure or heart rate problems, breathing problems, problems with the tubes, lines and catheters.

In a review of physiotherapy within Intensive Care Units, involving over 1100 patients and 5267 episodes of physiotherapy in similar patients, there were 34 potential safety events (equivalent to 6 events in 1000 episodes of physiotherapy), Most of these were potentially related to changes in heart rate or blood pressure which settle quickly in stopping the physiotherapy.

In Southampton, over a four year period, we have treated over 500 patients in this way and had 2 non-serious adverse events.

The doctors, physiotherapists and nurses who will be caring for you while in the ICU, are trained to recognise the effects on the body associated with physical rehabilitation and will treat you accordingly. You will be continue to be monitored and assessed. Your safety is always our number one priority.

There may be side effects that the researchers do not expect or do not know about and that may be serious. Please tell the doctor immediately about any new or unusual symptoms that you get.

What are the possible benefits of taking part?

We cannot guarantee or promise that you will receive any benefits from this research. This study aims to further medical knowledge and may improve future treatment of patients who need to be on a ventilator, however it may not directly benefit you.

For how long will I be in the research study?

The final research assessment will take place 3 months after discharge from hospital. Once that is done, your participation in the study will end.

Cusack R, *et al. BMJ Open* 2022; 12:e055285. doi: 10.1136/bmjopen-2021-055285

What happens if there is a problem?

We will keep you fully informed of any problems which may be related to the study.

Will taking part in the study be kept confidential?

Yes. All of the information about participation and the data collected will be kept confidential.

Information held by the NHS and records maintained by the NHS Information Centre and the NHS Central Register may be used to help contact you and provide information about your health status. This information may be obtained and stored by the study research team to enable long term follow-up.

University Hospital Southampton is the sponsor for this study based in the United Kingdom. We will be using information from you and/or your medical records in order to undertake this study and will act as the data controller for this study. This means that we are responsible for looking after your information and using it properly. University Hospital Southampton will keep information about you for 10 years after the study has finished.

Your rights to access, change or move your information are limited, as we need to manage your information in specific ways in order for the research to be reliable and accurate. If you withdraw from the study, we will keep the information about you that we have already obtained. To safeguard your rights, we will use the minimum personally-identifiable information possible.

You can find out more about how we use your information at https://www.hra.nhs.uk/information-about-patients/

[Local NHS site name] will collect information from you and/or your medical records for this research study in accordance with our instructions.

(Local NHS site name) will keep your name, NHS number and contact details confidential and will not pass this information to University Hospital Southampton. [Local NHS site name] will use this information as needed, to contact you about the research study, and make sure that relevant information about the study is recorded for your care, and to oversee the quality of the study. Certain individuals from University Hospital Southampton and regulatory organisations may look at your medical and research records to check the accuracy of the research study. University Hospital Southampton will only receive information without any identifying information. The people who analyse the information will not be able to identify you and will not be able to find out your name, NHS number or contact details.

[Local NHS site name] will keep identifiable information about you, including the consent form from this study for 10 years after the study has finished.

Cusack R, *et al. BMJ Open* 2022; 12:e055285. doi: 10.1136/bmjopen-2021-055285

Contact Details:

Local PI

Consultant Critical Care,

Hospital address

Dr XXXX: 02381 XXXXXX

Research Nurse: 02381 XXXXXX

ICU: 02381 XXXXXX

PART 2: How we will run this study.

What if relevant new information becomes available?

During the research project, new information about the risks and benefits of the study may become known to the researchers. If this occurs, you will be told about this new information and the doctor will discuss whether this new information affects you.

If any information becomes available which could affect your participation in the study the research doctor will tell you about it and discuss whether you want to continue in the study. If you decide to not continue in the study, the research doctor will make arrangements for your care to continue as normal. If you decide to continue in the study you will be asked to sign an updated consent form.

Also, on receiving new information the research doctor might consider it to be in your best interests to withdraw you from the study. He/she will explain the reasons and arrange for your care to continue.

If the study is stopped for any other reason, you will be told why and your continuing care will be arranged.

What will happen if I don't want to carry on with the study?

You can withdraw from the study at any time without giving an explanation and be assured that it will not impact on any part of your further treatment.

If you decide to withdraw from the study, the researchers would like to keep your health information that has already been collected. This is to help them make sure that the results of the research can be measured properly. If you do not want them to do this, you can tell them when you withdraw from the research project.

What if there is a problem?

If you have any concerns regarding the study, please ask to speak to the ICU doctor in charge of your care or ask to speak to name of local PI, the consultant who is in charge of the study.

Complaints:

If you have a concern about any aspect of this study, you should ask to speak with the researchers or the Intensive Care doctors and nurses, who will do their best to answer your questions. If you remain unhappy and wish to complain formally, you can do this through the NHS Complaints Procedure. Please localise with your hospital PALS contact details.

Harm:

In the event that something does go wrong and you are harmed during the research study there are no special compensation arrangements. If you are harmed and this is due to someone's negligence then you may have grounds for a legal action for compensation against University Hospital Southampton, but you may have to pay the legal costs. The normal National Health Service complaints mechanisms will still be available to you.

Involvement of the General Practitioner/Family doctor (GP)

If you are agreeable we would like to inform your GP of your participation in the study. If you do not wish for your GP to be informed, please let us know and indicate on the consent form that you do not wish your GP to be informed.

Will taking part in this study be kept confidential?

If you continue with the study, some parts of your medical records and the data collected for the study will be looked at by authorised researchers from University Hospital Southampton and University of Southampton who are sponsoring and organising the research. They may also be looked at by authorised people to check that the study is being carried out correctly. All will have a strict duty of confidentiality to you, as a research participant and we will do our best to meet this duty.

All information that is collected about you during the course of the research will be kept strictly confidential. Any information about you that leaves the hospital will have your name and address removed so that you cannot be recognised from it.

Anonymised data collected during the study may be sent to associated researchers in other countries, where the laws don't protect your privacy to the same extent as the law in the UK but the study team will take all reasonable steps to protect your privacy.

You have the right to check the accuracy of data held about you and correct any errors.

What will happen to the results of the research study?

They will be published in a medical journal, presented at conferences and lay press where possible.

Who is organising and funding the research?

Dr Rebecca Cusack from University Hospital Southampton is the lead researcher, who is organising the research.

Cusack R, *et al. BMJ Open* 2022; 12:e055285. doi: 10.1136/bmjopen-2021-055285

The research is funded by the NHS through the National Institute for Health Research, Research for Patient Benefit scheme.

Who has reviewed the study?

Hampshire Research Ethics Committee (IRAS number: 250165) have reviewed this study and given their approval.

Thank you very much for taking the time to read this information sheet at this very stressful time.

If you have any further questions please ask the doctors in Intensive Care, Dr (local PI) or one of the research team.

If you agree to continuing participation in this study, please keep this information sheet and you will be given a copy of the agreement form that you will be asked to sign.
