# Supplementary material for: Improving physical function of patients following intensive care unit admission (EMPRESS): protocol of a randomised controlled feasibility trial
Source: BMJ Open. 2022 Apr 15;12(4):e055285. doi: 10.1136/bmjopen-2021-055285 (PMC9014051; doi:10.1136/bmjopen-2021-055285)
Supplement: Supplementary data [file bmjopen-2021-055285supp002.pdf]

FORM TO BE ON SITE SPECIFIC HEADED PAPER

University Hospital Southampton NHS
NHS Foundation Trust

Critical Care, Anaesthesia & Peri-operative Medicine Dept,
Research office CE 93. MP 24,
University Hospital Southampton,
Tremona Road,
Southampton
SO16 6YD

Tel: 023 8120 5308
Fax: 023 8120 5378

Consent form for patients participating in EMPRESS. A feasibility study of early mobilisation programmes in Critical Care.

Name of Researcher: ___________________________

Please initial box

1. I confirm that I have read and understood the Patient Information Sheet (version ___ Dated ___________) for the EMPRESS study. I have had the opportunity to ask questions about the study and understand what is involved. ☐

2. I have no objection to taking part in the above study. ☐

3. I understand that my participation is voluntary and that I am free to withdraw from the study at any time, without giving any reason and without my care or legal rights being affected. ☐

4. I understand that relevant sections of my medical notes and data collected during the study, may be looked at by individuals from the research team, from regulatory authorities or from the NHS Trust, where it is relevant to my taking part in this research. I give permission for these individuals to have access to my records. ☐

5. I understand that information held by the NHS and records maintained by the NHS Information Centre and the NHS Central Register may be used to help contact me and provide information about my health status. I give permission for this information to be obtained and stored by the study research team to enable long term follow-up. ☐

6. I agree to my GP being informed of my participation in the study. ☐

7. I agree to take part in the above study ☐

**Name of Participant:**	**Signature:**	**Date:**
___________________________	___________________________	______________
**Person undertaking consultation (researcher):**	**Signature:**	**Date:**
___________________________	___________________________	______________

Original *Informed Consent form to be filed in the Investigator Site File.*

1 copy to be given to the patient

1 copy to be filed in the patients' hospital notes.

Cusack R, *et al. BMJ Open* 2022; 12:e055285. doi: 10.1136/bmjopen-2021-055285
