# Supplementary material for: Improving physical function of patients following intensive care unit admission (EMPRESS): protocol of a randomised controlled feasibility trial
Source: BMJ Open. 2022 Apr 15;12(4):e055285. doi: 10.1136/bmjopen-2021-055285 (PMC9014051; doi:10.1136/bmjopen-2021-055285)
Supplement: Supplementary data [file bmjopen-2021-055285supp003.pdf]

Site specific header to be inserted here

EMPRESS.

A feasibility study of early mobilisation in Critical Care.

Information for Consultee

Version 2: 29th January 2019

<u>Introduction</u>

Study Title: EMPRESS: A study of very early mobilisation in Critical Care.

Invitation: This hospital is taking part in a national research study to investigate whether starting rehabilitation in the Intensive Care Unit, as soon as possible, will improve patients' long-term physical ability and quality of life.

When patients are sedated in the Intensive Care unit, muscle wasting and weakness can occur very quickly and this can take a long time to recover from. Because we feel that it may be important to deliver rehabilitation physiotherapy as early as possible, we wish for your relative/friend to participate in the trial.

Because your relative/friend is unable to decide for himself/herself whether to participate in this research, we'd like to ask your opinion as to whether or not they would want to be involved. Please consider what you know about their wishes and feelings and what you think may be best for them.

If we have been unable to contact you, your relative/friend may have been enrolled as a participant in this research project with the approval of their treating doctor and the Hampshire Research Ethics Committee (IRAS number: 250165). If this is the case, then we seek to confirm that you are in agreement.

Knowing what is involved will help you decide if you want your relative/friend to continue to take part in the research, so this information sheet explains the tests and treatments involved.

- Part 1 tells you about why we are doing this study and what will happen to your relative if they take part.
- Part 2 gives you more detailed information about how we will run the study.

If you decide your relative/friend would have no objection to taking part, we will ask you to read and sign a form that records your permission, called the consultee declaration. We'll then give you a copy to keep. We will keep you fully informed during the study so you can let us know if you have any concerns or you think your relative/friend should be withdrawn. Taking part in this research is entirely voluntary. If you decide not to continue, they will still be offered the best possible standard of care.

Please ask us if there is anything that is not clear or if you would like more information. Take time to decide whether or not you wish your relative/friend to take part.

PART ONE: Why are we doing this study and what will happen to my friend / relative?

What is the purpose of this study?

We know that when patients are very unwell and need sedation in the Intensive Care Unit, they can lose muscle strength and size very quickly. It is normal to offer rehabilitation, but this often starts after the patient has woken up. By this time the muscles have already been affected. Previous studies have shown that this muscle weakness may take many months to recover from and may affect a patient's quality of life after leaving hospital.

In Southampton Hospital, researchers and physiotherapists started performing rehabilitation exercises much earlier than usual, even while the patient was sedated. They showed that this method reduced the patient's time on the ventilator and reduced the amount of time that they needed to be in Intensive Care.

We are now trying to discover whether this method will work in a number of different hospitals in the UK. We will also do some tests to see whether the patients who have this type of rehabilitation are stronger and able to engage in physical activity more easily, when they leave hospital and 3 months later.

Why has my relative been chosen?

Your relative has been enrolled in this study because during their admission to the Intensive Care Unit he/she needed a ventilator (a machine to help them breathe) and sedation to help keep them calm and comfortable. The treating doctor and physiotherapist thought that either very early rehabilitation or standard rehabilitation would be equally suitable. We may have made a start already, because we are testing such very early rehabilitation, but we would like to ask for your permission to continue.

Does my relative/ friend have to take part?

No. It is up to you to decide whether or not you would like him/her to continue to take part. If you decide they can, you will be given this information sheet to keep and be asked to sign a permission form. You are still free to withdraw your relative at any time without giving a reason. The decision to withdraw at any time, or a decision not to take part, will not affect the standard of care your relative receives.

At an appropriate time, when we hope your relative has recovered sufficiently, we will ask their permission to use the data we have collected. If they do not agree we will not collect any new data and ask if we may use the data already collected.

What will happen to my relative if they take part?

Participation in the study will begin in the Intensive Care Unit. The final tests will take place 3 months after they leave hospital.

In the Intensive Care Unit: Your friend/ relative's treating doctor has assessed them to be eligible to take part in this study: EMPRESS. They were randomly allocated (like the flip of a coin) to receive either of the following:

- **Standard physiotherapy:** All patients on the trial will receive their normal physiotherapy. This will normally include activities to assist in keeping their airway clear and activities to maintain limb flexibility. These will not be affected by being on the trial.

OR

- **Standard physiotherapy, as above, plus an extra 2 sessions of 30 minutes of rehabilitation from Monday to Friday.** For patients receiving extra, early rehabilitation, in addition to their normal physiotherapy, they will start using a cycle machine that is designed to work, in the bed, even with sedated patients. As your friend/ relative wakes up they will start to pedal for themselves, do some more bed-based exercises and finally get out of bed and start moving. All of these sessions will be run by a well-trained physiotherapist and the bedside nurses. We have already tested this method in University Hospital Southampton and it has reduced the length of time on the ventilator and ICU stay. During these sessions, they will be very carefully monitored for their own safety and the safety of their lines, tubes and catheters.

  These exercises will continue for a maximum of 28 days or less if they leave the Intensive care unit before then.

BOTH GROUPS

- **Additional assessments:** So that we can test whether our new method works, patients on the trial will undertake some extra assessments. These include a simple test of grip strength by using a hand held pressure monitor; a test of arm and leg strength, ability to stand and step and mobility and walking tests. There will also be quality of life and health questionnaires.

There was a 50/50 chance of being allocated to either group. Neither you nor their doctor can decide which. No samples of blood are required for this research study.

In the hospital ward: When your friend/ relative has been discharged to a normal hospital ward, they won't receive any extra physiotherapy. Just before they go home, they will be tested again for muscle strength and mobility, including how far they can walk in 6 minutes. These tests will be supervised by a trained and experienced physiotherapist

Following discharge from hospital: Regardless of which group your friend/ relative was allocated to, after going home, they should follow the advice given to them by their doctors and physiotherapists. We have designed our study so that this will not affect our results.

They will be contacted by one of the critical care research team 3 months after they have been discharged home. We will arrange to see them for approximately one hour. During this visit we will test their walking speed, strength and agility. We will also ask for some questionnaires to be completed, which will assess how they feel about their quality of life and recovery.

The researchers would also like to have access to your relative or friend's medical record to obtain information relevant to the study. This information would be anonymised and kept confidential.

Cusack R, *et al. BMJ Open* 2022; 12:e055285. doi: 10.1136/bmjopen-2021-055285

If you have any questions regarding the trial procedures, please don't hesitate to ask the intensive care or research doctors, physiotherapists and nurses.

What do I have to do?

It is important to tell the doctor and the research staff about any treatments or medications you know your relative/friend may have been taking, including over-the-counter medications, vitamins or herbal remedies, acupuncture or other alternative treatments. We would also like to know about any medical conditions which may affect the exercise.

Please just let us know if your relative/friend is involved in any other studies at this time.

What are the alternatives to participation?

Participation in this research is not the only option. You may decide for your relative to receive only standard care physiotherapy. That is absolutely fine. Please feel free to discuss these options with your relative's doctor before deciding whether or not to continue to take part in this research project.

What are the possible disadvantages of taking part?

Early mobility within ICU is safe. Potential risks may include, but not be limited to blood pressure or heart rate problems, breathing problems, problems with the tubes, lines and catheters.

In a review of physiotherapy within Intensive Care Units, involving over 1100 patients and 5267 episodes of physiotherapy in similar patients, there were 34 potential safety events (equal to 6 events in 1000 episodes of physiotherapy). Most of these were related to changes in heart rate or blood pressure and settles quickly on stopping the physiotherapy.

In Southampton, over a 4 year period, we have treated over 500 patients in this way and had 2 events needing attention but neither resulted in harm to the patient..

The doctors, physiotherapists and nurses who will be caring for your relative or friend while in the ICU, are trained to recognise the effects on the body associated with physical rehabilitation and will treat accordingly. Your friend/ relative will be continually monitored and assessed. Their safety will always be our number one priority.

There may be side effects that the researchers do not expect or do not know about and that may be serious. Please tell the doctor immediately if you are worried about any new or unusual symptoms that your relative/friend gets.

What are the possible benefits of taking part?

We cannot guarantee or promise that your relative will receive any benefits from this research. This study aims to further medical knowledge and may improve future treatment of patients who need to be on a ventilator, however it may not directly benefit your relative/friend.

For how long will my relative/ friend be in the research study?

The final research assessment will take place 3 months after discharge from hospital. Once that is done, your friend/ relative's participation in the study will end.

What happens if there is a problem?

We will keep you and your friend/ relative, fully informed of any problems which may be related to the study.

Will taking part in the study be kept confidential?

Yes. All of the information about participation and the data collected will be kept confidential.

Information held by the NHS and records maintained by the NHS Information Centre and the NHS Central Register may be used to help contact your friend/ relative and provide information about their health status. This information may be obtained and stored by the study research team to enable long term follow-up.

University Hospital Southampton is the sponsor for this study based in the United Kingdom. We will be using information from their medical records in order to undertake this study and will act as the data controller for this study. This means that we are responsible for looking after their information and using it properly. University Hospital Southampton will keep information about them for 10 years after the study has finished.

Your friend/ relative's rights to access, change or move your information are limited, as we need to manage the information in specific ways in order for the research to be reliable and accurate. If they withdraw from the study, we will keep the information that we have already obtained. To safeguard their rights, we will use the minimum personally-identifiable information possible.

You can find out more about how we use information at https://www.hra.nhs.uk/information-about-patients/

[Local NHS site name] will collect information from their medical records for this research study in accordance with our instructions.

(Local NHS site name) will keep name, NHS number and contact details confidential and will not pass this information to University Hospital Southampton. [Local NHS site name] will use this information as needed, to contact your relative/ friend about the research study, and make sure that relevant information about the study is recorded for their care, and to oversee the quality of the study. Certain individuals from University Hospital Southampton and regulatory organisations may look at medical and research records to check the accuracy of the research study. University Hospital Southampton will only receive information without any identifying information. The people who analyse the information will not be able to identify patients and will not be able to find out their name, NHS number or contact details.

[Local NHS site name] will keep identifiable information, including the consent form from this study, for 10 years after the study has finished.

Cusack R, *et al. BMJ Open* 2022; 12:e055285. doi: 10.1136/bmjopen-2021-055285

Contact Details:

Local PI details

Address

Dr local PI: 02381 XXXXXX

Research Nurse: 02381 XXXXXX

ICU: 02381 XXXXXX

PART 2: How we will run this study.

What if relevant new information becomes available?

During the research project, new information about the risks and benefits of the study may become known to the researchers. If this occurs, you will be told about this new information and the doctor will discuss whether this new information affects your relative.

If any information becomes available which could affect participation in the study the research doctor will tell you about it and discuss whether you want your relative to continue in the study. If you decide your relative should not continue in the study, the research doctor will make arrangements for your relative's care to continue as normal. If you decide to allow your relative to continue in the study you will be asked to sign an updated agreement form.

Also, on receiving new information the research doctor might consider it to be in your relative's best interests to withdraw them from the study. He/she will explain the reasons and arrange for their care to continue.

If the study is stopped for any other reason, you will be told why and your relative's continuing care will be arranged.

What will happen if I don't want my relative to carry on with the study?

You can withdraw your relative from the study at any time without giving an explanation and be assured that it will not impact on any part of your relative's further treatment.

If you decide to withdraw your relative from the study, the researchers would like to keep your relative's health information that has been collected. This is to help them make sure that the results of the research can be measured properly. If you do not want them to do this, you can tell them when you withdraw your relative from the research project.

What if there is a problem?

If you have any concerns regarding the study, please ask to speak to the ICU doctor in charge of your friend/ relative's care or ask to speak to (name of local PI), the consultant who is in charge of the study.

Complaints:

If you have a concern about any aspect of this study, you should ask to speak with the researchers or the Intensive Care doctors and nurses, who will do their best to answer your questions. If you remain unhappy and wish to complain formally, you can do this through the NHS Complaints Procedure. (Please localise with your hospital PALS contact details)

Harm:

In the event that something does go wrong and your relative is harmed during the research study there are no special compensation arrangements. If your relative is harmed and this is due to someone's negligence then your relative may have grounds for a legal action for compensation against University Hospital Southampton, but they may have to pay the legal costs. The normal National Health Service complaints mechanisms will still be available to them.

Involvement of the General Practitioner/Family doctor (GP)

If you are agreeable we would like to inform your friend/ relative's GP of their participation in the study. If you do not wish for their GP to be informed, please let us know and indicate on the consent form that you do not wish for their GP to be informed.

Will allowing my relative to take part in this study be kept confidential?

If your relative joins the study, some parts of their medical records and the data collected for the study will be looked at by authorised persons from University Hospital Southampton and University of Southampton who are sponsoring and organising the research. They may also be looked at by authorised people to check that the study is being carried out correctly. All will have a strict duty of confidentiality to your relative as a research participant and we will do our best to meet this duty.

All information that is collected about your relative during the course of the research will be kept strictly confidential. Any information about your relative that leaves the hospital will have their name and address removed so that they cannot be recognised from it.

Anonymised data collected during the study may be sent to associated researchers in other countries, where the laws don't protect your privacy to the same extent as the law in the UK but the study team will take all reasonable steps to protect your privacy.

Your relative has the right to check the accuracy of data held about them and correct any errors.

What will happen to the results of the research study?

They will be published in a medical journal, presented at conferences and lay press where possible.

Cusack R, *et al. BMJ Open* 2022; 12:e055285. doi: 10.1136/bmjopen-2021-055285

Who is organising and funding the research?

Dr Rebecca Cusack from University Hospital Southampton is the lead researcher, who is organising the research.

The research is funded by the NHS through the National Institute for Health Research, Research for Patient Benefit scheme.

Who has reviewed the study?

Hampshire Research Ethics Committee (IRAS number: 250165) have reviewed this study and given their approval.

Thank you very much for taking the time to read this information sheet at this very stressful time.

If you have any further questions please ask the doctors in Intensive Care, Dr (local PI) or one of the research team.

If you agree to your relative participating in this study please keep this information sheet and you will be given a copy of the agreement form that you will be asked to sign.
