# Supplementary material for: Improving physical function of patients following intensive care unit admission (EMPRESS): protocol of a randomised controlled feasibility trial
Source: BMJ Open. 2022 Apr 15;12(4):e055285. doi: 10.1136/bmjopen-2021-055285 (PMC9014051; doi:10.1136/bmjopen-2021-055285)
Supplement: Supplementary data [file bmjopen-2021-055285supp004.pdf]

FORM TO BE ON SITE SPECIFIC HEADED PAPER

University Hospital Southampton NHS
NHS Foundation Trust

**Critical Care, Anaesthesia & Peri-operative Medicine Dept,**
**Research office CE 93. MP 24,**
**University Hospital Southampton,**
**Tremona Road,**
**Southampton**
**SO16 6YD**

**Tel: 023 8120 5308**
**Fax: 023 8120 5378**

# Consultee declaration form for patients participating in EMPRESS. A feasibility study of early mobilisation programmes in Critical Care.

**Name of Researcher:** ____________________________ Please initial box

1. I ______________________ **[name of consultee]** have been consulted about ______________________ **[name of potential participant]**'s participation in this research project. I have read and understood the Consultee Information Sheet (version ___; Dated ____________). I have had the opportunity to ask questions about the study and understand what is involved. ☐

2. In my opinion he/she would have no objection to taking part in the above study. ☐

3. I understand that I can request he/she is withdrawn from the study at any time, without giving any reason and without his/her care or legal rights being affected. ☐

4. I understand that relevant sections of his/her medical notes and data collected during the study, may be looked at by individuals from the research team, from regulatory authorities or from the NHS Trust, where it is relevant to their taking part in this research. I give permission for these individuals to have access to their records. ☐

5. I understand that information held by the NHS and records maintained by the NHS Information Centre and the NHS Central Register may be used to help contact me or my friend / relative and provide information about their health status. I give permission for this information to be obtained and stored by the study research team to enable long term follow-up. ☐

6. I agree to their GP being informed of their participation in the study. ☐

When completed: 1 for participant; 1 (original) for researcher site file; 1 to be kept in medical notes.
Version 2: 29th January 2019 IRAS ID:250165

Please confirm either:

I confirm that I will act as the personal consultee for: ______________________

Relationship to participant: ______________________

| **Name of consultee:** | **Signature:** | **Date:** |
| --- | --- | --- |
| ______________________ | ______________________ | ______________ |
| **Person undertaking consultation (researcher):** | **Signature:** | **Date:** |
| ______________________ | ______________________ | ______________ |

***Original*** *Informed Consent form to be filed in the Investigator Site File.*

*1 copy to be given to the patient*

*1 copy to be filed in the patients' hospital notes.*

When completed: 1 for participant; 1 (original) for researcher site file; 1 to be kept in medical notes.
Version 2: 29th January 2019 IRAS ID:250165
