# Supplementary material for: Improving physical function of patients following intensive care unit admission (EMPRESS): protocol of a randomised controlled feasibility trial
Source: BMJ Open. 2022 Apr 15;12(4):e055285. doi: 10.1136/bmjopen-2021-055285 (PMC9014051; doi:10.1136/bmjopen-2021-055285)
Supplement: Author's manuscript [file bmjopen-2021-055285.draft_revisions.pdf]

BMJ Open is committed to open peer review. As part of this commitment we make the peer review history of every article we publish publicly available.

When an article is published we post the peer reviewers' comments and the authors' responses online. We also post the versions of the paper that were used during peer review. These are the versions that the peer review comments apply to.

The versions of the paper that follow are the versions that were submitted during the peer review process. They are not the versions of record or the final published versions. They should not be cited or distributed as the published version of this manuscript.

BMJ Open is an open access journal and the full, final, typeset and author-corrected version of record of the manuscript is available on our site with no access controls, subscription charges or pay-per-view fees (http://bmjopen.bmj.com).

If you have any questions on BMJ Open's open peer review process please email info.bmjopen@bmj.com

BMJ Open

Improving physical function of patients following Intensive Care Unit admission (EMPRESS): Protocol of a randomised controlled feasibility trial

Journal:	*BMJ Open*
Manuscript ID	bmjopen-2021-055285
Article Type:	Protocol
Date Submitted by the Author:	11-Aug-2021
Complete List of Authors:	Cusack, Rebecca; University Hospital Southampton NHS Foundation Trust, NIHR Biomedical Research Centre ; University Hospital Southampton NHS Foundation Trust, Department of Intensive Care<br>Bates, Andrew; University Hospital Southampton NHS Foundation Trust, NIHR Southampton Biomedical Research Centre<br>Mitchell, Kay; University Hospital Southampton NHS Foundation Trust, NIHR Biomedical Research Centre<br>van Willigen, Zoe; University Hospital Southampton NHS Foundation Trust, Department of Physiotherapy<br>Denehy, Linda; The University of Melbourne Melbourne School of Health Sciences; Peter MacCallum Cancer Institute<br>Hart, Nicholas; Guy's and St Thomas' NHS Foundation Trust; King's College London, Respiratory and Critical Care<br>Dushianthan, Ahilanandan; University Hospital Southampton NHS Foundation Trust, General Intensive Care Unit; University Hospital Southampton NHS Foundation Trust, NIHR Biomedical Research Centre<br>Reading, Isabel; University of Southampton<br>Chorozoglou, Maria; University of Southampton<br>Sturmey, Gordon<br>Davey, Iain<br>Grocott, Michael; University of Southampton, Faculty of Medicine ; University Hospital Southampton NHS Foundation Trust, NIHR Biomedical Research Centre
Keywords:	Adult intensive & critical care < INTENSIVE & CRITICAL CARE, REHABILITATION MEDICINE, Quality in health care < HEALTH SERVICES ADMINISTRATION & MANAGEMENT

SCHOLARONE™
Manuscripts

Improving physical function of patients following Intensive Care Unit admission (EMPRESS): Protocol of a randomised controlled feasibility trial

Authors:

Rebecca J Cusack[1,2], Andrew Bates[1], Kay Mitchel[1], Zoe van Willigen[2], Linda Denehy[3,4], Nicholas Hart[5,6], Ahilanandan Dushianthan[1,2], Isabel Reading [1], Maria Chorozoglou [1], Gordon Sturmey[7], Iain Davey[7],Michael P W Grocott[1,2]

Author Affiliations

1 University of Southampton UK

2 University Hospital of Southampton NHS Foundation Trust UK

3 University of Melbourne Australia

4 Peter MacCallum Cancer Centre, Melbourne, Victoria, Australia

5 Guys and St Thomas' Hospital NHS Foundation Trust London UK

6 Kings College University of London UK

7 Patient advisory group

Corresponding Author:
Dr Rebecca Cusack
University Hospital of Southampton NHS FT
Tremona Road Southampton
E-mail R.Cusack@soton.ac.uk
Phone: +44 2381 202382

For peer review only

Word Count: 4109

Keywords: Critical care, physical therapy, rehabilitation, early ambulation

Improving physical function of patients following Intensive Care Unit admission (EMPRESS): protocol of a randomised controlled feasibility trial

Introduction: Physical rehabilitation delivered early following admission to the Intensive Care Unit (ICU) has the potential to improve short and long-term outcomes. The use of supine cycling together with other rehabilitation techniques has potential as a method of introducing rehabilitation earlier in the patient journey. The objective of the study is to determine the feasibility of recruitment and delivery of a randomised clinical trial comparing an early mobilisation programme including cycling with usual care to inform a larger multicentred study.

Methods and Analysis: 90 acute medical patients from 2 mixed medical-surgical ICUs will be recruited. We will include within 72 hours of initiation of mechanical ventilation and expected to be ventilated a further 48 hours or more. Patients will receive usual care or usual care plus two 30-minute rehabilitation sessions 5 days per week.

Feasibility outcomes are: i)recruitment 1-2 patients per month per site, ii) protocol fidelity with > 75% of patients commencing interventions within 72 hours of mechanical ventilation, > 70% interventions delivered and iii) blinded outcome measures recorded at 3 time points in > 80% of patients. Secondary outcomes are: i) strength and function; the Physical Function ICU Test-scored (PFITs) measured on ICU discharge, ii) hospital length of stay and iii) mental health and physical ability at 3 months using the WHODAS 2. An economic analysis using hospital health services data reported with an embedded health economic study will collect and assess economic and QoL data.

Ethics and Dissemination: The study has ethical approval from South Central Hampshire A Research Ethics Committee (19/SC/0016). An independent trial monitoring committee is overseeing the study. Results will be made available to critical care survivors, their caregivers, the critical care societies and other researchers.

Trial registration number: NCT03771014

Sponsor: University Hospital Southampton NHS Foundation Trust.

Strengths and limitations of study

- Will investigate the implementation of an early mobilisation intervention, which is usual care in one NHS/University Teaching institution, in other NHS institutions with different structures
- The defined cohort has been demonstrated to benefit from this type of rehabilitation in alternative health care systems

For peer review only

- Results will inform the design of a multi-centred RCT
- This study is not designed to assess effectiveness of the intervention
- Inability to blind the intervention to patients, physiotherapist and clinicians involved in the study.

Introduction

In 2018/19 there were over 290,000 admissions to adult intensive care units (ICU) in the United Kingdom [1]. Treatment advances have reduced mortality associated with critical illness [2, 3], however, survival does not represent the end of the story[4]. A complex interplay between baseline health status, acute disease and the traumatic effects of intensive care treatment is associated with long-term physical, psychological and social hardship with cognitive impairment and substantially reduced quality of life [5-10]. Within the UK, patients discharged from ICU have a higher mortality, higher health service costs and a 50% reduction in employment in the 5-years following discharge, compared to hospitalised patients not requiring ICU [8, 11].

ICU acquired weakness (ICU-AW) is characterised by rapid muscle wasting, polyneuropathy and bone demineralisation, causing pain, weakness and impaired physical function [12-14]. Risk factors are multifactorial although immobility principally due to the sedation required for tolerance of ventilation plays an important role [15, 16]. Efforts to mitigate these consequences have included a move towards earlier mobilisation of critical care patients [17, 18], defined as commencing within 5 days of admission to the ICU [19]. A seminal RCT of early mobilisation intensive care patients in 2009 found patients who received early physical therapy (within 1.5 days of mechanical ventilation) had greater functional independence at hospital discharge than the patients who received usual care physical therapy commencing 7.4 days mechanical ventilation (59% vs 35% p=0.02)[20]. While meta-analyses and systematic reviews report that early rehabilitation and mobilisation of ICU patients improves short term physical outcomes[21-23] a number of studies with a delayed start of rehabilitation have not had similar outcomes [24-30]. Physical rehabilitation is difficult to implement early during a patient's stay in the ICU, and often delayed beyond a week after ICU admission [31-33]. Contributary barriers can be attributed to patient factors such as safety concerns, heavy sedation or agitation and organisational factors such as resources and culture within an individual unit [34]. A number of studies report the feasibility and safety of using cycle ergometry in critically ill patients [35-37] .In-bed cycle ergometry can facilitate passive activity in the acute phase of illness in patients who are heavily sedated and receiving vasopressors [38 39] with minimal physiological demand [39 40] and transition to active cycling as the patient's condition improves. Early cycle ergometry has been shown to preserve muscle cross sectional area in patients presenting with septic shock[41], and greater increase in muscle strength in patients receiving passive cycling [42] However recent systematic reviews do not conclusively report

For peer review only

find any differences in physical function, duration of mechanical ventilation, ICU and hospital length of stay in patients who received cycle ergometry in ICU [43,44].

As a Quality Improvement process, we introduced cycle ergometry as part of an early mobility programme which included employing physiotherapy technician to support the additional workload involved [45]. Like other investigators our intervention reduced both number of ventilator days and ICU length of stay indicating potential of cost effectiveness [46-49]. The benefits of such early mobility programmes are supported by a recent RCT on the impact of a progressive ICU mobility programme which found that patients who had a progressive mobility programme in addition to usual care had better functional status at discharge from the ICU [50]

The primary aim of this study is to establish feasibility and trends in efficacy to support a prospective, randomised, multi-centre study in the UK is needed to determine if early mobilisation including if cycling in ICU confers patient benefit. This protocol is reported according to SPIRIT [51] and TIDieR[52] guidelines.

Aim and Hypotheses

EMPRESS is a randomised feasibility study which aims to assess if an early mobilisation programme that includes cycling can be delivered, with follow-up assessments, in two NHS Intensive Care Units in the UK. We hypothesise that early rehabilitation with cycling will be successfully carried out in critically ill patients in ICU with acceptable intervention fidelity.

METHODS AND ANALYSIS

Study design:

This is a two-centre feasibility study using a two-arm RCT, randomised 1:1, with blinded outcome assessments at ICU discharge, hospital discharge and 3-month follow-up. Participants will be recruited from two general intensive care units, located in the south of the UK. Each site will have a principal investigator from the NIHR Clinical Research Network, experienced in delivering clinical trials.

Participants:

Ninety participants meeting eligibility criteria will be recruited. Eligible patients will be over 42 years old and have an acute/unplanned medical admission to the ICU. They will be functionally independent prior to ICU admission (Barthel Index >80), in hospital for <5 days prior to intubation and ventilation, intubated and ventilated for <72 hrs and expected to remain ventilated for a further 48 hours. Patients will be excluded if in hospital for 5 days or more prior to ICU admission, have acute brain or spinal cord injury, known or suspected neurological / muscular impairment, condition

For peer review only

limiting use of cycle ergometry (e.g. lower limb fracture / amputation), not expected to survive >48hrs decided by consulting Intensivist, persistent therapy exemptions in first 3 days of mechanical ventilation. (Figure 1) presents the planned flow of patients through the study.

Recruitment, consent and randomisation:

ICU researches will screen all patients for trial eligibility. Recruitment began in June 2019 (and was temporarily suspended in March 2020 due to the COVID 19 pandemic). It is anticipated recruitment will continue until early 2022. The majority of participants will have diminished capacity, therefore, the consent process is multi-layered and designed in accordance with the Mental Capacity Act (MCA) 2005 [53] (Figure 2). .*Patient Informed Consent*: Wherever possible, informed consent will be directly sought from the patient. *Personal Consultee Informed Assent*: If the participant is unable to provide consent, informed assent will be sought from the patient's personal consultee, within 6 hours of confirmation of eligibility. If the personal consultee is not available in person, attempts will be made to contact them by telephone. They will be asked to provide written assent, at the earliest possible convenience. *Professional Consultee Informed Assent:* Where both patient and personal consultee are not available to approve enrolment within 6 hours of confirmation of eligibility, assent will be sought from a professional consultee in accordance with the MCA. The professional consultee will be a consultant medical practitioner, independent from the study. The patient's personal consultee will be consulted at the earliest possible opportunity and assent requested to continue in the study.
In all cases, once the participant has regained capacity they will be informed of the study and consent continuation sought. Following consent or assent, patients will be registered on a bespoke electronic data collection tool (ALEA™) and randomly assigned to early mobilisation or usual care.

Staff Training/ site set-up:

Participating sites will employ the equivalent of a full-time therapy technician to deliver the study intervention, under the supervision of a senior critical care therapist. Both senior critical care therapists and therapy technicians will complete a training package delivered by the primary institution (University Hospital Southampton), where early rehabilitation with cycling is well established and embedded in usual care. This includes seminars on the delivery of early mobilisation, use of the bespoke electronic database and 5-days of clinical shadowing. An electronic copy of the full training program used at the primary institution has been given to the study sites for reference. The manufacturer supplied additional training on use of the cycle ergometer.

Interventions:

All participants will receive usual medical, nursing and physiotherapy care while in intensive care. Each bedside nurse will be asked at the start of the shift if they have been involved caring for a patient

For peer review only

in the intervention arm of the study. The ICU physiotherapy team, who are not involved in delivery of the study intervention, will deliver all usual physiotherapy interventions in both groups. The physiotherapist delivering usual care will be asked to verify if they have delivered any of the study interventions. At the start of each physiotherapy intervention the participants level of sedation will be assessed using the Richmond Agitation-Sedation Scale (RASS) [54 55] and the Confusion Assessment Method for ICU (CAM-ICU) [56]. Sedation will be targeted to a RASS between -1 and +1 by the bedside nurse. After 28 days of ICU admission, all participants will receive usual care physiotherapy interventions.

Group 1: Usual care control group

Participants will receive physiotherapy interventions guided by individual assessment prior to each intervention. This includes, where appropriate, passive or active range of movement (PROMs), positioning and respiratory physiotherapy, and when able, sitting on the edge of the bed, standing (assisted or unassisted), standing to transfer to chair, marching on the spot and walking . (Figure 3).

Group 2: Early mobilisation pathway

Participants will receive usual care physiotherapy, in addition to commencing the early mobilisation pathway within 72 hours of ICU admission. Participants will be screened for criteria to withhold the intervention prior to each planned intervention session(Table 1).

For peer review only

Table 1 Safety criteria for delivery of physical therapy interventions

	Criteria to commence physiotherapy	Criteria to stop / withhold physiotherapy intervention
Blood pressure	MAP 60 – 100 mmHg, no change in vasopressor dose requirement for preceding 2 hours	Catecholamine resistant hypotension with MAP < 60 mmHg
Heart rate	Between 40-140 bpm	<50 or >140 bpm
Respiratory rate	Sustained < 40 breaths/min	Sustained >40 breaths/min
Temperature		>40 °C
Oxygen requirement	If FiO2 >0.8 for passive exercise only<br>FiO2 <0.8 and PEEP<15 cmH_2O	
Desaturation		Sats fall <85% for > 1 minute
Other		· Fall<br>· Unplanned extubation<br>· Acute bleeding<br>· New onset arrhythmia<br>· Signs/symptoms of acute myocardial ischaemia<br>· Patient pain/distress<br>· Clinical team decide therapy intervention not appropriate<br>· Refusal by patient or representative

Those meeting criteria to withhold intervention will have issues addressed and will be reassessed for intervention 2 hours later. Usual physiotherapy will be delivered by the ward physiotherapists. Additional mobilisation sessions will be delivered by the research physiotherapy staff. This will initially comprise one additional mobility session, chosen at the discretion of the physiotherapist, plus one 30-minute session of supine cycling.

The first mobilisation intervention each day will include activities such as PROMS, passive cycling, active cycling, in bed exercises, sitting, mobilisation out of bed and walking. Daily assessment of the patient will be made to ensure the highest level of activity is provided for each individual patient.

The second session will be cycling based. We will use an in-bed supine cycle ergometer (MotoMed

Letto 2™) to engage in passive, assisted or active cycling, or a combination, depending on the degree of patient co-operation (Figure3). The aim is for the patient to have 30 minutes of cycling per day, following a standardised cycling programme. If cycling is in passive mode, they commence cycling at 5 revolutions per minute (RPM), building up to 20 RPM over a 5-minute period and continue this for 20 minutes before 5-minute 5RPM cool down. In the assisted or active mode, after the 5-minute warm up, cycling will continue for 20 minutes at patient selected RPM followed by a 5-minute cool down at 5 RPM. In-bed cycling sessions will stop when the patient is deemed to be able to stand and transfer from bed to chair for both mobility sessions for two consecutive days. If participants are considered unable to have concurrent mobility therapy and respiratory weaning, mobility therapy will take priority, in agreement with the senior clinical team. Individual participants will receive the trial intervention on five days per week (Monday to Friday) for the duration of their ICU stay or maximum of 28 days whichever comes first. Participants will be monitored for cardiovascular and respiratory stability and safety of indwelling lines, tubes and catheters with pre-determined criteria for termination of any session (Table 1). Deviations from the planned protocol will be reported to determine potential barriers to implementation. Participants will be able to decline any intervention or outcome assessment at any time without compromise to their care.

Feasibility outcomes: Primary Outcomes

Feasibility will be determined by measures of the recruitment process, intervention fidelity and outcome measurement completeness, specifically:

1. Study accrual rates: a minimum of 30% of eligible patients or 1-2 patients per site per month are enrolled
2. Protocol adherence: 75% of patients commencing intervention within 72 hours of ICU admission; minimum of 70% of planned interventions delivered
3. Blinded outcome assessment: functional assessment performed at 3 time-points in 80% of survivors.

Secondary Outcomes:

The schedule of outcome assessments is detailed in Table 2

For peer review only

Strength and Function

We will measure the Physical Function ICU Test-scored (PFITs) measured at awakening as described by deJonghe [57] then weekly within ICU and on ICU discharge [58]. PFITs is a reliable and valid 4 item scale (arm strength, leg strength, ability to stand and step cadence), with a score range of 0-10 and is responsive to change and predictive of key outcomes [59]. Medical Research Council Manual Muscle Test Sum Score (MRC-ss) [60 61] and Hand Held Dynamometry (HHD)[62] will be measured on awakening, weekly, on ICU discharge and hospital discharge. Chelsea Critical Care Physical Assessment tool (CPAX) [63] and ICU Mobility Scale [64] will be assessed three times during the first week within ICU, on awakening, weekly thereafter within the ICU and at ICU discharge. Timed Up and Go (TUG)[65 66], Clinical Frailty Score (CFS)[67-69] and Barthel Index will be assessed at ICU discharge, hospital discharge and 3-months post-hospital discharge. Pre-admission Barthel Index and CFS will be assessed by proxy on admission from family member or next of kin. Six-minute walk test (6MWT) [70] will be performed, in accordance with American Thoracic Society guidelines, at hospital discharge and 3-months post-hospital discharge.

Health related quality of life Outcomes

The following will be measured at 3-months post-hospital discharge : WHODAS-2.0 [71] , Hospital Anxiety and Depression Score (HADS) [72 73] , Euroqol-5 Dimension-5Level (EQ-5D-5L)[74], Impact of Event Score (IES) [75] and Client Service Receipt Inventory questionnaire (CSRI), designed for this study to evaluate costs that fall on patients and their carers. Resource use and costs including direct intervention costs of therapists and equipment and General Hospital costs (per bed day) will be recorded for each patient

Health economic sub-study

Alongside the feasibility RCT we will conduct an embedded health economic study with the aim to identify and define data collection for the future RCT where a full cost effectiveness analysis (CEA) will be conducted. Within the feasibility study we aim to address the following research questions: what is the quality of the data and potential problems reporting QoL (EQ-5D-5L), resource use and costs; what are the cost implications of the proposed intervention in terms of impact for the NHS (inpatient stay bed days) and identifying the main cost drivers; is the EQ-5D-5L appropriate for use in the future RCT. The economic outcomes will include: secondary care resource use from hospitals during inpatient stay, primary care resource use following discharge up to 3m and resource use providing the intervention. The results will be reported in the form of descriptive statistics and will be used to inform a future CEA within a definitive RCT.

Additional data collection

For peer review only

We will collect baseline data including demographic information, Functional Comorbidity Index, ICU diagnosis, APACHE II score, ventilation duration, ventilator free days, ICU and hospital length of Stay, within ICU drug history and duration and type of usual care physiotherapy.

Implementation Evaluation

We aim to investigate whether the early mobilisation programme used in one NHS institution is transferable, as an RCT, into other similar NHS health institutions. The design of a future multi-centre study will be informed by identified facilitators and barriers to implementation. Implementation assessment will be based on the measures described by Proctor [76]. A cross section of ICU staff and patients will complete questionnaires at trial completion by direct questioning and use of questionnaires. Understanding of the integration and sustainability of the intervention are necessary to inform the design of a powered RCT. Acceptability will be measured at the beginning and end of the study from investigators and clinical staff by direct discussions and questionnaire. Our experience informs us that the introduction of this intervention is dependent on a cultural change within any unit for a pro-active focus on early mobilisation. We aim to explore measures to help optimise implementation. Adoption, feasibility and fidelity measures will be monitored during the study by regular meetings with the investigators. Patient screening logs will identify the number of patients eligible for the study and barriers to enrolment. We will assess the degree to which it is possible to separate the staff caring for the intervention group from those caring for the patients in the control group.

We will report whether trial participation has influenced usual care within the participating units by pre- and post-study audits. Participating sites will collect data regarding number and seniority of therapy staff with dedicated time to work within the ICU; delirium and sedation protocols used; time, type and frequency of rehab interventions delivered, who delivers the interventions and reasons why usual care may not be delivered.

The feasibility outcomes are described above and will be used to power a full randomised control trial.

Data entry and checks

Data will be entered into the electronic case report form (ALEA™) and data validation will take place according to the procedures set out in the data management plan and data validation plan, both developed apriori. Missing data will be assessed to identify any specific challenges with any items of data collected. Missing data level expected to be less the 20%. Data loss and mortality will inform number of participants needed to design a larger randomised trial. As this is a feasibility

For peer review only

study data imputation will not be undertaken. Prior to statistical analysis, variables will be checked for missing and impossible and improbable values as defined by clinical opinion. Questions regarding the data will go to the data manager.

Sample size calculation

This is a feasibility study the results of which will be used to power a definitive study if appropriate, as such no formal sample size calculation has been undertaken. A total of 90 participants will be recruited to this study aiming for 30-45 participants at each site. We anticipate a 30% in hospital mortality /loss to follow-up with an estimated total of 60 patients completing the study. This sample size will provide enough data to be representative of the population of ICU patients requiring rehabilitation.

Statistical analysis

The analysis will be reported in line with the feasibility studies extension to the CONSORT statement [77]. The main aims of the study are to estimate the recruitment, compliance and retention rates to inform the design of a future study and is not powered for hypothesis testing regarding the effectiveness of the intervention. Baseline and demographic characteristics of randomised participants will be summarised and the two groups compared to ensure balanced recruitment. Mortality and participant drop out will be examined. Primary and secondary outcome measures will be presented using summary statistics using means and standard deviations or medians and ranges/interquartile ranges, as applicable.

Trial management

The Chief Investigator will ensure all study personnel are appropriately orientated and trained, oversee recruitment and report to the trial safety monitoring committee. Training will occur across sites using competency based training developed at the primary site (University Hospital Southampton). A study steering group, consisting of an independent chair, expert members and 2 lay advisors will meet every 3-months. Fortnightly teleconferences with trial sites will be held to monitor conduct and progress. Timing and intervals of visits and teleconferences will be reviewed at 3 months to ensure optimal time use.

The CI and PIs will facilitate local monitoring by the R&D quality manager, REC review and provide access to source data as required. A monitoring report will be produced, summarising the visit, documents and findings. The CI will ensure that all findings are addressed appropriately. The steering group will review all events in a timely manner. Additional monitoring will be scheduled where there is evidence or suspicion of non-compliance with the Study protocol.

For peer review only

A Data Management and Safety Committee will be chaired by an independent expert. Quarterly reports will be given to the committee once recruitment has commenced.

Patient and Public Involvement

The study has been supported by patient advisory representatives. These representatives are members of the trial steering committee. Patient advisors partnered with us for the design of the study, the informational material to support the intervention, the burden of the intervention from the patient's perspective and contributed to the dissemination plan

Ethics and dissemination

Ethical approval has been granted by South Central - Hampshire A Research Ethics Committee (REC reference 19/SC/0016). EMPRESS was registered with clinicaltrials.gov NCT03771014 on December 10th 2018.

Results of this proposed feasibility study will be disseminated for four key audiences: i) patients and public; ii) Intensive care staff, healthcare workers and potential future research delivery partners; iii) service delivery organisations and iv) academic and potential future research collaborators. Dissemination activities will include: Feedback to PPI study focus group, feedback to study participants, presentations to local clinical teams and managers and commissioners and presentation at conferences attended by appropriate healthcare professionals. Where appropriate, results will be published in peer reviewed journals.

Safety and adverse events

Early mobility within ICUs is safe. In a review of physiotherapy in a critical care rehabilitation programme of 2.5years, 1110 patients received 5267 rehabilitation sessions physiological abnormalities or potential adverse events occurred in only 6 per 1000 interventions [78]. Of these patients 628 intervention sessions included in-bed cycling with 1 safety event. Mobilisation interventions will only be delivered if patients fit the safety criteria defined in table 1. Similar safety criteria have been used in other ICU rehabilitation studies[79 80] .

All interventions will be documented. Any intervention will cease according to stopping criteria detailed in table 1. Any such event will be recorded as an adverse event. The Chief Investigator will provide a monthly update to the safety monitoring committee. Serious adverse events are events that result in death, are life threatening or require prolonged hospitalisation. Any such event will be reported in accordance with the NHS Health Research Authority guidance.

Discussion:

For peer review only

Empress is a feasibility study to determine if an early mobilisation programme, that includes cycling can be delivered in ventilated patients, with blinded follow-up assessments. It is not powered to determine any potential effectiveness or cost-effectiveness of the early mobility programme described however the results of this study will inform the design of a future multi-centre RCT. In-bed cycle ergometry circumvents the need for volitional engagement from the patient enabling our physiotherapy interventions to commence very soon after the patient's admission to intensive care. The protocol facilitates early initiation of the intervention, commencing when the patient is physiologically stable but may still be heavily sedated and receiving vasopressors, with progression from passive to active in-bed cycling and then to out of bed mobility activities as the patient becomes more engaged. Due to the increased workload of delivering the additional physiotherapy sessions, the physiotherapy team will be supported by a full-time therapy technician to the therapy team. Economic and implementation evaluations will determine cost effectiveness and identify challenges that will need to be considered in the design of a future larger study.

Acknowledgements

Contributions: RJC and AB contributed equally to the preparation of the paper. RJC and ZvW had the original idea for the study, RJC, LD, IR, NH, AD, GS, ID, MPWG developed the trial protocol, IR devised the statistical analysis plan, MC developed the economic analysis. AB, GS, ID and RJC prepared and submitted documents for Research and Development and ethical approval. RJC, KM and AB wrote the manuscript. All authors reviewed the final version.

Funding: This paper presents independent research funded by the National Institute for Health Research (NIHR) under its Research for Patient Benefit (RfPB) Programme. Grant Reference Number PB-PG-0317-20045. KM is an NIHR 70@70 Senior Research Leader

Competing Interests: None Declared

Ethics Approval: The study has ethical approval from South Central Hampshire A Research Ethics Committee (19/SC/0016).

Provenance and Peer review: Not commissioned. Protocol peer-reviewed for ethical and funding application

Disclaimer: The views expressed are those of the author(s) and not necessarily those of the NIHR,

For peer review only

NHS or the Department of Health and Social Care.

References:

1. Hospital Admitted Patient Care Activity 2018-19: NHS England; 2019 [National stastics]. Available from: https://digital.nhs.uk/data-and-information/publications/statistical/hospital-admitted-patient-care-activity/2018-19.
2. Esteban A, Frutos-Vivar F, Muriel A, et al. Evolution of Mortality over Time in Patients Receiving Mechanical Ventilation. *American Journal of Respiratory and Critical Care Medicine* 2013;188(2):220-30. doi: 10.1164/rccm.201212-2169oc
3. Kaukonen KM, Bailey M, Suzuki S, et al. Mortality Related to Severe Sepsis and Septic Shock Among Critically Ill Patients in Australia and New Zealand, 2000-2012. *Jama-J Am Med Assoc* 2014;311(13):1308-16. doi: 10.1001/jama.2014.2637
4. Iwashyna TJ, Netzer G. The Burdens of Survivorship: An Approach to Thinking about Long-Term Outcomes after Critical Illness. *Seminars in Respiratory and Critical Care Medicine* 2012;33(4):327-38. doi: 10.1055/s-0032-1321982
5. Cheung AM, Tansey CM, Tomlinson G, et al. Two-year outcomes, health care use, and costs of survivors of acute respiratory distress syndrome. *American Journal of Respiratory and Critical Care Medicine* 2006;174(5):538-44.
6. Cuthbertson BH, Roughton S, Jenkinson D, et al. Quality of life in the five years after intensive care: a cohort study. *Critical Care* 2010;14(1) doi: 10.1186/cc8848
7. Desai SV, Law TJ, Needham DM. Long-term complications of critical care. *Critical Care Medicine* 2011;39(2):371-79.
8. Griffiths J, Hatch RA, Bishop J, et al. An exploration of social and economic outcome and associated health-related quality of life after critical illness in general intensive care unit survivors: a 12-month follow-up study. *Critical Care* 2013;17(3) doi: 10.1186/cc12745
9. Herridge MS, Tansey CM, Matte A, et al. Functional Disability 5 Years after Acute Respiratory Distress Syndrome. *New England Journal of Medicine* 2011;364(14):1293-304. doi: 10.1056/NEJMoa1011802
10. Iwashyna TJ, Ely EW, Smith DM, et al. Long-term Cognitive Impairment and Functional Disability Among Survivors of Severe Sepsis. *Jama-J Am Med Assoc* 2010;304(16):1787-94. doi: 10.1001/jama.2010.1553
11. Lone NI, Gillies MA, Haddow C, et al. Five-Year Mortality and Hospital Costs Associated with Surviving Intensive Care. *American Journal of Respiratory and Critical Care Medicine* 2016;194(2):198-208. doi: 10.1164/rccm.201511-2234OC
12. Allen C, Glasziou P, Del Mar C. Bed rest: a potentially harmful treatment needing more careful evaluation. *Lancet* 1999;354(9186):1229-33. doi: 10.1016/s0140-6736(98)10063-6
13. Jolley SE, Bunnell AE, Hough CL. ICU-Acquired Weakness. *Chest* 2016;150(5):1129-40. doi: 10.1016/j.chest.2016.03.045 [published Online First: 2016/04/07]
14. Parry SM, Puthucheary Z. The impact of extended bed rest on the musculoskeletal system in the critical care environment. *Extreme Physiology and Experimental Medicine* 2015;4
15. Puthucheary ZA, Rawal J, McPhail M, et al. Acute Skeletal Muscle Wasting in Critical Illness. *Jama-J Am Med Assoc* 2013;310(15):1591-600. doi: 10.1001/jama.2013.278481
16. Levine S, Nguyen T, Taylor N, et al. Rapid Disuse Atrophy of Diaphragm Fibers in Mechanically Ventilated Humans. *New England Journal of Medicine* 2008;358(13):1327-35. doi: 10.1056/NEJMoa070447

For peer review only

17. Needham DM. Mobilizing patients in the intensive care unit - Improving neuromuscular weakness and physical function. *Jama-J Am Med Assoc* 2008;300(14):1685-90. doi: 10.1001/jama.300.14.1685
18. Kress JP, Hall JB. CRITICAL CARE MEDICINE ICU-Acquired Weakness and Recovery from Critical Illness. *New England Journal of Medicine* 2014;370(17):1626-35. doi: 10.1056/NEJMra1209390
19. Hodgson CL, Berney S, Harrold M, et al. Clinical review: early patient mobilization in the ICU. *Crit Care* 2013;17(1):207. doi: 10.1186/cc11820 [published Online First: 2013/05/16]
20. Schweickert WD, Pohlman MC, Pohlman AS, et al. Early physical and occupational therapy in mechanically ventilated, critically ill patients: a randomised controlled trial. *Lancet* 2009;373(9678):1874-82. doi: 10.1016/s0140-6736(09)60658-9
21. Calvo-Ayala E, Khan BA, Farber MO, et al. Interventions to Improve the Physical Function of ICU Survivors A Systematic Review. *Chest* 2013;144(5):1469-80. doi: 10.1378/chest.13-0779
22. Hermans G, De Jonghe B, Bruyninckx F, et al. Interventions for preventing critical illness polyneuropathy and critical illness myopathy. *Cochrane Db Syst Rev* 2014(1) doi: 10.1002/14651858.CD006832.pub3
23. Kayambu G, Boots R, Paratz J. Physical Therapy for the Critically Ill in the ICU: A Systematic Review and Meta-Analysis. *Critical Care Medicine* 2013;41(6):1543-54. doi: 10.1097/CCM.0b013e31827ca637
24. McWilliams D, Jones C, Atkins G, et al. Earlier and enhanced rehabilitation of mechanically ventilated patients in critical care: A feasibility randomised controlled trial. *J Crit Care* 2018;44:407-12. doi: 10.1016/j.jcrc.2018.01.001 [published Online First: 2018/01/15]
25. Wright SE, Thomas K, Watson G, et al. Intensive versus standard physical rehabilitation therapy in the critically ill (EPICC): a multicentre, parallel-group, randomised controlled trial. *Thorax* 2018;73(3):213-21. doi: 10.1136/thoraxjnl-2016-209858
26. Denehy L, Skinner EH, Edbrooke L, et al. Exercise rehabilitation for patients with critical illness: a randomized controlled trial with 12 months of follow-up. *Critical Care* 2013;17(4) doi: 10.1186/cc12835
27. Moss M, Nordon-Craft A, Malone D, et al. A Randomized Trial of an Intensive Physical Therapy Program for Patients with Acute Respiratory Failure. *Am J Respir Crit Care Med* 2016;193(10):1101-10. doi: 10.1164/rccm.201505-1039OC [published Online First: 2015/12/15]
28. Walsh TS, Salisbury LG, Boyd J, et al. A randomised controlled trial evaluating a rehabilitation complex intervention for patients following intensive care discharge: the RECOVER study. *Bmj Open* 2012;2(4) doi: ARTN e001475 10.1136/bmjopen-2012-001475
29. Cuthbertson BH, Rattray J, Campbell MK, et al. The PRaCTICaL study of nurse led, intensive care follow-up programmes for improving long term outcomes from critical illness: a pragmatic randomised controlled trial. *BMJ* 2009;339(oct16 1):b3723-b23. doi: 10.1136/bmj.b3723
30. Elliott D, McKinley S, Alison J, et al. Health-related quality of life and physical recovery after a critical illness: a multi-centre randomised controlled trial of a home-based physical rehabilitation program. *Critical Care* 2011;15(3) doi: 10.1186/cc10265
31. Berney SC, Harrold M, Webb SA, et al. Intensive care unit mobility practices in Australia and New Zealand: a point prevalence study. *Critical Care and Resuscitation* 2013;15(4):260-65.
32. Harrold ME, Salisbury LG, Webb SA, et al. Early mobilisation in intensive care units in Australia and Scotland: a prospective, observational cohort study examining mobilisation practises and barriers. *Critical Care* 2015;19 doi: 10.1186/s13054-015-1033-3
33. Nydahl P, Ruhl AP, Bartoszek G, et al. Early Mobilization of Mechanically Ventilated Patients: A 1-Day Point-Prevalence Study in Germany*. *Critical Care Medicine* 2014;42(5):1178-86. doi: 10.1097/ccm.0000000000000149

For peer review only

34. Dubb R, Nydahl P, Hermes C, et al. Barriers and Strategies for Early Mobilization of Patients in Intensive Care Units. *Ann Am Thorac Soc* 2016;13(5):724-30. doi: 10.1513/AnnalsATS.201509-586CME
35. Nydahl P, Sricharoenchai T, Chandra S, et al. Safety of Patient Mobilization and Rehabilitation in the Intensive Care Unit Systematic Review with Meta-Analysis. *Ann Am Thorac Soc* 2017;14(5):766-77. doi: 10.1513/AnnalsATS.201611-843SR
36. Kho ME, Martin RA, Toonstra AL, et al. Feasibility and safety of in-bed cycling for physical rehabilitation in the intensive care unit. *J Crit Care* 2015;30(6):1419.e1-5. doi: 10.1016/j.jcrc.2015.07.025 [published Online First: 2015/09/01]
37. Sricharoenchai T, Parker AM, Zanni JM, et al. Safety of physical therapy interventions in critically ill patients: A single-center prospective evaluation of 1110 intensive care unit admissions. *Journal of Critical Care* 2014;29(3):395-400. doi: 10.1016/j.jcrc.2013.12.012
38. Kho ME, Molloy AJ, Clarke FJ, et al. TryCYCLE: A Prospective Study of the Safety and Feasibility of Early In-Bed Cycling in Mechanically Ventilated Patients. *Plos One* 2016;11(12) doi: 10.1371/journal.pone.0167561
39. Camargo Pires-Neto R, Fogaça Kawaguchi YM, Sayuri Hirota A, et al. Very early passive cycling exercise in mechanically ventilated critically ill patients: physiological and safety aspects--a case series. *Plos One* 2013;8(9):e74182. doi: 10.1371/journal.pone.0074182 [published Online First: 2013/09/17]
40. Wilkinson OM, Bates A, Cusack R. An observational feasibility study - does early limb ergometry affect oxygen delivery and uptake in intubated critically ill patients - a comparison of two assessment methods. *BMC Anesthesiol* 2021;21(1):27. doi: 10.1186/s12871-020-01227-z [published Online First: 2021/01/27]
41. Hickmann CE, Castanares-Zapatero D, Deldicque L, et al. Impact of Very Early Physical Therapy During Septic Shock on Skeletal Muscle: A Randomized Controlled Trial. *Crit Care Med* 2018;46(9):1436-43. doi: 10.1097/ccm.0000000000003263 [published Online First: 2018/06/30]
42. Machado ADS, Pires-Neto RC, Carvalho MTX, et al. Effects that passive cycling exercise have on muscle strength, duration of mechanical ventilation, and length of hospital stay in critically ill patients: a randomized clinical trial. *J Bras Pneumol* 2017;43(2):134-39. doi: 10.1590/s1806-37562016000000170 [published Online First: 2017/05/26]
43. Takaoka A, Utgikar R, Rochwerg B, et al. The Efficacy and Safety of In-Intensive Care Unit Leg-Cycle Ergometry in Critically Ill Adults A Systematic Review and Meta-analysis. *Ann Am Thorac Soc* 2020;17(10):1289-307. doi: 10.1513/AnnalsATS.202001-059OC
44. Waldauf P, Jiroutkova K, Krajcova A, et al. Effects of Rehabilitation Interventions on Clinical Outcomes in Critically Ill Patients: Systematic Review and Meta-Analysis of Randomized Controlled Trials*. *Critical Care Medicine* 2020;48(7):1055-65. doi: 10.1097/ccm.0000000000004382
45. van Willigen Z, Collings N, Richardson D, et al. Quality improvement: The delivery of true early mobilisation in an intensive care unit. *BMJ Qual Improv Rep* 2016;5(1):u211734.w4726. doi: 10.1136/bmjquality.u211734.w4726
46. Needham DM, Korupolu R, Zanni JM, et al. Early Physical Medicine and Rehabilitation for Patients With Acute Respiratory Failure: A Quality Improvement Project. *Arch Phys Med Rehab* 2010;91(4):536-42. doi: 10.1016/j.apmr.2010.01.002
47. Morris PE, Goad A, Thompson C, et al. Early intensive care unit mobility therapy in the treatment of acute respiratory failure. *Critical Care Medicine* 2008;36(8):2238-43. doi: 10.1097/CCM.0b013e318180b90e
48. McWilliams D, Weblin J, Atkins G, et al. Enhancing rehabilitation of mechanically ventilated patients in the intensive care unit: A quality improvement project. *Journal of Critical Care* 2015;30(1):13-18. doi: 10.1016/j.jcrc.2014.09.018

For peer review only

49. Lord RK, Mayhew CR, Korupolu R, et al. ICU Early Physical Rehabilitation Programs: Financial Modeling of Cost Savings. *Critical Care Medicine* 2013;41(3):717-24. doi: 10.1097/CCM.0b013e3182711de2
50. Schujmann DS, Gomes TT, Lunardi AC, et al. Impact of a Progressive Mobility Program on the Functional Status, Respiratory, and Muscular Systems of ICU Patients: A Randomized and Controlled Trial*. *Critical Care Medicine* 2020;48(4):491-97. doi: 10.1097/ccm.0000000000004181
51. Chan A-W, Tetzlaff JM, Altman DG, et al. SPIRIT 2013 Statement: Defining Standard Protocol Items for Clinical Trials. *Annals of Internal Medicine* 2013;158(3):200-+. doi: 10.7326/0003-4819-158-3-201302050-00583
52. Hoffmann TC, Glasziou PP, Boutron I, et al. Better reporting of interventions: template for intervention description and replication (TIDieR) checklist and guide. *BMJ : British Medical Journal* 2014;348:g1687. doi: 10.1136/bmj.g1687
53. Mental Capacity Act, 2005. Available http://www.legislation.gov.uk/ukpga/**2005**/9/pdfs/ukpga_20050009_en.pdf.
54. Ely EW, Truman B, Shintani A, et al. Monitoring Sedation Status Over Time in ICU PatientsReliability and Validity of the Richmond Agitation-Sedation Scale (RASS). *JAMA* 2003;289(22):2983-91. doi: 10.1001/jama.289.22.2983
55. Sessler CN, Gosnell MS, Grap MJ, et al. The Richmond Agitation–Sedation Scale. *American Journal of Respiratory and Critical Care Medicine* 2002;166(10):1338-44. doi: 10.1164/rccm.2107138
56. Ely EW, Inouye SK, Bernard GR, et al. Delirium in mechanically ventilated patients - Validity and reliability of the Confusion Assessment Method for the intensive care unit (CAM-ICU). *Jama-J Am Med Assoc* 2001;286(21):2703-10. doi: 10.1001/jama.286.21.2703
57. De Jonghe B, Sharshar T, Lefaucheur JP, et al. Paresis acquired in the intensive care unit - A prospective multicenter study. *Jama-J Am Med Assoc* 2002;288(22):2859-67. doi: 10.1001/jama.288.22.2859
58. Denehy L, de Morton NA, Skinner EH, et al. A physical function test for use in the intensive care unit: validity, responsiveness, and predictive utility of the physical function ICU test (scored). *Phys Ther* 2013;93(12):1636-45. doi: 10.2522/ptj.20120310 [published Online First: 2013/07/28]
59. Skinner EH, Berney S, Warrillow S, et al. Development of a physical function outcome measure (PFIT) and a pilot exercise training protocol for use in intensive care. *Crit Care Resusc* 2009;11(2):110-5. [published Online First: 2009/06/03]
60. Fan E, Ciesla ND, Truong AD, et al. Inter-rater reliability of manual muscle strength testing in ICU survivors and simulated patients. *Intensive Care Med* 2010;36(6):1038-43. doi: 10.1007/s00134-010-1796-6 [published Online First: 2010/03/10]
61. Hermans G, Clerckx B, Vanhullebusch T, et al. Interobserver agreement of Medical Research Council sum-score and handgrip strength in the intensive care unit. *Muscle Nerve* 2012;45(1):18-25. doi: 10.1002/mus.22219 [published Online First: 2011/12/23]
62. Vanpee G, Segers J, Van Mechelen H, et al. The interobserver agreement of handheld dynamometry for muscle strength assessment in critically ill patients. *Crit Care Med* 2011;39(8):1929-34. doi: 10.1097/CCM.0b013e31821f050b [published Online First: 2011/05/17]
63. Corner EJ, Wood H, Englebretsen C, et al. The Chelsea critical care physical assessment tool (CPAx): validation of an innovative new tool to measure physical morbidity in the general adult critical care population; an observational proof-of-concept pilot study. *Physiotherapy* 2013;99(1):33-41. doi: 10.1016/j.physio.2012.01.003 [published Online First: 2012/12/12]
64. Hodgson C, Needham D, Haines K, et al. Feasibility and inter-rater reliability of the ICU Mobility Scale. *Heart Lung* 2014;43(1):19-24. doi: 10.1016/j.hrtlng.2013.11.003 [published Online First: 2014/01/01]

For peer review only

65. Bischoff HA, Stähelin HB, Monsch AU, et al. Identifying a cut-off point for normal mobility: a comparison of the timed 'up and go' test in community-dwelling and institutionalised elderly women. *Age Ageing* 2003;32(3):315-20. doi: 10.1093/ageing/32.3.315 [published Online First: 2003/05/02]
66. Ng SS, Hui-Chan CW. The timed up & go test: its reliability and association with lower-limb impairments and locomotor capacities in people with chronic stroke. *Arch Phys Med Rehabil* 2005;86(8):1641-7. doi: 10.1016/j.apmr.2005.01.011 [published Online First: 2005/08/09]
67. Bagshaw M, Majumdar SR, Rolfson DB, et al. A prospective multicenter cohort study of frailty in younger critically ill patients. *Crit Care* 2016;20(1):175. doi: 10.1186/s13054-016-1338-x [published Online First: 2016/06/07]
68. Bagshaw SM, Stelfox HT, Johnson JA, et al. Long-term association between frailty and health-related quality of life among survivors of critical illness: a prospective multicenter cohort study. *Crit Care Med* 2015;43(5):973-82. doi: 10.1097/ccm.0000000000000860 [published Online First: 2015/02/11]
69. Rockwood K, Song X, MacKnight C, et al. A global clinical measure of fitness and frailty in elderly people. *Cmaj* 2005;173(5):489-95. doi: 10.1503/cmaj.050051 [published Online First: 2005/09/01]
70. ATS statement: guidelines for the six-minute walk test. *Am J Respir Crit Care Med* 2002;166(1):111-7. doi: 10.1164/ajrccm.166.1.at1102 [published Online First: 2002/07/02]
71. Ustun TB, Kostanjesek N, Chatterji S, et al. Measuring health and disability : manual for WHO Disability Assessment Schedule (WHODAS 2.0) / edited by T.B. Üstün, N. Kostanjsek, S. Chatterji, J.Rehm. Geneva: World Health Organization, 2010.
72. Bjelland I, Dahl AA, Haug TT, et al. The validity of the Hospital Anxiety and Depression Scale. An updated literature review. *J Psychosom Res* 2002;52(2):69-77. doi: 10.1016/s0022-3999(01)00296-3 [published Online First: 2002/02/08]
73. Zigmond AS, Snaith RP. The hospital anxiety and depression scale. *Acta Psychiatr Scand* 1983;67(6):361-70. doi: 10.1111/j.1600-0447.1983.tb09716.x [published Online First: 1983/06/01]
74. Herdman M, Gudex C, Lloyd A, et al. Development and preliminary testing of the new five-level version of EQ-5D (EQ-5D-5L). *Qual Life Res* 2011;20(10):1727-36. doi: 10.1007/s11136-011-9903-x [published Online First: 2011/04/12]
75. Schwarzwald J, Solomon Z, Weisenberg M, et al. Validation of the Impact of Event Scale for psychological sequelae of combat. *J Consult Clin Psychol* 1987;55(2):251-6. doi: 10.1037//0022-006x.55.2.251 [published Online First: 1987/04/01]
76. Proctor E, Silmere H, Raghavan R, et al. Outcomes for Implementation Research: Conceptual Distinctions, Measurement Challenges, and Research Agenda. *Administration and Policy in Mental Health and Mental Health Services Research* 2011;38(2):65-76. doi: 10.1007/s10488-010-0319-7
77. Eldridge SM, Chan CL, Campbell MJ, et al. CONSORT 2010 statement: extension to randomised pilot and feasibility trials. *BMJ* 2016;355:i5239. doi: 10.1136/bmj.i5239
78. Sricharoenchai T, Parker AM, Zanni JM, et al. Safety of physical therapy interventions in critically ill patients: a single-center prospective evaluation of 1110 intensive care unit admissions. *J Crit Care* 2014;29(3):395-400. doi: 10.1016/j.jcrc.2013.12.012 [published Online First: 2014/02/11]
79. Kho ME, Molloy AJ, Clarke F, et al. CYCLE pilot: a protocol for a pilot randomised study of early cycle ergometry versus routine physiotherapy in mechanically ventilated patients. *Bmj Open* 2016;6(4) doi: 10.1136/bmjopen-2016-011659
80. Kayambu G, Boots RJ, Paratz JD. Early rehabilitation in sepsis: a prospective randomised controlled trial investigating functional and physiological outcomes The i-PERFORM Trial (Protocol Article). *BMC Anesthesiol* 2011;11:21. doi: 10.1186/1471-2253-11-21 [published Online First: 2011/11/01]

For peer review only

Table 2 Schedule of assessments

	Randomisation	Day 1	Day 3	Day 7	Awakening	Weekly	ICU Discharge	Hospital Discharge	3 months post hospital discharge
**Demographic Data**	X								
**Muscle assessment:**									
MRCss [60,61]					X	X	X	X	
Grip strength [62]					X	X	X	X	
**Physical function:**									
CPAX [63]		X	X	X	X	X	X		
ICU mobility [64]		X	X	X	X	X	X		
PFITs [59]					X	X	X		
Timed-Up and Go (TUG)							X	X	X
Clinical Frailty Score [69]		(X)					X	X	X
Barthel Index		(X)					X	X	X
6-minute walk test [70]								X	X

**HRQL:**	
WHODAS 2 [71]	X
HADS [72,73]	X
EQ5D-5L [74]	X
Impact of Event Scale [75]	X
Health Economic Evaluation (CSRI)*	X

Physical Function ICU Test-scored (PFITs); Medical Research Council Manual Muscle Test Sum Score (MRC-ss); Chelsea Critical Care Assessment Tool (CPAX); World Health Organisation Disability Assessment; Euroqol 5 dimension 5 level health related quality of life questionnaire; Hospital anxiety and depression scale (HADs); Client service receipt inventory(CSRI)

For peer review only

For peer review only

Table 2 Schedule of assessments

	Randomisation	Day 1	Day 3	Day 7	Awakening	Weekly	ICU Discharge	Hospital Discharge	3 months post hospital discharge
**Demographic Data**	X								
**Muscle assessment:**									
MRCss [60,61]					X	X	X	X	
Grip strength [62]					X	X	X	X	
**Physical function:**									
CPAX [63]		X	X	X	X	X	X		
ICU mobility [64]		X	X	X	X	X	X		
PFITs [59]					X	X	X		
Timed-Up and Go (TUG)							X	X	X
Clinical Frailty Score [69]		(X)					X	X	X
Barthel Index		(X)					X	X	X
6-minute walk test [70]								X	X
**HRQL:**									
WHODAS 2 [71]									X
HADS [72,73]									X
EQ5D-5L [74]									X
Impact of Event Scale [75]									X
Health Economic Evaluation (CSRI)*									X

Physical Function ICU Test-scored (PFITs); Medical Research Council Manual Muscle Test Sum Score (MRC-ss); Chelsea Critical Care Assessment Tool (CPAX); World Health Organisation Disability Assessment; Euroqol 5 dimension 5 level health related quality of life questionnaire; Hospital anxiety and depression scale (HADs); Client service receipt inventory(CSRI)

For peer review only

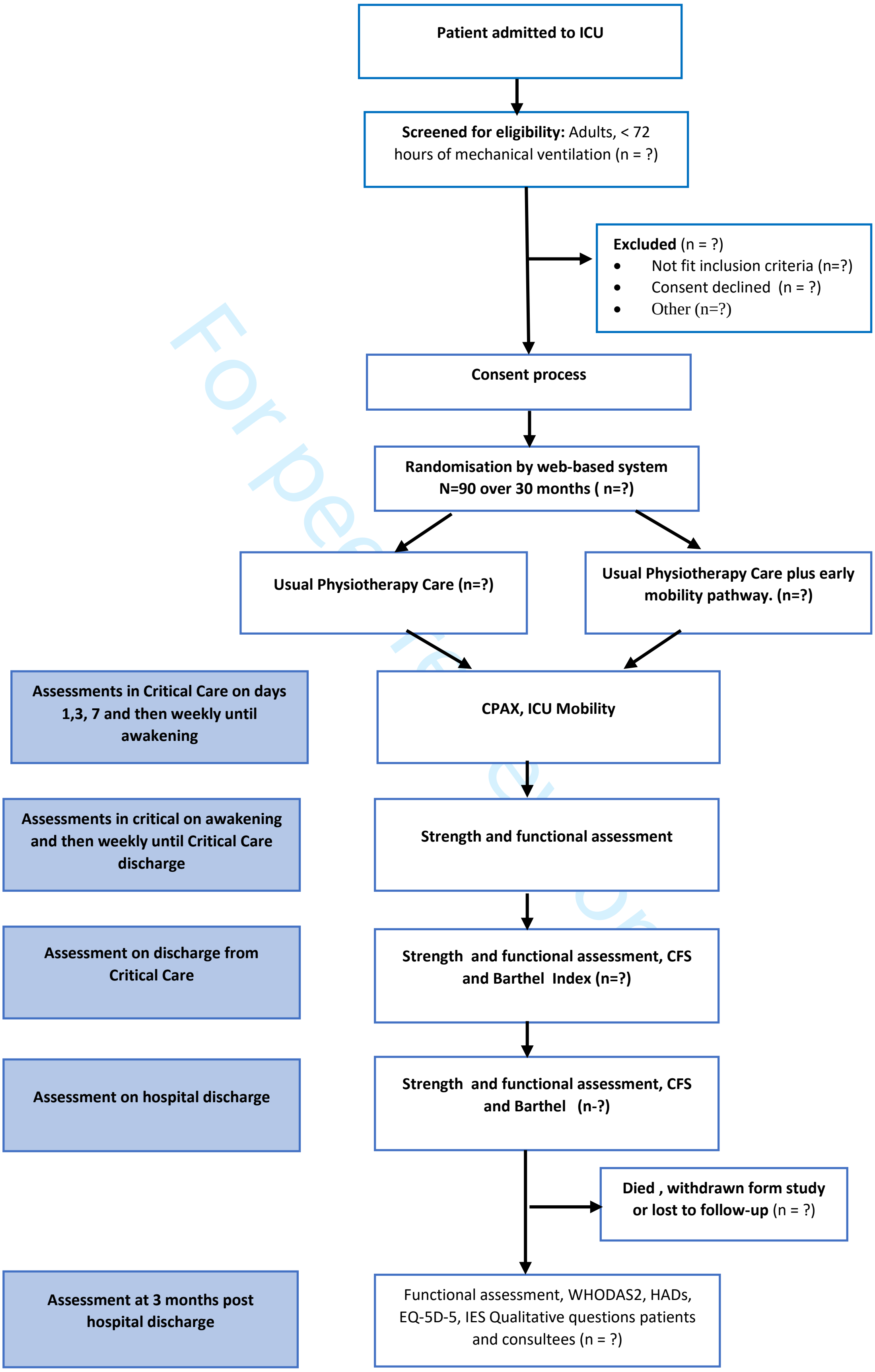

For peer review only

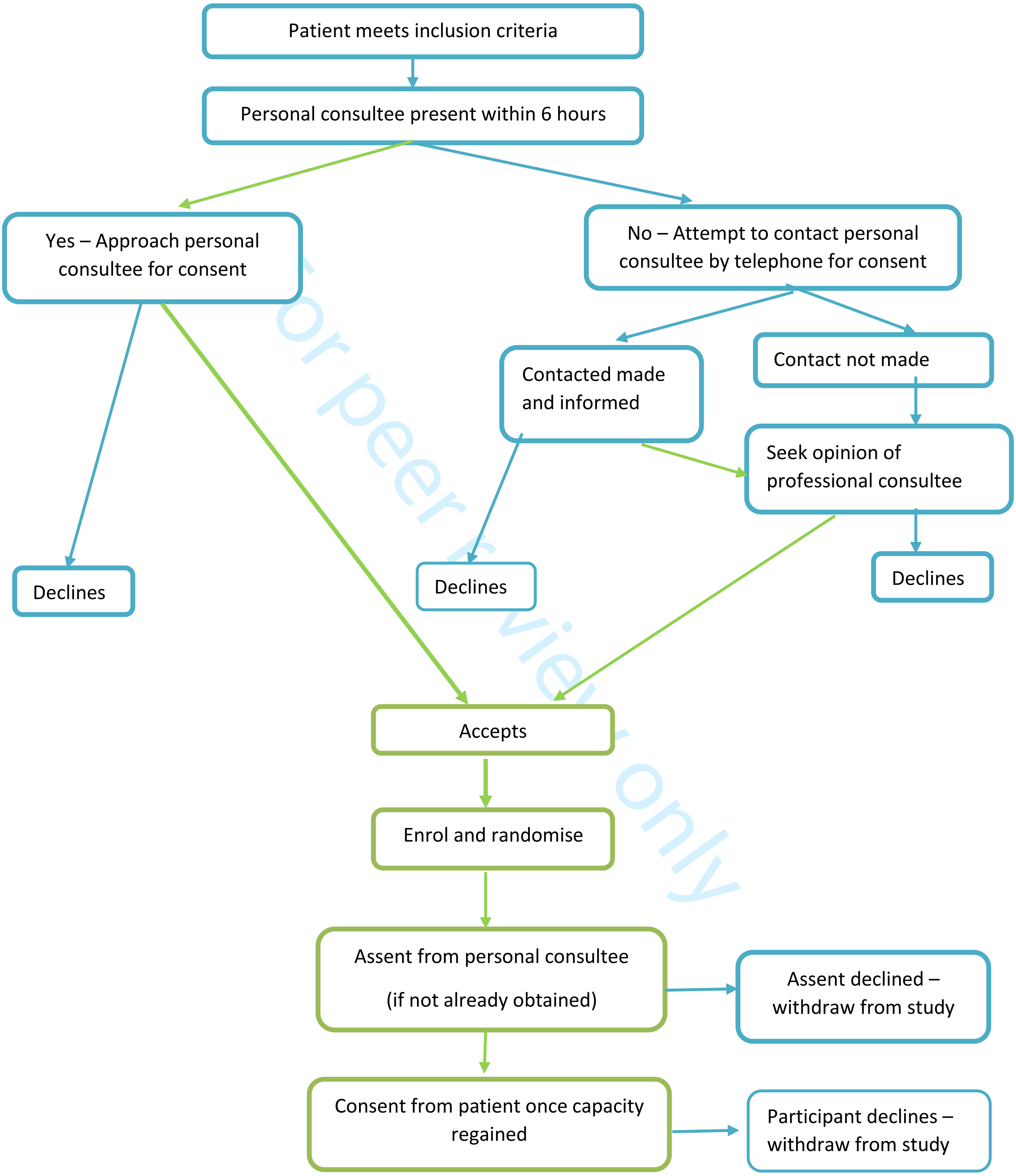
Patient meets inclusion criteria
Personal consultee present within 6 hours
Yes – Approach personal consultee for consent
No – Attempt to contact personal consultee by telephone for consent
Contacted made and informed
Contact not made
Seek opinion of professional consultee
Declines
Declines
Declines
Accepts
Enrol and randomise
Assent from personal consultee (if not already obtained)
Assent declined – withdraw from study
Consent from patient once capacity regained
Participant declines – withdraw from study

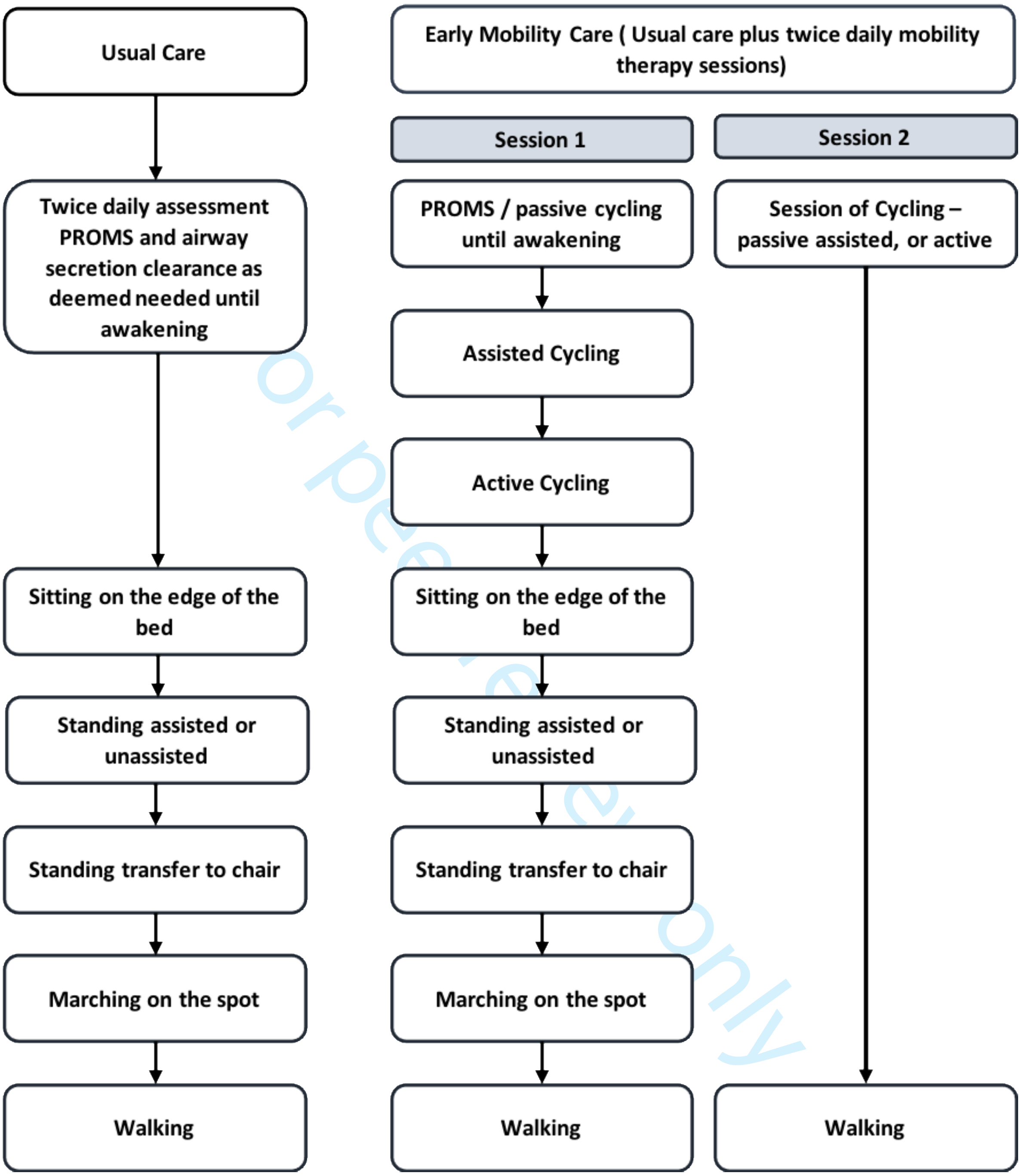
Usual Care
Twice daily assessment PROMS and airway secretion clearance as deemed needed until awakening
Sitting on the edge of the bed
Standing assisted or unassisted
Standing transfer to chair
Marching on the spot
Walking
Early Mobility Care (Usual care plus twice daily mobility therapy sessions)
Session 1
PROMS / passive cycling until awakening
Assisted Cycling
Active Cycling
Sitting on the edge of the bed
Standing assisted or unassisted
Standing transfer to chair
Marching on the spot
Walking
Session 2
Session of Cycling – passive assisted, or active
Walking

BMJ Open

Improving physical function of patients following Intensive Care Unit admission (EMPRESS): Protocol of a randomised controlled feasibility trial

Journal:	*BMJ Open*
Manuscript ID	bmjopen-2021-055285.R1
Article Type:	Protocol
Date Submitted by the Author:	16-Feb-2022

Complete List of Authors:	Cusack, Rebecca; University Hospital Southampton NHS Foundation Trust, NIHR Biomedical Research Centre ; University Hospital Southampton NHS Foundation Trust, Department of Intensive Care<br>Bates, Andrew; University Hospital Southampton NHS Foundation Trust, NIHR Southampton Biomedical Research Centre<br>Mitchell, Kay; University Hospital Southampton NHS Foundation Trust, NIHR Biomedical Research Centre<br>van Willigen, Zoe; University Hospital Southampton NHS Foundation Trust, Department of Physiotherapy<br>Denehy, Linda; The University of Melbourne Melbourne School of Health Sciences; Peter MacCallum Cancer Institute<br>Hart, Nicholas; Guy's and St Thomas' NHS Foundation Trust; King's College London, Respiratory and Critical Care<br>Dushianthan, Ahilanandan; University Hospital Southampton NHS Foundation Trust, General Intensive Care Unit; University Hospital Southampton NHS Foundation Trust, NIHR Biomedical Research Centre<br>Reading, Isabel; University of Southampton<br>Chorozoglou, Maria; University of Southampton<br>Sturmey, Gordon; University Hospital Southampton NHS Foundation Trust<br>Davey, Iain; University Hospital Southampton NHS Foundation Trust<br>Grocott, Michael; University of Southampton, Faculty of Medicine ; University Hospital Southampton NHS Foundation Trust, NIHR Biomedical Research Centre

**Primary Subject Heading**:	Intensive care
Secondary Subject Heading:	Rehabilitation medicine
Keywords:	Adult intensive & critical care < INTENSIVE & CRITICAL CARE, Quality in health care < HEALTH SERVICES ADMINISTRATION & MANAGEMENT, REHABILITATION MEDICINE

SCHOLARONE™
Manuscripts

Improving physical function of patients following Intensive Care Unit admission (EMPRESS): Protocol of a randomised controlled feasibility trial

Authors:

Rebecca J Cusack[1,2], Andrew Bates[1], Kay Mitchell[1,2], Zoe van Willigen[2], Linda Denehy[3,4], Nicholas Hart[5,6], Ahilanandan Dushianthan[1,2], Isabel Reading [1], Maria Chorozoglou [1], Gordon Sturmey[7], Iain Davey[7],Michael P W Grocott[1,2]

Author Affiliations

1 University of Southampton UK

2 University Hospital of Southampton NHS Foundation Trust UK

3 University of Melbourne Australia

4 Peter MacCallum Cancer Centre, Melbourne, Victoria, Australia

5 Guys and St Thomas' Hospital NHS Foundation Trust London UK

6 Kings College University of London UK

7 Patient advisory group

Corresponding Author:
Dr Rebecca Cusack
University Hospital of Southampton NHS FT
Tremona Road Southampton
E-mail R.Cusack@soton.ac.uk
Phone: +44 2381 202382

Word Count: 4237

Keywords: Critical care, physical therapy, rehabilitation, cycling

For peer review only

Improving physical function of patients following Intensive Care Unit admission (EMPRESS): protocol of a randomised controlled feasibility trial

Introduction: Physical rehabilitation delivered early following admission to the Intensive Care Unit (ICU) has the potential to improve short and long-term outcomes. The use of supine cycling together with other rehabilitation techniques has potential as a method of introducing rehabilitation earlier in the patient journey. The aim of the study is to determine the feasibility of delivering the designed protocol of a randomised clinical trial, comparing an protocolised early rehabilitation programme including cycling with usual care. This feasibility study will inform a larger multicentre study.

Methods and Analysis: 90 acute medical patients from 2 mixed medical-surgical ICUs will be recruited. We will include ventilated patients within 72 hours of initiation of mechanical ventilation and expected to be ventilated a further 48 hours or more. Patients will receive usual care or usual care plus two 30-minute rehabilitation sessions 5 days per week.

Feasibility outcomes are: i) recruitment 1-2 patients per month per site, ii) protocol fidelity with > 75% of patients commencing interventions within 72 hours of mechanical ventilation, > 70% interventions delivered and iii) blinded outcome measures recorded at 3 time points in > 80% of patients. Secondary outcomes are: i) strength and function; the Physical Function ICU Test-scored (PFITs) measured on ICU discharge, ii) hospital length of stay and iii) mental health and physical ability at 3 months using the WHODAS 2. An economic analysis using hospital health services data reported with an embedded health economic study will collect and assess economic and QoL data including Hospital Anxiety and Depression score (HADS), Euroqol-5 Dimension-5Level (EQ-5D-5L) and the Impact of Event Score (IES). .

Ethics and Dissemination: The study has ethical approval from South Central Hampshire A Research Ethics Committee (19/SC/0016). All amendments will be approved by this committee. An independent trial monitoring committee is overseeing the study. Results will be made available to critical care survivors, their caregivers, the critical care societies and other researchers.

Trial registration number: NCT03771014

Sponsor: University Hospital Southampton NHS Foundation Trust.

Strengths and limitations of study

- Will investigate the implementation of an protocolised early rehabilitation intervention, that is usual care in one NHS/University Teaching institution, in other NHS institutions with different organisational structures

For peer review only

- The defined cohort has been demonstrated to benefit from this type of rehabilitation in alternative health care systems
- Results will inform the design of a multi-centre randomised controlled trial (RCT
- This study is not designed to assess effectiveness of the intervention
- Inability to blind the intervention to patients, physiotherapist and clinicians involved in the delivery of the intervention.

Introduction

In 2018/19 there were over 290,000 admissions to adult ICUsin the United Kingdom (UK)[1]. Treatment advances have reduced mortality associated with critical illness[2 3], however, survival does not represent the end of the story[4]. A complex interplay between baseline health status, acute disease and the traumatic effects of intensive care treatment are associated with long-term physical, psychological and social hardship[5-10]. Patients discharged from ICU have higher mortality, higher health service costs and a reduction in employment status compared to hospitalised patients not requiring ICU [8 11].

ICU acquired weakness (ICU-AW) is characterised by rapid muscle wasting, polyneuropathy and bone demineralisation, causing pain, weakness and impaired physical function [12-14]. Contributing factors are multifactorial although immobility due to the sedation required for tolerance of ventilation plays an important role[15, 16]. Early mobilisation may mitigate these effects[17-19]. In 2009 Schweickert et al.reported that patients who received early physical therapy (within 1.5 days of mechanical ventilation) had greater functional independence at hospital discharge than patients that received usual care physical therapy[20]. A recent RCT on the impact of a progressive ICU mobility programme reported improved functional status at ICU discharge[21]. Meta-analyses and systematic reviews report that early mobilisation of ICU patients may reduce duration of mechanical ventilation and improve short term physical outcomes[22-24], however mobilisation can be difficult to implement during a patient's stay in the ICU, Moreover studies which utilised delayed rehabilitation, often more than a week after ICU admission[25-27], have not replicated these outcomes[28-34]. Barriers to early mobilisation include heavy sedation, patient's illness, lack of resources and/or clinician buy-in [35-38].In-bed cycle ergometry can provide passive activity in heavily sedated patients who are receiving vasopressors[39, 40] with minimal physiological demand [40 41] and can be transitioned to active cycling as the patient's condition improves[23, 42-44]

We implemented cycle ergometry as part of an early protocolised rehabilitation quality improvement programme with physiotherapy technicians supporting the additional workload[45]. Like other investigators, we reported reduced number of ventilator days and ICU length of stay[21 46-49].

For peer review only

The primary aim of this study is to evaluate the feasibility of a (RCT) investigating the effect of earlyprotocolised rehabilitation versus usual physiotherapy care in ICU patients. Results will inform a prospective fully powered multi-centre RCT. This protocol is reported according to SPIRIT [50] and TIDieR[51] guidelines.

Aim

The aim of this study is to determine the feasibility to deliver study procedures comparing an early protocolised mobilisation programme that includes cycling with usual care.

Objectives

Feasibility will be determined by measures of the recruitment process, intervention fidelity and outcome measurement completeness, specifically: i) Study accrual rates: a minimum of 30% of eligible patients or 1-2 patients per site per month are enrolled; ii) Protocol adherence: 75% of patients commencing intervention within 72 hours of ICU admission; minimum of 70% of planned interventions delivered and iii) Blinded outcome assessment: functional assessment performed at 3 time-points in 80% of survivors. The results will inform a larger fully powered RCT.

METHODS AND ANALYSIS

Study design:

This is a two-centre feasibility study using a two-arm RCT, randomised 1:1, with blinded outcome assessments at ICU discharge, hospital discharge and 3-month follow-up. Patients will be recruited from two general ICUs, located in the south of the UK. They will not be recruited from our ICU on account that the intervention is now standard practice at this site. Prior to each site opening to recruitment an audit of current physiotherapy practice will be undertaken over a four-week period to evaluate what constitutes 'usual care' in each institution

Participants:

Ninety patients will be recruited. Eligible patients will be over 42 years old and have an acute/unplanned medical admission to the ICU. They will be functionally independent prior to ICU admission (Barthel Index >80), in hospital for <5 days prior to intubation and ventilation, intubated and ventilated for <72 hrs and expected to remain ventilated for a further 48 hours. Patients will be excluded if in hospital for 5 days or more prior to ICU admission, have acute brain or spinal cord injury, known or suspected neurological / muscular impairment, condition limiting use of cycle ergometry (e.g. lower limb fracture / amputation), not expected to survive >48hrs decided by consulting Intensivist, persistent therapy exemptions in first 3 days of mechanical ventilation. (Figure 1) presents the planned flow of patients through the study.

For peer review only

Recruitment, consent and randomisation:

The study team will screen all patients for eligibility. Recruitment began in June 2019 (and was temporarily suspended in March 2020 due to the COVID 19 pandemic). It is anticipated recruitment will continue until early 2022. The majority of patients will have diminished capacity when first eligible, therefore, the consent process is multi-layered and designed in accordance with the Mental Capacity Act (MCA) 2005[52] (Figure 2). .*Patient Informed Consent*: Wherever possible, informed consent will be directly sought from the patient. *Personal Consultee Informed Assent*: If the patient is unable to provide consent, informed assent will be sought from the patient's personal consultee, within 6 hours of confirmation of eligibility. If the personal consultee is not available in person, attempts will be made to contact them by telephone. They will be asked to provide written assent, at the earliest possible convenience. *Professional Consultee Informed Assent:* Where both patient and personal consultee are not available to approve enrolment within 6 hours of confirmation of eligibility, assent will be sought from a professional consultee in accordance with the MCA. The professional consultee will be a consultant medical practitioner, independent from the study. The patient's personal consultee will be consulted at the earliest possible opportunity and assent requested to continue in the study.

In all cases, once the patient has regained capacity they will be informed of the study and consent continuation sought. Following consent or assent, patients will be registered on a bespoke electronic data collection tool (ALEA™) and randomly assigned to the protocolised early rehabilitation or usual care.

Staff training/ site set-up:

Participating sites will employ the equivalent of a full-time therapy technician to deliver the study intervention, under the supervision of a senior critical care therapist. Both senior critical care therapists and therapy technicians will complete a training package delivered by the primary institution (University Hospital Southampton NHS Foundation Trust), where early rehabilitation with cycling is well established and embedded in usual care. This package includes seminars on the delivery of the protocolised early rehabilitation , use of the bespoke electronic database and 5-days of clinical shadowing.

Interventions:

All patients will receive usual medical, nursing and physiotherapy care while in intensive care. Each bedside nurse will be asked at the start of the shift if they have been involved caring for a patient in the intervention arm of the study. The ICU physiotherapy team, who are not involved the study delivery of, will deliver all usual physiotherapy interventions in both groups. The physiotherapist delivering usual care will be asked to verify if they have delivered any of the study interventions. In the intervention arm the protocolised physiotherapy programme will commence within 72 hours of ICU admission or as soon as possible thereafter and continue for 28 days or until ICU discharge, whichever

For peer review only

occurs first. Patients respiratory support can range from full mandatory ventilation through to oxygen supplementation with no mechanical support following extubation. Sedation is targeted throughout the time that the patient is intubated and ventilation mode adjusted to patients' needs, compliance and comfort at discretion At the start of each physiotherapy intervention the participants level of sedation will be assessed using the Richmond Agitation-Sedation Scale (RASS) [53 54] and the Confusion Assessment Method for ICU (CAM-ICU) [55].will be undertaken. RASS will be targeted to a RASS between -1 and +1 by the bedside nurse. After 28 days of ICU admission, all patients will receive usual care physiotherapy interventions.

Group 1: Usual care control group

In this pragmatic study physiotherapy interventions will be guided by individual assessment and start in accordance with the usual care pathway within each institution. The focus of each session may be respiratory support, mobilisation or a combination of both. Interventions delivered will be determined by the physiotherapist in conjunction with the attending physician. Interventions include, where appropriate, passive or active range of movement, positioning and respiratory physiotherapy, and when able, sitting on the edge of the bed, standing (assisted or unassisted), standing to transfer to chair, marching on the spot and walking. (Figure 3). Usual interventions may occur at any time of day.

Group 2: Protocolised rehabilitation pathway

Paitients will receive usual care physiotherapy, in addition to the two protocolised intervention within 72 hours of ICU admission or as soon as possible thereafter. Patients will be screened for safety criteria to withhold the intervention prior to each planned intervention session (Table 1).

For peer review only

Table 1

	Criteria to commence physiotherapy	Criteria to stop / withhold physiotherapy intervention
Blood pressure	MAP 60 – 100 mmHg, no change in vasopressor dose requirement for preceding 2 hours	Catecholamine resistant hypotension with MAP < 60 mmHg
Heart rate	Between 40-140 bpm	<50 or >140 bpm
Respiratory rate	Sustained < 40 breaths/min	Sustained >40 breaths/min
Temperature		>40 °C
Oxygen requirement	If FiO2 >0.8 for passive exercise only<br>FiO2 <0.8 and PEEP<15 cmH_2O	
Desaturation		Sats fall <85% for > 1 minute
Other		· Fall<br>· Unplanned extubation<br>· Acute bleeding<br>· New onset arrhythmia<br>· Signs/symptoms of acute myocardial ischaemia<br>· Patient pain/distress<br>· Clinical team decide therapy intervention not appropriate<br>· Refusal by patient or representative

Those meeting criteria to withhold interventions will have issues addressed and reassessed for interventions 2 hours later. The two additional rehabilitation sessions will be delivered by the research physiotherapy staff including a therapy technician. This will comprise of two mobility sessions the modality of the first, chosen at the discretion of the physiotherapist. The second session will be 30-minutes of supine cycling. delivered in the afternoon.

The first rehabilitation intervention each day will be delivered in the morning. Planned interventions include passive or active range of movements, passive cycling, active cycling, in bed exercises, sitting, mobilisation out of bed and walking. Daily assessment of the patient will be made to ensure the highest

For peer review only - http://bmjopen.bmj.com/site/about/guidelines.xhtml

level of activity possible is provided for each individual patient given safety considerations and capability of the patient.

The second session will be cycling based. An in-bed supine cycle ergometer (MotoMed Letto 2™) will be used to engage the participant in passive, assisted or active cycling, or a combination, depending on the degree of patient co-operation (Figure3). The aim is for the patient to have 30 minutes of cycling per day, following a standardised cycling programme. If cycling is in passive mode, patients will commence cycling at 5 revolutions per minute (RPM), building up to 20 RPM over a 5minutes and continue this for 20 minutes before 5-minute 5RPM cool down. In the assisted or active mode, after the 5-minute warm up, cycling will continue for 20 minutes at patient selected RPM followed by a 5-minute cool down at 5 RPM. In-bed cycling sessions will stop when the patient is deemed to be able to stand and transfer from bed to chair for both mobility sessions for two consecutive days. If patients are considered unable to have concurrent mobility therapy and respiratory weaning, mobility therapy will take priority, in agreement with the senior clinical team. Individual participants will receive the trial intervention on five days per week (Monday to Friday) for the duration of their ICU stay or maximum of 28 days whichever comes first. Patients will be monitored for cardiovascular and respiratory stability and safety of indwelling lines, tubes and catheters with pre-determined criteria for termination of any session (Table 1). Deviations from the planned protocol will be reported to determine potential barriers to implementation. Patients will be able to decline any intervention or outcome assessment at any time without compromise to their care.

Primary Outcome: Feasibility to deliver the protocol as designed

Feasibility will be determined by measures of the recruitment process, intervention fidelity and outcome measurement completeness, specifically:

1. Study accrual rates: a minimum of 30% of eligible patients or 1-2 patients per site per month are enrolled
2. Protocol adherence: 75% of patients commencing intervention within 72 hours of ICU admission; minimum of 70% of planned interventions delivered
3. Blinded outcome assessment: functional assessment performed at 3 time-points in 80% of survivors by physiotherapists working within the hospital but not within the ICU

Secondary Outcomes:

The schedule of outcome assessments is detailed in Table 2

Strength and Function

We will measure the Physical Function ICU Test-scored (PFITs) at awakening as described by deJonghe [56] then weekly within ICU and on ICU discharge [57]. PFITs is a reliable and valid 4 item scale (arm strength, leg strength, ability to stand and step cadence), with a score range of 0-10 and is responsive to change and predictive of key outcomes [58]. Medical Research Council Manual Muscle Test

For peer review only

Sum Score (MRC-ss)[59, 60] and Handheld Dynamometry (HHD) [61] will be measured on awakening, weekly, on ICU discharge and hospital discharge. Chelsea Critical Care Physical Assessment tool (CPAX) [62] and ICU Mobility Scale [63] will be assessed three times during the first week within ICU, on awakening, weekly thereafter within the ICU and at ICU discharge. Timed Up and Go (TUG)[64, 65], Clinical Frailty Score (CFS)[66-68] and Barthel Index will be assessed at ICU discharge, hospital discharge and 3-months post-hospital discharge. Pre-admission Barthel Index and CFS will be assessed by proxy on admission from family member or next of kin. Six-minute walk test (6MWT)[69] will be performed, in accordance with American Thoracic Society guidelines, at hospital discharge and 3-months post-hospital discharge.

Health related quality of life Outcomes

The following will be measured at 3-months post-hospital discharge : WHODAS-2.0[70] , Hospital Anxiety and Depression Score (HADS)[71, 72] , Euroqol-5 Dimension-5Level (EQ-5D-5L)[73], Impact of Event Score (IES)[74] and Client Service Receipt Inventory questionnaire (CSRI), designed for this study to evaluate costs that fall on patients and their carers. Resource use and costs including direct intervention costs of therapists and equipment and general hospital costs (per bed day) will be recorded for each patient

Health economic sub-study

We will also conduct an embedded health economic study to identify and define data collection for a future RCT where a full cost effectiveness analysis (CEA) can be conducted. Within the feasibility study we aim to address the following:

- what the quality of the data and what potential problems are there for reporting QoL (EQ-5D-5L), resource use and costs;
- the cost implications of the proposed intervention in terms of impact for the NHS (inpatient stay bed days) and identifying the main cost drivers;
- is the EQ-5D-5L appropriate for use in the future RCT.

The economic outcomes will include: secondary care resource use within hospitals during inpatient stay, primary care resource use following discharge up to 3months and resource use related to providing the intervention. The results will be reported in the form of descriptive statistics and will be used to inform a future CEA within a definitive RCT.

Additional data collection

For peer review only

We will collect baseline data including demographic information, Functional Comorbidity Index, ICU diagnosis, APACHE II score, ventilation duration, ventilator free days, ICU and hospital length of stay, within ICU drug history and duration and type of usual care physiotherapy.

Implementation Evaluation

We aim to investigate whether the protocolised early rehabilitation programme used in one NHS institution is transferable, as an RCT, into other similar NHS institutions. The design of a future multi-centre study will be informed by identified facilitators and barriers to implementation. Implementation assessment will be based on the measures described by Proctor[75]. A cross section of ICU staff and patients will be interviewed and complete questionnaires at trial completion to identify barriers impacting delivery of the study . Understanding of the integration and sustainability of the intervention are necessary to inform the design of a powered RCT. Acceptability will be measured at the beginning and end of the study from investigators and clinical staff by direct discussions and questionnaire. Our experience informs us that the introduction of this intervention is dependent on a cultural change within any unit for a pro-active focus on early mobilisation. We aim to explore measures to help optimise implementation. Adoption, feasibility, and fidelity measures will be monitored during the study by regular meetings with the investigators. Patient screening logs will identify the number of patients eligible for the study and barriers to enrolment. We will assess the degree to which it is possible to separate the staff caring for the intervention group from those caring for the patients in the control group.

We will report whether trial participation has influenced usual care within the participating units by pre- and post-study audits. Participating sites will collect data regarding number and seniority of therapy staff with dedicated time to work within the ICU; delirium and sedation protocols used; time, type and frequency of rehabilitation interventions delivered, who delivers the interventions and reasons why usual care may not be delivered.

The feasibility outcomes described above will be used to power a larger RCT.

Data entry and checks

Data will be entered into the secure electronic case report form (ALEA™) and data validation will take place according to the procedures set out in the data management plan and data validation plan, both developed apriori. Missing data will be assessed to identify any specific challenges with any items of data collected. Missing data level is expected to be less than 20%. Data loss and mortality will inform number of participants needed to design a larger RCT. As this is a feasibility study data imputation will not be undertaken. Prior to statistical analysis, variables will be checked for missing

For peer review only

and impossible and improbable values as defined by clinical opinion. Questions regarding the data will be directed to the data manager.

Sample size calculation

.

This is a feasibility study the results of which will be used to power a definitive study if appropriate, as such no formal sample size calculation for effectiveness of the intervention has been undertaken. 90 patients will be recruited aiming for 30-45 participants at each site. We anticipate a 30% in hospital mortality /loss to follow-up with an estimate of 60 patients completing the study. This sample size of 90 will allow the estimate of recruitment rate to be made with a 95% confidence interval of ± 5.2% if the rate is observed to be around 30%, and with a confidence interval of ± 7.3% if the recruitment rate is observed to be around 50%. In addition, the sample of 90 recruited patients will allow the estimate of the mortality rate to be made with a 95% confidence interval of ±9.5% assuming the mortality rate was around 30%. Finally, assuming the recruitment rate was around 30%, a sample of 300 patients approached to take part in the study leading to 90 enrolled patients would allow for the recruitment rate to be estimated with a 95% confidence interval of ± 5.2%. If the recruitment rate was nearer 50%, with 180 patients approached to recruit the 90 enrolled patients, the recruitment rate would be estimated with a 95% confidence interval of ± 7.3%.

Statistical analysis

The analysis will be reported in line with the feasibility studies extension to the CONSORT statement [76]. The aims of the study are to estimate the recruitment, compliance and retention rates to inform the design of a future study and is not powered for hypothesis testing regarding the effectiveness of the intervention. Feasibility outcomes (recruitment, compliance, and retention rates) will be presented with 95% confidence intervals across the whole study population. Compliance and retention rates will also be presented by treatment arm to ensure balanced recruitment, but no formal statistical comparison tests will be made. Mortality and participant dropout rates will be presented with 95% confidence intervals across the whole study population and within treatment arm. Clinical outcome data (secondary outcomes) will be presented as summary statistics using means and standard deviations or medians and ranges/interquartile ranges, as applicable, across the whole study population and by treatment arm. These data will be used

For peer review only

to inform the future trial but will not be used to draw conclusions about the effectiveness of the protocolised early rehabilitation intervention within this study

Trial management

The Chief Investigator (CI) will ensure all study personnel are appropriately orientated and trained, oversee recruitment and report to the trial safety monitoring committee. Training will occur across sites using competency-based training developed at the primary site (University Hospital Southampton NHS Foundation Trust). A study steering group, consisting of an independent chair, expert members and 2 lay advisors will meet every 3-months. Fortnightly teleconferences with trial sites will be held to monitor conduct and progress. Timing and intervals of visits and teleconferences will be reviewed at 3 months to ensure optimal time use.

The CI and Principal Investigators will facilitate local monitoring by the Research and Development quality manager, Research Ethics committee (REC) review and provide access to source data as required. A monitoring report will be produced, summarising the visit, documents and findings. The CI will ensure that all findings are addressed appropriately. The steering group will review all events in a timely manner. Additional monitoring will be scheduled where there is evidence or suspicion of non-compliance with the study protocol.

A Data Management and Safety Committee will be chaired by an independent expert. Quarterly reports will be given to the committee once recruitment has commenced.

Patient and Public Involvement

The study has been supported by patient advisory representatives. These represen3tatives are members of the trial steering committee. Patient advisors partnered with us for the design of the study, the informational material to support the intervention, the burden of the intervention from the patient's perspective and contributed to the dissemination plan

Ethics and dissemination

Ethical approval has been granted by South Central - Hampshire A Research Ethics Committee (REC reference 19/SC/0016). EMPRESS was registered with Clinical Ttrials.gov (ref:NCT03771014) on 10th December, 2018.

Results of this proposed feasibility study will be disseminated for four key audiences: i) patients and public; ii) Intensive care staff, healthcare workers and potential future research delivery partners; iii) service delivery organisations and iv) academic and potential future research collaborators.

Dissemination activities will include: feedback to Patients and Public Involvement study focus group, feedback to study participants, presentations to local clinical teams and managers and commissioners

For peer review only

and presentation at conferences attended by appropriate healthcare professionals. Where appropriate, results will be published in peer reviewed journals.

Safety and adverse events

Early mobility within ICUs is safe. In a review of physiotherapy in a critical care rehabilitation programme, 1110 patients received 5267 rehabilitation sessions physiological abnormalities or potential adverse events occurred in only 6 per 1000 interventions [77]. Mobilisation interventions will only be delivered if patients fit the safety criteria defined in table 1. Similar safety criteria have been used in other ICU rehabilitation studies [78, 79].

All adverse events will be documented. Any intervention will cease according to stopping criteria detailed in Table 1. Any such event will be recorded as an adverse event. The CI will provide a monthly update to the safety monitoring committee. Serious adverse events are events that result in death, are life threatening or require prolonged hospitalisation. Any such event will be reported in accordance with the NHS Health Research Authority guidance.

Discussion:

EMPRESS is a feasibility study to assess if a randomised controlled trial of protocolised rehabilitation with supine cycling can be delivered in ventilated patients in ICUs with differing organisational structures with blinded follow-up assessments. A recent meta-analysis indicated that protocolised rehabilitation significantly reduces duration of mechanical ventilation and ICU length of stay[23]. This is consistent with our findings when we introduced the early rehabilitation programme outlined here in our intensive care unit[45]. Passive cycling commenced on ventilated patients may assist the recovery muscle strength in ICU patients[43] although the overall benefits of leg cycle ergometry in the critically ill is inconclusive[44]. We describe a protocolised rehabilitation programme with supine cycling delivered as close to intubation as possible, at an intensity according to the patients' highest performance capability.

Both patient and organisational issues are recognised to the delivery of early rehabilitation of the critically ill patients [35]. A frequently reported challenge is the lack of appropriately qualified staff [80]. This study evaluates the safety, feasibility, effectiveness of delivery and cost efficiency of using therapy technicians to deliver protocolised rehabilitation interventions. In addition to the clinical benefits, early physical rehabilitation can also be cost saving[49]. Even with the employment of additional therapy technicians specifically to assist in the delivery of we have found this early rehabilitation programme cost effective [81].

For peer review only

This study will collect data on the dose of intervention delivered to all patients, reasons for non-delivery of protocol interventions, and the level of experience of therapists delivering the interventions. A qualitative process evaluation is designed to identify both patient and organisational challenges that have potential to be addressed in a potential future study. Findings will inform refinement of trial design and evaluation of the intervention, clarifying causal mechanisms behind study outcomes and providing additional context not adequately captured by the quantitative data. The process evaluation will be consistent with Medical Research Council guidance for conducting process evaluations of complex healthcare interventions[82].

Targeted sedation is embedded within this protocol as oversedation is one of the more commonly cited barriers to mobilisation of the ventilated patient[35]. This study opened to recruitment prior to the publication of the recommended core outcome set for critical care ventilation trials [83] however three of the six outcomes listed (duration of mechanical ventilation, duration of stay and health related quality of life) are secondary outcomes in this study and the other 3 outcomes are included in the data collected This will be addressed should we proceed to a full RCT. Due to the nature of the intervention, it is not possible for this to be blinded however the follow-up assessments will be carried out by a blinded.

Results from EMPRESS will inform the design of a multi-centred RCT, both identifying barriers to the implementation of the designed protocol and exploring how these may be addressed from feedback from the therapy and nursing teams in addition to the feedback from patients and their next of kin.

Acknowledgements

Contributors: RJC and AB contributed equally to the preparation of the paper. RJC and ZvW had the original idea for the study, RJC, LD, IR, NH, AD, GS, ID, MPWG developed the trial protocol, IR devised the statistical analysis plan, MC developed the economic analysis. AB, GS, ID and RJC prepared and submitted documents for Research and Development and ethical approval. RJC, KM and AB wrote the manuscript. All authors reviewed the final version.

Funding: This is supported by the National Institute for Health Research (NIHR) under its Research for Patient Benefit (RfPB) Programme. Grant Reference Number PB-PG-0317-20045. KM is an NIHR Senior Nurse and Midwife Research Leader. The views expressed in this article are those of the author(s) and not necessarily those of the NIHR, or the Department of Health and Social Care

Competing Interests: None declared

For peer review only

Ethics Approval: The study has ethical approval from South Central Hampshire A Research Ethics Committee (19/SC/0016). Protocol Version 1.3 7th Feb 2019

Provenance and Peer review: Not commissioned. Protocol peer-reviewed for ethical and funding application

Disclaimer: The views expressed are those of the author(s) and not necessarily those of the NIHR, NHS or the Department of Health and Social Care.

For peer review only

Table 2

	Randomisation	Day 1	Day 3	Day 7	Awakening	Weekly	ICU Discharge	Hospital Discharge	3 months post hospital discharge
**Demographic Data**	X								
**Muscle assessment:**									
MRCss [60,61]					X	X	X	X	
Grip strength [62]					X	X	X	X	
**Physical function:**									
CPAX [63]		X	X	X	X	X	X		
ICU mobility [64]		X	X	X	X	X	X		
PFITs [59]					X	X	X		
Timed-Up and Go (TUG)							X	X	X
Clinical Frailty Score [69]		(X)					X	X	X
Barthel Index		(X)					X	X	X
6-minute walk test [70]								X	X
**HRQL:**									
WHODAS 2 [71]									X
HADS [72,73]									X
EQ5D-5L [74]									X
Impact of Event Scale [75]									X
Health Economic Evaluation (CSRI)*									X

Physical Function ICU Test-scored (PFITs); Medical Research Council Manual Muscle Test Sum Score (MRC-ss); Chelsea Critical Care Assessment Tool (CPAX); World Health Organisation Disability Assessment; Euroqol 5 dimension 5 level health related quality of life questionnaire; Hospital anxiety and depression scale (HADs); Client service receipt inventory(CSRI)

For peer review only - http://bmjopen.bmj.com/site/about/guidelines.xhtml

Legends

Table 1 Safety criteria for delivery of physical therapy interventions

Table 2 Schedule of assessments and collection of outcome data

Figure 1 Planned participants' flow

Figure 2 Study consent process

Figure 3 EMPRESS study participant rehabilitation pathway

For peer review only

References

1. Hospital Admitted Patient Care Activity 2018-19: NHS England; 2019 [National stastics]. Available at: https://digital.nhs.uk/data-and-information/publications/statistical/hospital-admitted-patient-care-activity/2018-19.[Acessed 21st Aug 2021]
2. Esteban A, Frutos-Vivar F, Muriel A, et al. Evolution of Mortality over Time in Patients Receiving Mechanical Ventilation. *American Journal of Respiratory and Critical Care Medicine* 2013;188(2):220-30. doi: 10.1164/rccm.201212-2169oc
3. Kaukonen KM, Bailey M, Suzuki S, et al. Mortality Related to Severe Sepsis and Septic Shock Among Critically Ill Patients in Australia and New Zealand, 2000-2012. *Jama-J Am Med Assoc* 2014;311(13):1308-16. doi: 10.1001/jama.2014.2637
4. Iwashyna TJ, Netzer G. The Burdens of Survivorship: An Approach to Thinking about Long-Term Outcomes after Critical Illness. *Seminars in Respiratory and Critical Care Medicine* 2012;33(4):327-38. doi: 10.1055/s-0032-1321982

5. Cheung AM, Tansey CM, Tomlinson G, et al. Two-year outcomes, health care use, and costs of survivors of acute respiratory distress syndrome. *American Journal of Respiratory and Critical Care Medicine* 2006;174(5):538-44.

6. Cuthbertson BH, Roughton S, Jenkinson D, et al. Quality of life in the five years after intensive care: a cohort study. *Critical Care* 2010;14(1) doi: 10.1186/cc8848

7. Desai SV, Law TJ, Needham DM. Long-term complications of critical care. *Critical Care Medicine* 2011;39(2):371-79.

8. Griffiths J, Hatch RA, Bishop J, et al. An exploration of social and economic outcome and associated health-related quality of life after critical illness in general intensive care unit survivors: a 12-month follow-up study. *Critical Care* 2013;17(3) doi: 10.1186/cc12745

9. Herridge MS, Tansey CM, Matte A, et al. Functional Disability 5 Years after Acute Respiratory Distress Syndrome. *New England Journal of Medicine* 2011;364(14):1293-304. doi: 10.1056/NEJMoa1011802

10. Iwashyna TJ, Ely EW, Smith DM, et al. Long-term Cognitive Impairment and Functional Disability Among Survivors of Severe Sepsis. *Jama-J Am Med Assoc* 2010;304(16):1787-94. doi: 10.1001/jama.2010.1553

11. Lone NI, Gillies MA, Haddow C, et al. Five-Year Mortality and Hospital Costs Associated with Surviving Intensive Care. *American Journal of Respiratory and Critical Care Medicine* 2016;194(2):198-208. doi: 10.1164/rccm.201511-2234OC

12. Allen C, Glasziou P, Del Mar C. Bed rest: a potentially harmful treatment needing more careful evaluation. *Lancet* 1999;354(9186):1229-33. doi: 10.1016/s0140-6736(98)10063-6

13. Jolley SE, Bunnell AE, Hough CL. ICU-Acquired Weakness. *Chest* 2016;150(5):1129-40. doi: 10.1016/j.chest.2016.03.045 [published Online First: 2016/04/07]

14. Parry SM, Puthucheary Z. The impact of extended bed rest on the musculoskeletal system in the critical care environment. *Extreme Physiology and Experimental Medicine* 2015;4

15. Puthucheary ZA, Rawal J, McPhail M, et al. Acute Skeletal Muscle Wasting in Critical Illness. *Jama-J Am Med Assoc* 2013;310(15):1591-600. doi: 10.1001/jama.2013.278481

16. Levine S, Nguyen T, Taylor N, et al. Rapid Disuse Atrophy of Diaphragm Fibers in Mechanically Ventilated Humans. *New England Journal of Medicine* 2008;358(13):1327-35. doi: 10.1056/NEJMoa070447

17. Needham DM. Mobilizing patients in the intensive care unit - Improving neuromuscular weakness and physical function. *Jama-J Am Med Assoc* 2008;300(14):1685-90. doi: 10.1001/jama.300.14.1685

18. Kress JP, Hall JB. CRITICAL CARE MEDICINE ICU-Acquired Weakness and Recovery from Critical Illness. *New England Journal of Medicine* 2014;370(17):1626-35. doi: 10.1056/NEJMra1209390

19. Hodgson CL, Berney S, Harrold M, et al. Clinical review: early patient mobilization in the ICU. *Crit Care* 2013;17(1):207. doi: 10.1186/cc11820 [published Online First: 2013/05/16]

20. Schweickert WD, Pohlman MC, Pohlman AS, et al. Early physical and occupational therapy in mechanically ventilated, critically ill patients: a randomised controlled trial. *Lancet* 2009;373(9678):1874-82. doi: 10.1016/s0140-6736(09)60658-9

21. Schujmann DS, Gomes TT, Lunardi AC, et al. Impact of a Progressive Mobility Program on the Functional Status, Respiratory, and Muscular Systems of ICU Patients: A Randomized and Controlled Trial*. *Critical Care Medicine* 2020;48(4):491-97. doi: 10.1097/ccm.0000000000004181

22. Calvo-Ayala E, Khan BA, Farber MO, et al. Interventions to Improve the Physical Function of ICU Survivors A Systematic Review. *Chest* 2013;144(5):1469-80. doi: 10.1378/chest.13-0779

23. Waldauf P, Jiroutkova K, Krajcova A, et al. Effects of Rehabilitation Interventions on Clinical Outcomes in Critically Ill Patients: Systematic Review and Meta-Analysis of Randomized Controlled Trials*. *Critical Care Medicine* 2020;48(7):1055-65. doi: 10.1097/ccm.0000000000004382

For peer review only

24. Kayambu G, Boots R, Paratz J. Physical Therapy for the Critically Ill in the ICU: A Systematic Review and Meta-Analysis. *Critical Care Medicine* 2013;41(6):1543-54. doi: 10.1097/CCM.0b013e31827ca637
25. Berney SC, Harrold M, Webb SA, et al. Intensive care unit mobility practices in Australia and New Zealand: a point prevalence study. *Critical Care and Resuscitation* 2013;15(4):260-65.
26. Harrold ME, Salisbury LG, Webb SA, et al. Early mobilisation in intensive care units in Australia and Scotland: a prospective, observational cohort study examining mobilisation practises and barriers. *Critical Care* 2015;19 doi: 10.1186/s13054-015-1033-3
27. Nydahl P, Ruhl AP, Bartoszek G, et al. Early Mobilization of Mechanically Ventilated Patients: A 1-Day Point-Prevalence Study in Germany*. *Critical Care Medicine* 2014;42(5):1178-86. doi: 10.1097/ccm.0000000000000149
28. McWilliams D, Jones C, Atkins G, et al. Earlier and enhanced rehabilitation of mechanically ventilated patients in critical care: A feasibility randomised controlled trial. *J Crit Care* 2018;44:407-12. doi: 10.1016/j.jcrc.2018.01.001 [published Online First: 2018/01/15]
29. Wright SE, Thomas K, Watson G, et al. Intensive versus standard physical rehabilitation therapy in the critically ill (EPICC): a multicentre, parallel-group, randomised controlled trial. *Thorax* 2018;73(3):213-21. doi: 10.1136/thoraxjnl-2016-209858
30. Denehy L, Skinner EH, Edbrooke L, et al. Exercise rehabilitation for patients with critical illness: a randomized controlled trial with 12 months of follow-up. *Critical Care* 2013;17(4) doi: 10.1186/cc12835
31. Moss M, Nordon-Craft A, Malone D, et al. A Randomized Trial of an Intensive Physical Therapy Program for Patients with Acute Respiratory Failure. *Am J Respir Crit Care Med* 2016;193(10):1101-10. doi: 10.1164/rccm.201505-1039OC [published Online First: 2015/12/15]
32. Walsh TS, Salisbury LG, Boyd J, et al. A randomised controlled trial evaluating a rehabilitation complex intervention for patients following intensive care discharge: the RECOVER study. *Bmj Open* 2012;2(4) doi: ARTN e001475 10.1136/bmjopen-2012-001475
33. Cuthbertson BH, Rattray J, Campbell MK, et al. The PRaCTICaL study of nurse led, intensive care follow-up programmes for improving long term outcomes from critical illness: a pragmatic randomised controlled trial. *BMJ* 2009;339(oct16 1):b3723-b23. doi: 10.1136/bmj.b3723
34. Elliott D, McKinley S, Alison J, et al. Health-related quality of life and physical recovery after a critical illness: a multi-centre randomised controlled trial of a home-based physical rehabilitation program. *Critical Care* 2011;15(3) doi: 10.1186/cc10265
35. Dubb R, Nydahl P, Hermes C, et al. Barriers and Strategies for Early Mobilization of Patients in Intensive Care Units. *Ann Am Thorac Soc* 2016;13(5):724-30. doi: 10.1513/AnnalsATS.201509-586CME
36. Nydahl P, Sricharoenchai T, Chandra S, et al. Safety of Patient Mobilization and Rehabilitation in the Intensive Care Unit Systematic Review with Meta-Analysis. *Ann Am Thorac Soc* 2017;14(5):766-77. doi: 10.1513/AnnalsATS.201611-843SR
37. Kho ME, Martin RA, Toonstra AL, et al. Feasibility and safety of in-bed cycling for physical rehabilitation in the intensive care unit. *J Crit Care* 2015;30(6):1419.e1-5. doi: 10.1016/j.jcrc.2015.07.025 [published Online First: 2015/09/01]
38. Sricharoenchai T, Parker AM, Zanni JM, et al. Safety of physical therapy interventions in critically ill patients: A single-center prospective evaluation of 1110 intensive care unit admissions. *Journal of Critical Care* 2014;29(3):395-400. doi: 10.1016/j.jcrc.2013.12.012
39. Kho ME, Molloy AJ, Clarke FJ, et al. TryCYCLE: A Prospective Study of the Safety and Feasibility of Early In-Bed Cycling in Mechanically Ventilated Patients. *Plos One* 2016;11(12) doi: 10.1371/journal.pone.0167561
40. Camargo Pires-Neto R, Fogaça Kawaguchi YM, Sayuri Hirota A, et al. Very early passive cycling exercise in mechanically ventilated critically ill patients: physiological and safety aspects--a

For peer review only

case series. *Plos One* 2013;8(9):e74182. doi: 10.1371/journal.pone.0074182 [published Online First: 2013/09/17]

41. Wilkinson OM, Bates A, Cusack R. An observational feasibility study - does early limb ergometry affect oxygen delivery and uptake in intubated critically ill patients - a comparison of two assessment methods. *BMC Anesthesiol* 2021;21(1):27. doi: 10.1186/s12871-020-01227-z [published Online First: 2021/01/27]
42. Hickmann CE, Castanares-Zapatero D, Deldicque L, et al. Impact of Very Early Physical Therapy During Septic Shock on Skeletal Muscle: A Randomized Controlled Trial. *Crit Care Med* 2018;46(9):1436-43. doi: 10.1097/ccm.0000000000003263 [published Online First: 2018/06/30]
43. Machado ADS, Pires-Neto RC, Carvalho MTX, et al. Effects that passive cycling exercise have on muscle strength, duration of mechanical ventilation, and length of hospital stay in critically ill patients: a randomized clinical trial. *J Bras Pneumol* 2017;43(2):134-39. doi: 10.1590/s1806-37562016000000170 [published Online First: 2017/05/26]
44. Takaoka A, Utgikar R, Rochwerg B, et al. The Efficacy and Safety of In-Intensive Care Unit Leg-Cycle Ergometry in Critically Ill Adults A Systematic Review and Meta-analysis. *Ann Am Thorac Soc* 2020;17(10):1289-307. doi: 10.1513/AnnalsATS.202001-059OC
45. van Willigen Z, Collings N, Richardson D, et al. Quality improvement: The delivery of true early mobilisation in an intensive care unit. *BMJ Qual Improv Rep* 2016;5(1):u211734.w4726. doi: 10.1136/bmjquality.u211734.w4726
46. Needham DM, Korupolu R, Zanni JM, et al. Early Physical Medicine and Rehabilitation for Patients With Acute Respiratory Failure: A Quality Improvement Project. *Arch Phys Med Rehab* 2010;91(4):536-42. doi: 10.1016/j.apmr.2010.01.002
47. Morris PE, Goad A, Thompson C, et al. Early intensive care unit mobility therapy in the treatment of acute respiratory failure. *Critical Care Medicine* 2008;36(8):2238-43. doi: 10.1097/CCM.0b013e318180b90e
48. McWilliams D, Weblin J, Atkins G, et al. Enhancing rehabilitation of mechanically ventilated patients in the intensive care unit: A quality improvement project. *Journal of Critical Care* 2015;30(1):13-18. doi: 10.1016/j.jcrc.2014.09.018
49. Lord RK, Mayhew CR, Korupolu R, et al. ICU Early Physical Rehabilitation Programs: Financial Modeling of Cost Savings. *Critical Care Medicine* 2013;41(3):717-24. doi: 10.1097/CCM.0b013e3182711de2
50. Chan A-W, Tetzlaff JM, Altman DG, et al. SPIRIT 2013 Statement: Defining Standard Protocol Items for Clinical Trials. *Annals of Internal Medicine* 2013;158(3):200-+. doi: 10.7326/0003-4819-158-3-201302050-00583
51. Hoffmann TC, Glasziou PP, Boutron I, et al. Better reporting of interventions: template for intervention description and replication (TIDieR) checklist and guide. *BMJ : British Medical Journal* 2014;348:g1687. doi: 10.1136/bmj.g1687
52. Mental Capacity Act, 2005.Availble at 2005. Legislation.gov.uk, Available at: http://www.legislation.gov.uk/ukpga/2005/9/contents [Accessed 7 Dec 2021].
53. Ely EW, Truman B, Shintani A, et al. Monitoring Sedation Status Over Time in ICU PatientsReliability and Validity of the Richmond Agitation-Sedation Scale (RASS). *JAMA* 2003;289(22):2983-91. doi: 10.1001/jama.289.22.2983
54. Sessler CN, Gosnell MS, Grap MJ, et al. The Richmond Agitation–Sedation Scale. *American Journal of Respiratory and Critical Care Medicine* 2002;166(10):1338-44. doi: 10.1164/rccm.2107138
55. Ely EW, Inouye SK, Bernard GR, et al. Delirium in mechanically ventilated patients - Validity and reliability of the Confusion Assessment Method for the intensive care unit (CAM-ICU). *Jama-J Am Med Assoc* 2001;286(21):2703-10. doi: 10.1001/jama.286.21.2703
56. De Jonghe B, Sharshar T, Lefaucheur JP, et al. Paresis acquired in the intensive care unit - A prospective multicenter study. *Jama-J Am Med Assoc* 2002;288(22):2859-67. doi: 10.1001/jama.288.22.2859

For peer review only

57. Denehy L, de Morton NA, Skinner EH, et al. A physical function test for use in the intensive care unit: validity, responsiveness, and predictive utility of the physical function ICU test (scored). *Phys Ther* 2013;93(12):1636-45. doi: 10.2522/ptj.20120310 [published Online First: 2013/07/28]
58. Skinner EH, Berney S, Warrillow S, et al. Development of a physical function outcome measure (PFIT) and a pilot exercise training protocol for use in intensive care. *Crit Care Resusc* 2009;11(2):110-5. [published Online First: 2009/06/03]
59. Fan E, Ciesla ND, Truong AD, et al. Inter-rater reliability of manual muscle strength testing in ICU survivors and simulated patients. *Intensive Care Med* 2010;36(6):1038-43. doi: 10.1007/s00134-010-1796-6 [published Online First: 2010/03/10]
60. Hermans G, Clerckx B, Vanhullebusch T, et al. Interobserver agreement of Medical Research Council sum-score and handgrip strength in the intensive care unit. *Muscle Nerve* 2012;45(1):18-25. doi: 10.1002/mus.22219 [published Online First: 2011/12/23]
61. Vanpee G, Segers J, Van Mechelen H, et al. The interobserver agreement of handheld dynamometry for muscle strength assessment in critically ill patients. *Crit Care Med* 2011;39(8):1929-34. doi: 10.1097/CCM.0b013e31821f050b [published Online First: 2011/05/17]
62. Corner EJ, Wood H, Englebretsen C, et al. The Chelsea critical care physical assessment tool (CPAx): validation of an innovative new tool to measure physical morbidity in the general adult critical care population; an observational proof-of-concept pilot study. *Physiotherapy* 2013;99(1):33-41. doi: 10.1016/j.physio.2012.01.003 [published Online First: 2012/12/12]
63. Hodgson C, Needham D, Haines K, et al. Feasibility and inter-rater reliability of the ICU Mobility Scale. *Heart Lung* 2014;43(1):19-24. doi: 10.1016/j.hrtlng.2013.11.003 [published Online First: 2014/01/01]
64. Bischoff HA, Stähelin HB, Monsch AU, et al. Identifying a cut-off point for normal mobility: a comparison of the timed 'up and go' test in community-dwelling and institutionalised elderly women. *Age Ageing* 2003;32(3):315-20. doi: 10.1093/ageing/32.3.315 [published Online First: 2003/05/02]
65. Ng SS, Hui-Chan CW. The timed up & go test: its reliability and association with lower-limb impairments and locomotor capacities in people with chronic stroke. *Arch Phys Med Rehabil* 2005;86(8):1641-7. doi: 10.1016/j.apmr.2005.01.011 [published Online First: 2005/08/09]
66. Bagshaw M, Majumdar SR, Rolfson DB, et al. A prospective multicenter cohort study of frailty in younger critically ill patients. *Crit Care* 2016;20(1):175. doi: 10.1186/s13054-016-1338-x [published Online First: 2016/06/07]
67. Bagshaw SM, Stelfox HT, Johnson JA, et al. Long-term association between frailty and health-related quality of life among survivors of critical illness: a prospective multicenter cohort study. *Crit Care Med* 2015;43(5):973-82. doi: 10.1097/ccm.0000000000000860 [published Online First: 2015/02/11]
68. Rockwood K, Song X, MacKnight C, et al. A global clinical measure of fitness and frailty in elderly people. *Cmaj* 2005;173(5):489-95. doi: 10.1503/cmaj.050051 [published Online First: 2005/09/01]
69. ATS statement: guidelines for the six-minute walk test. *Am J Respir Crit Care Med* 2002;166(1):111-7. doi: 10.1164/ajrccm.166.1.at1102 [published Online First: 2002/07/02]
70. Ustun TB, Kostanjesek N, Chatterji S, et al. Measuring health and disability : manual for WHO Disability Assessment Schedule (WHODAS 2.0) / edited by T.B. Üstün, N. Kostanjsek, S. Chatterji, J.Rehm. Geneva: World Health Organization, 2010.
71. Bjelland I, Dahl AA, Haug TT, et al. The validity of the Hospital Anxiety and Depression Scale. An updated literature review. *J Psychosom Res* 2002;52(2):69-77. doi: 10.1016/s0022-3999(01)00296-3 [published Online First: 2002/02/08]

For peer review only

72. Zigmond AS, Snaith RP. The hospital anxiety and depression scale. *Acta Psychiatr Scand* 1983;67(6):361-70. doi: 10.1111/j.1600-0447.1983.tb09716.x [published Online First: 1983/06/01]
73. Herdman M, Gudex C, Lloyd A, et al. Development and preliminary testing of the new five-level version of EQ-5D (EQ-5D-5L). *Qual Life Res* 2011;20(10):1727-36. doi: 10.1007/s11136-011-9903-x [published Online First: 2011/04/12]
74. Schwarzwald J, Solomon Z, Weisenberg M, et al. Validation of the Impact of Event Scale for psychological sequelae of combat. *J Consult Clin Psychol* 1987;55(2):251-6. doi: 10.1037//0022-006x.55.2.251 [published Online First: 1987/04/01]
75. Proctor E, Silmere H, Raghavan R, et al. Outcomes for Implementation Research: Conceptual Distinctions, Measurement Challenges, and Research Agenda. *Administration and Policy in Mental Health and Mental Health Services Research* 2011;38(2):65-76. doi: 10.1007/s10488-010-0319-7
76. Eldridge SM, Chan CL, Campbell MJ, et al. CONSORT 2010 statement: extension to randomised pilot and feasibility trials. *BMJ* 2016;355:i5239. doi: 10.1136/bmj.i5239
77. Sricharoenchai T, Parker AM, Zanni JM, et al. Safety of physical therapy interventions in critically ill patients: a single-center prospective evaluation of 1110 intensive care unit admissions. *J Crit Care* 2014;29(3):395-400. doi: 10.1016/j.jcrc.2013.12.012 [published Online First: 2014/02/11]
78. Kho ME, Molloy AJ, Clarke F, et al. CYCLE pilot: a protocol for a pilot randomised study of early cycle ergometry versus routine physiotherapy in mechanically ventilated patients. *Bmj Open* 2016;6(4) doi: 10.1136/bmjopen-2016-011659
79. Kayambu G, Boots RJ, Paratz JD. Early rehabilitation in sepsis: a prospective randomised controlled trial investigating functional and physiological outcomes The i-PERFORM Trial (Protocol Article). *BMC Anesthesiol* 2011;11:21. doi: 10.1186/1471-2253-11-21 [published Online First: 2011/11/01]
80. Parry SM, Knight LD, Connolly B, et al. Factors influencing physical activity and rehabilitation in survivors of critical illness: a systematic review of quantitative and qualitative studies. *Intensive Care Med* 2017;43(4):531-42. doi: 10.1007/s00134-017-4685-4 [published Online First: 2017/02/18]
81. van Willigen Z, Gallagher L, Cusack R, et al. NEW ROLES ON ICU: THE COST EFFECTIVENESS OF EMPLOYING THERAPY TECHNICIANS ON INTENSIVE CARE TO REDUCE LENGTH OF STAY AND IMPROVE FUNCTIONAL OUTCOMES. *Intens Care Med* 2013;39:S488-S88.
82. Skivington K, Matthews L, Simpson SA, et al. A new framework for developing and evaluating complex interventions: update of Medical Research Council guidance. *BMJ* 2021;374:n2061. doi: 10.1136/bmj.n2061
83. Blackwood B, Ringrow S, Clarke M, et al. A Core Outcome Set for Critical Care Ventilation Trials. *Critical care medicine* 2019;47(10):1324-31. doi: 10.1097/CCM.0000000000003904

For peer review only

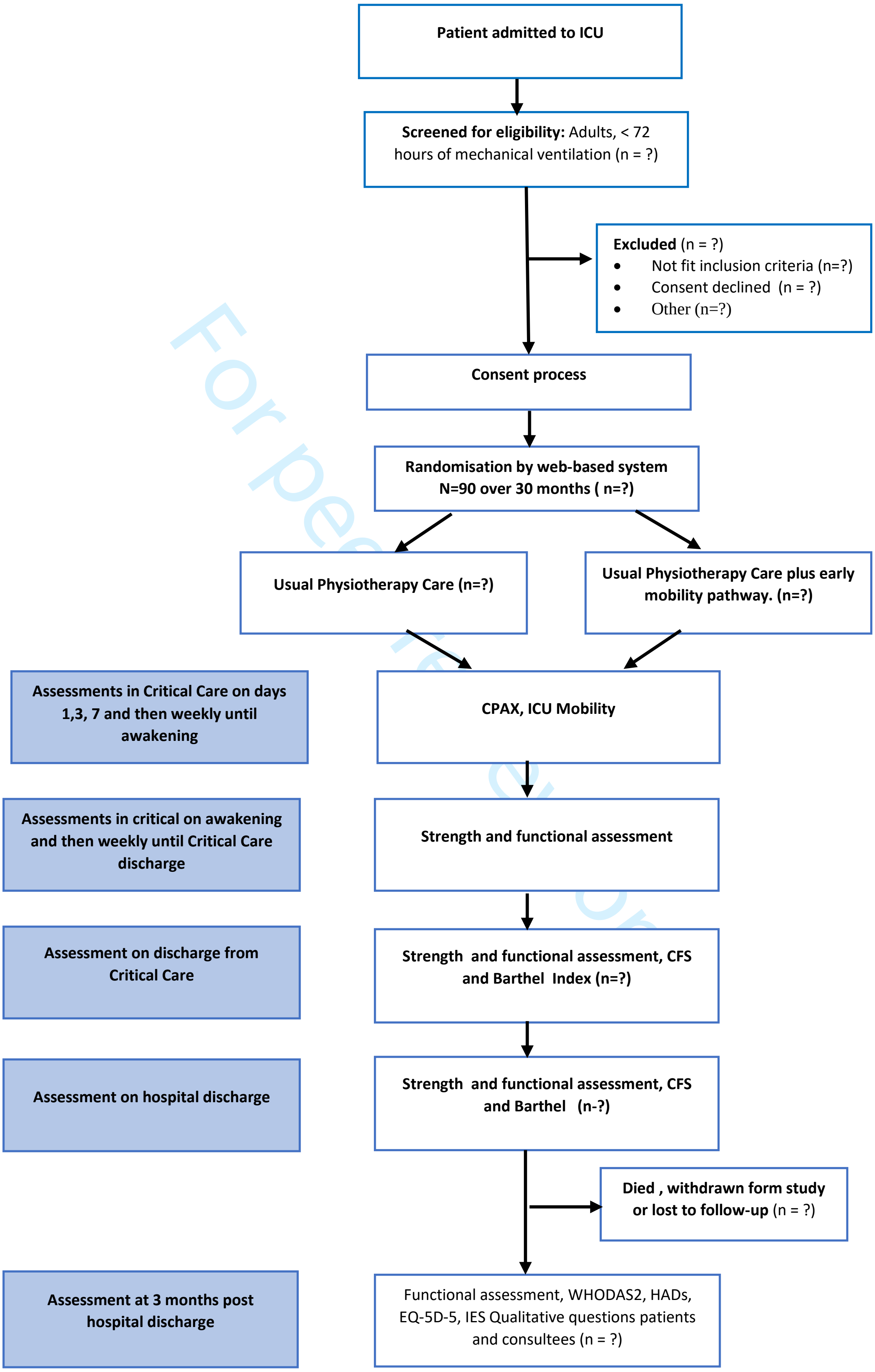
Patient admitted to ICU
Screened for eligibility: Adults, < 72 hours of mechanical ventilation (n = ?)
Excluded (n = ?)
Not fit inclusion criteria (n=?)
Consent declined (n = ?)
Other (n=?)
Consent process
Randomisation by web-based system N=90 over 30 months (n=?)
Usual Physiotherapy Care (n=?)
Usual Physiotherapy Care plus early mobility pathway. (n=?)
Assessments in Critical Care on days 1,3, 7 and then weekly until awakening
CPAX, ICU Mobility
Assessments in critical on awakening and then weekly until Critical Care discharge
Strength and functional assessment
Assessment on discharge from Critical Care
Strength and functional assessment, CFS and Barthel Index (n=?)
Assessment on hospital discharge
Strength and functional assessment, CFS and Barthel (n-?)
Died , withdrawn form study or lost to follow-up (n = ?)
Assessment at 3 months post hospital discharge
Functional assessment, WHODAS2, HADs, EQ-5D-5, IES Qualitative questions patients and consultees (n = ?)

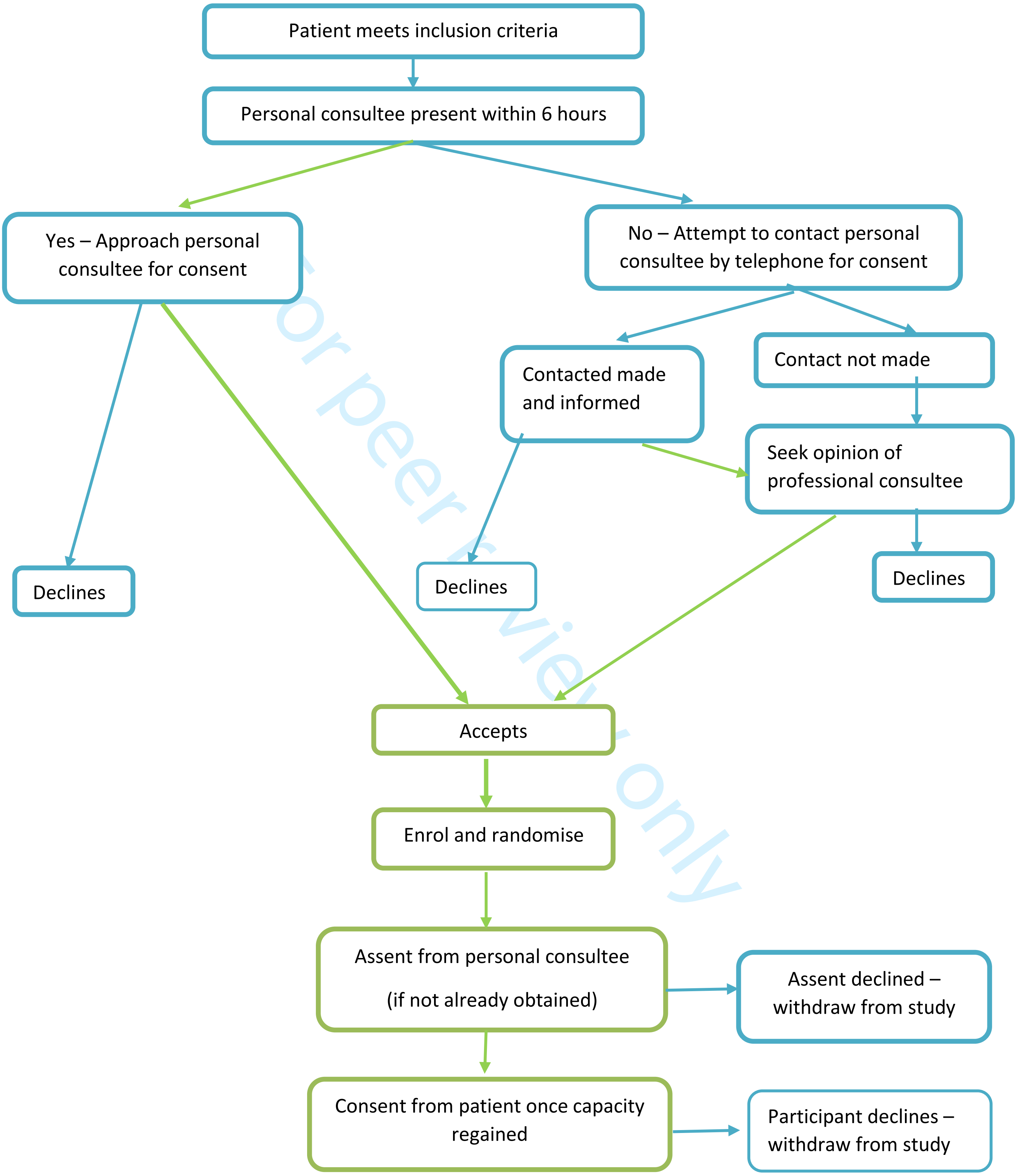
Patient meets inclusion criteria
Personal consultee present within 6 hours
Yes – Approach personal consultee for consent
No – Attempt to contact personal consultee by telephone for consent
Contacted made and informed
Contact not made
Seek opinion of professional consultee
Declines
Declines
Declines
Accepts
Enrol and randomise
Assent from personal consultee
(if not already obtained)
Assent declined – withdraw from study
Consent from patient once capacity regained
Participant declines – withdraw from study

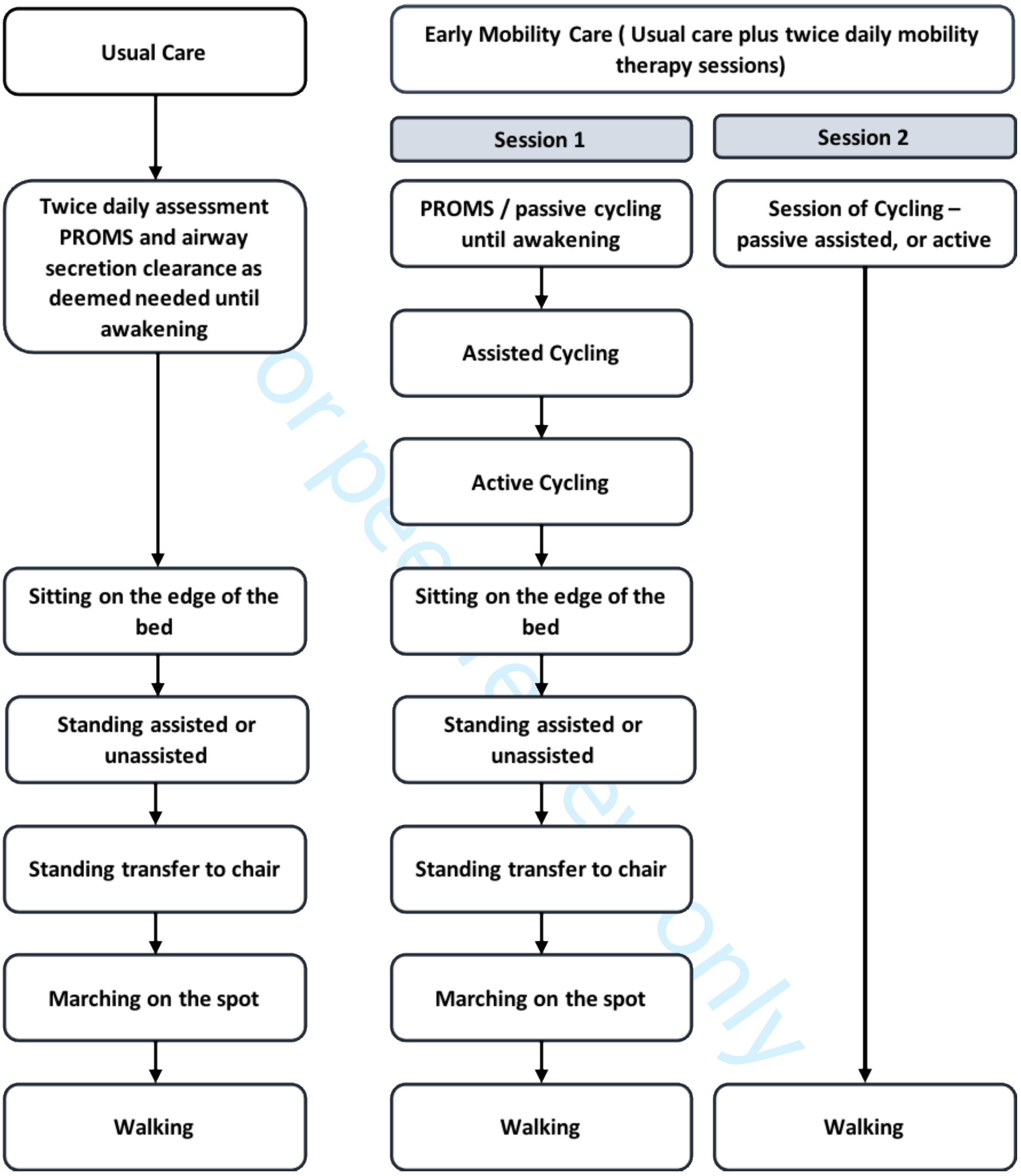

For peer review only - http://bmjopen.bmj.com/site/about/guidelines.xhtml

STANDARD PROTOCOL ITEMS: RECOMMENDATIONS FOR INTERVENTIONAL TRIALS

SPIRIT 2013 Checklist: Recommended items to address in a clinical trial protocol and related documents*

Section/item	Item No	Description
**Administrative information**		
Title	1	Descriptive title identifying the study design, population, interventions, and, if applicable, trial acronym Pg1 lines 1-2
Trial registration	2	Trial identifier and registry name. If not yet registered, name of intended registry Pg2 line 28
Protocol version	3	Date and version identifier Pg 14 line 410
Funding	4	Sources and types of financial, material, and other support Pg 14 lines 403-4
Roles and responsibilities	5a	Names, affiliations, and roles of protocol contributors Title page
	5b	Name and contact information for the trial sponsor Pg2 line 29
	5c	Role of study sponsor and funders, if any, in study design; collection, management, analysis, and interpretation of data; writing of the report; and the decision to submit the report for publication, including whether they will have ultimate authority over any of these activities N/A
	5d	Composition, roles, and responsibilities of the coordinating centre, steering committee, endpoint adjudication committee, data management team, and other individuals or groups overseeing the trial, if applicable (see Item 21a for data monitoring committee) Trial management group/ Data safety group/PPI group -- Pg12 Lines 315 to Pg 13 line 334
**Introduction**		
Background and rationale	6a	Description of research question and justification for undertaking the trial, including summary of relevant studies (published and unpublished) examining benefits and harms for each intervention See introduction Pg3 line 44-Pg line 80
	6b	Explanation for choice of comparators See introduction Pg3 line 44-Pg line 80
Objectives	7	Specific objectives or hypotheses Pg 3 lines 82-88

For peer review only - http://bmjopen.bmj.com/site/about/guidelines.xhtml

Trial design	8	Description of trial design including type of trial (eg, parallel group, crossover, factorial, single group), allocation ratio, and framework (eg, superiority, equivalence, noninferiority, exploratory) Study design Pg 4 lines 91-97

Methods: Participants, interventions, and outcomes

Study setting	9	Description of study settings (eg, community clinic, academic hospital) and list of countries where data will be collected. Reference to where list of study sites can be obtained Pg 4 Lines 93-95
Eligibility criteria	10	Inclusion and exclusion criteria for participants. If applicable, eligibility criteria for study centres and individuals who will perform the interventions (eg, surgeons, psychotherapists) Pg4 Lines 99 -108
Interventions	11a	Interventions for each group with sufficient detail to allow replication, including how and when they will be administered Pg 5- 8 lines 137 - 198
	11b	Criteria for discontinuing or modifying allocated interventions for a given trial participant (eg, drug dose change in response to harms, participant request, or improving/worsening disease) Table 1
	11c	Strategies to improve adherence to intervention protocols, and any procedures for monitoring adherence (eg, drug tablet return, laboratory tests)N/A
	11d	Relevant concomitant care and interventions that are permitted or prohibited during the trial N/A
Outcomes	12	Primary, secondary, and other outcomes, including the specific measurement variable (eg, systolic blood pressure), analysis metric (eg, change from baseline, final value, time to event), method of aggregation (eg, median, proportion), and time point for each outcome. Explanation of the clinical relevance of chosen efficacy and harm outcomes is strongly recommended Primary outcomes Pg8 lines 200-208; Secondary outcomes lines 210-244
Participant timeline	13	Time schedule of enrolment, interventions (including any run-ins and washouts), assessments, and visits for participants. A schematic diagram is highly recommended (see Figure) Table 2
Sample size	14	Estimated number of participants needed to achieve study objectives and how it was determined, including clinical and statistical assumptions supporting any sample size calculations Pg 10 line 284-300
Recruitment	15	Strategies for achieving adequate participant enrolment to reach target sample sizePg5 Lines 110-127

Methods: Assignment of interventions (for controlled trials)

Allocation:

Sequence generation	16a	Method of generating the allocation sequence (eg, computer-generated random numbers), and list of any factors for stratification. To reduce predictability of a random sequence, details of any planned restriction (eg, blocking) should be provided in a separate document that is unavailable to those who enrol participants or assign interventions Pg10 Line 126-7
Allocation concealment mechanism	16b	Mechanism of implementing the allocation sequence (eg, central telephone; sequentially numbered, opaque, sealed envelopes), describing any steps to conceal the sequence until interventions are assigned Pg10 line 126-7
Implementation	16c	Who will generate the allocation sequence, who will enrol participants, and who will assign participants to interventions Pg10 line 126-7
Blinding (masking)	17a	Who will be blinded after assignment to interventions (eg, trial participants, care providers, outcome assessors, data analysts), and how Pg14 line 390
	17b	If blinded, circumstances under which unblinding is permissible, and procedure for revealing a participant's allocated intervention during the trial N/A

Methods: Data collection, management, and analysis

Data collection methods	18a	Plans for assessment and collection of outcome, baseline, and other trial data, including any related processes to promote data quality (eg, duplicate measurements, training of assessors) and a description of study instruments (eg, questionnaires, laboratory tests) along with their reliability and validity, if known. Reference to where data collection forms can be found, if not in the protocol Lines 278 - 286
	18b	Plans to promote participant retention and complete follow-up, including list of any outcome data to be collected for participants who discontinue or deviate from intervention protocols Lines 306-7
Data management	19	Plans for data entry, coding, security, and storage, including any related processes to promote data quality (eg, double data entry; range checks for data values). Reference to where details of data management procedures can be found, if not in the protocol Lines 279-86
Statistical methods	20a	Statistical methods for analysing primary and secondary outcomes. Reference to where other details of the statistical analysis plan can be found, if not in the protocol Lines 305- 317
	20b	Methods for any additional analyses (eg, subgroup and adjusted analyses) N/A

	20c	Definition of analysis population relating to protocol non-adherence (eg, as randomised analysis), and any statistical methods to handle missing data (eg, multiple imputation) Line 84
**Methods: Monitoring**		
Data monitoring	21a	Composition of data monitoring committee (DMC); summary of its role and reporting structure; statement of whether it is independent from the sponsor and competing interests; and reference to where further details about its charter can be found, if not in the protocol. Alternatively, an explanation of why a DMC is not needed Lines 332-333
	21b	Description of any interim analyses and stopping guidelines, including who will have access to these interim results and make the final decision to terminate the trial N/A
Harms	22	Plans for collecting, assessing, reporting, and managing solicited and spontaneously reported adverse events and other unintended effects of trial interventions or trial conduct Lines 351-361
Auditing	23	Frequency and procedures for auditing trial conduct, if any, and whether the process will be independent from investigators and the sponsor N/A
**Ethics and dissemination**		
Research ethics approval	24	Plans for seeking research ethics committee/institutional review board (REC/IRB) approval Line 24-27
Protocol amendments	25	Plans for communicating important protocol modifications (eg, changes to eligibility criteria, outcomes, analyses) to relevant parties (eg, investigators, REC/IRBs, trial participants, trial registries, journals, regulators) .Line 25
Consent or assent	26a	Who will obtain informed consent or assent from potential trial participants or authorised surrogates, and how (see Item 32)Lines 109-122
	26b	Additional consent provisions for collection and use of participant data and biological specimens in ancillary studies, if applicable N/A
Confidentiality	27	How personal information about potential and enrolled participants will be collected, shared, and maintained in order to protect confidentiality before, during, and after the trial Line 279
Declaration of interests	28	Financial and other competing interests for principal investigators for the overall trial and each study site Line 413
Access to data	29	Statement of who will have access to the final trial dataset, and disclosure of contractual agreements that limit such access for investigators Line 326-7 and 332-333

review only

Ancillary and post-trial care	30	Provisions, if any, for ancillary and post-trial care, and for compensation to those who suffer harm from trial participation Lines 360-361
Dissemination policy	31a	Plans for investigators and sponsor to communicate trial results to participants, healthcare professionals, the public, and other relevant groups (eg, via publication, reporting in results databases, or other data sharing arrangements), including any publication restrictions Lines 343-345
	31b	Authorship eligibility guidelines and any intended use of professional writersLines 402-406
	31c	Plans, if any, for granting public access to the full protocol, participant-level dataset, and statistical code Lines 348-
**Appendices**		
Informed consent materials	32	Model consent form and other related documentation given to participants and authorised surrogates Can be supplied if requored
Biological specimens	33	Plans for collection, laboratory evaluation, and storage of biological specimens for genetic or molecular analysis in the current trial and for future use in ancillary studies, if applicable N/A

*It is strongly recommended that this checklist be read in conjunction with the SPIRIT 2013 Explanation & Elaboration for important clarification on the items. Amendments to the protocol should be tracked and dated. The SPIRIT checklist is copyrighted by the SPIRIT Group under the Creative Commons "Attribution-NonCommercial-NoDerivs 3.0 Unported" license.

For peer review only

BMJ Open

Improving physical function of patients following Intensive Care Unit admission (EMPRESS): Protocol of a randomised controlled feasibility trial

Journal:	*BMJ Open*
Manuscript ID	bmjopen-2021-055285.R2
Article Type:	Protocol
Date Submitted by the Author:	15-Mar-2022
Complete List of Authors:	Cusack, Rebecca; University Hospital Southampton NHS Foundation Trust, NIHR Biomedical Research Centre ; University Hospital Southampton NHS Foundation Trust, Department of Intensive Care<br>Bates, Andrew; University Hospital Southampton NHS Foundation Trust, NIHR Southampton Biomedical Research Centre<br>Mitchell, Kay; University Hospital Southampton NHS Foundation Trust, NIHR Biomedical Research Centre<br>van Willigen, Zoe; University Hospital Southampton NHS Foundation Trust, Department of Physiotherapy<br>Denehy, Linda; The University of Melbourne Melbourne School of Health Sciences; Peter MacCallum Cancer Institute<br>Hart, Nicholas; Guy's and St Thomas' NHS Foundation Trust; King's College London, Respiratory and Critical Care<br>Dushianthan, Ahilanandan; University Hospital Southampton NHS Foundation Trust, General Intensive Care Unit; University Hospital Southampton NHS Foundation Trust, NIHR Biomedical Research Centre<br>Reading, Isabel; University of Southampton<br>Chorozoglou, Maria; University of Southampton<br>Sturmey, Gordon; University Hospital Southampton NHS Foundation Trust<br>Davey, Iain; University Hospital Southampton NHS Foundation Trust<br>Grocott, Michael; University of Southampton, Faculty of Medicine ; University Hospital Southampton NHS Foundation Trust, NIHR Biomedical Research Centre
**Primary Subject Heading**:	Intensive care
Secondary Subject Heading:	Rehabilitation medicine
Keywords:	Adult intensive & critical care < INTENSIVE & CRITICAL CARE, Quality in health care < HEALTH SERVICES ADMINISTRATION & MANAGEMENT, REHABILITATION MEDICINE

SCHOLARONE™
Manuscripts

Improving physical function of patients following Intensive Care Unit admission (EMPRESS): Protocol of a randomised controlled feasibility trial

Authors:

Rebecca J Cusack[1,2], Andrew Bates[1], Kay Mitchell[1,2], Zoe van Willigen[2], Linda Denehy[3,4], Nicholas Hart[5,6], Ahilanandan Dushianthan[1,2], Isabel Reading [1], Maria Chorozoglou [1], Gordon Sturmey[7], Iain Davey[7],Michael P W Grocott[1,2]

Author Affiliations

1 University of Southampton UK

2 University Hospital of Southampton NHS Foundation Trust UK

3 University of Melbourne Australia

4 Peter MacCallum Cancer Centre, Melbourne, Victoria, Australia

5 Guys and St Thomas' Hospital NHS Foundation Trust London UK

6 Kings College University of London UK

7 Patient advisory group

Corresponding Author:
Dr Rebecca Cusack
University Hospital of Southampton NHS FT
Tremona Road Southampton
E-mail R.Cusack@soton.ac.uk
Phone: +44 2381 202382

Word Count: 4237

Keywords: Critical care, physical therapy, rehabilitation, cycling

For peer review only

Improving physical function of patients following Intensive Care Unit admission (EMPRESS): protocol of a randomised controlled feasibility trial

Introduction: Physical rehabilitation delivered early following admission to the Intensive Care Unit (ICU) has the potential to improve short and long-term outcomes. The use of supine cycling together with other rehabilitation techniques has potential as a method of introducing rehabilitation earlier in the patient journey. The aim of the study is to determine the feasibility of delivering the designed protocol of a randomised clinical trial, comparing a protocolised early rehabilitation programme including cycling with usual care. This feasibility study will inform a larger multicentre study.

Methods and Analysis: 90 acute medical patients from 2 mixed medical-surgical ICUs will be recruited. We will include ventilated patients within 72 hours of initiation of mechanical ventilation and expected to be ventilated a further 48 hours or more. Patients will receive usual care or usual care plus two 30-minute rehabilitation sessions 5 days per week.

Feasibility outcomes are: i) recruitment 1-2 patients per month per site, ii) protocol fidelity with > 75% of patients commencing interventions within 72 hours of mechanical ventilation, > 70% interventions delivered and iii) blinded outcome measures recorded at 3 time points in > 80% of patients. Secondary outcomes are: i) strength and function; the Physical Function ICU Test-scored (PFITs) measured on ICU discharge, ii) hospital length of stay and iii) mental health and physical ability at 3 months using the WHODAS 2. An economic analysis using hospital health services data reported with an embedded health economic study will collect and assess economic and QoL data including Hospital Anxiety and Depression score (HADS), Euroqol-5 Dimension-5 Level (EQ-5D-5L) and the Impact of Event Score (IES).

Ethics and Dissemination: The study has ethical approval from South Central Hampshire A Research Ethics Committee (19/SC/0016). All amendments will be approved by this committee. An independent trial monitoring committee is overseeing the study. Results will be made available to critical care survivors, their caregivers, the critical care societies and other researchers.

Trial registration number: NCT03771014

Sponsor: University Hospital Southampton NHS Foundation Trust.

Strengths and limitations of study

- Will investigate the implementation of a protocolised early rehabilitation intervention, that is usual care in one NHS/University Teaching institution, in other NHS institutions with different organisational structures

For peer review only

- The defined cohort has been demonstrated to benefit from this type of rehabilitation in alternative health care systems
- Results will inform the design of a multi-centre randomised controlled trial (RCT
- This study is not designed to assess effectiveness of the intervention
- Inability to blind the intervention to patients, physiotherapist and clinicians involved in the delivery of the intervention.

Introduction

In 2018/19 there were over 290,000 admissions to adult ICUs in the United Kingdom (UK)[1]. Treatment advances have reduced mortality associated with critical illness[2, 3], however, survival does not represent the end of the story[4]. A complex interplay between baseline health status, acute disease and the traumatic effects of intensive care treatment are associated with long-term physical, psychological and social hardship[5-10]. Patients discharged from ICU have higher mortality, higher health service costs and a reduction in employment status compared to hospitalised patients not requiring ICU[8,11].

ICU acquired weakness (ICU-AW) is characterised by rapid muscle wasting, polyneuropathy and bone demineralisation, causing pain, weakness and impaired physical function [12-14]. Contributing factors are multifactorial although immobility due to the sedation required for tolerance of ventilation plays an important role[15,16]. Early mobilisation may mitigate these effects[17-19]. In 2009 Schweickert et al.reported that patients who received early physical therapy (within 1.5 days of mechanical ventilation) had greater functional independence at hospital discharge than patients that received usual care physical therapy[20]. A recent RCT on the impact of a progressive ICU mobility programme reported improved functional status at ICU discharge[21]. Meta-analyses and systematic reviews report that early mobilisation of ICU patients may reduce duration of mechanical ventilation and improve short term physical outcomes[22-24], however mobilisation can be difficult to implement during a patient's stay in the ICU, Moreover studies which utilised delayed rehabilitation, often more than a week after ICU admission[25-27], have not replicated these outcomes[28-34]. Barriers to early mobilisation include heavy sedation, patient's illness, lack of resources and/or clinician buy-in[35-38].In-bed cycle ergometry can provide passive activity in heavily sedated patients who are receiving vasopressors[39, 40] with minimal physiological demand[40,41] and can be transitioned to active cycling as the patient's condition improves[23, 42-44]

We implemented cycle ergometry as part of an early protocolised rehabilitation quality improvement programme with physiotherapy technicians supporting the additional workload[45]. Like other investigators, we reported reduced number of ventilator days and ICU length of stay[21, 46-49].

For peer review only

The primary aim of this study is to evaluate the feasibility of a (RCT) investigating the effect of early protocolised rehabilitation versus usual physiotherapy care in ICU patients. Results will inform a prospective fully powered multi-centre RCT. This protocol is reported according to SPIRIT [50] and TIDieR[51] guidelines.

Aim

The aim of this study is to determine the feasibility to deliver study procedures comparing an early protocolised mobilisation programme that includes cycling with usual care.

Objectives

Feasibility will be determined by measures of the recruitment process, intervention fidelity and outcome measurement completeness, specifically: i) Study accrual rates: a minimum of 30% of eligible patients or 1-2 patients per site per month are enrolled; ii) Protocol adherence: 75% of patients commencing intervention within 72 hours of ICU admission; minimum of 70% of planned interventions delivered and iii) Blinded outcome assessment: functional assessment performed at 3 time-points in 80% of survivors. The results will inform a larger fully powered RCT.

METHODS AND ANALYSIS

Study design:

This is a two-centre feasibility study using a two-arm RCT, randomised 1:1, with blinded outcome assessments at ICU discharge, hospital discharge and 3-month follow-up. Patients will be recruited from two general ICUs, located in the south of the UK. They will not be recruited from our ICU on account that the intervention is now standard practice at this site. Prior to each site opening to recruitment an audit of current physiotherapy practice will be undertaken over a four-week period to evaluate what constitutes 'usual care' in each institution.

Participants:

Ninety patients will be recruited. Eligible patients will be over 42 years old and have an acute/unplanned medical admission to the ICU. They will be functionally independent prior to ICU admission (Barthel Index >80), in hospital for <5 days prior to intubation and ventilation, intubated and ventilated for <72 hrs and expected to remain ventilated for a further 48 hours. Patients will be excluded if in hospital for 5 days or more prior to ICU admission, have acute brain or spinal cord injury, known or suspected neurological / muscular impairment, condition limiting use of cycle ergometry (e.g. lower limb fracture /amputation), not expected to survive >48hrs decided by consulting Intensivist, persistent therapy exemptions in first 3 days of mechanical ventilation. (Figure 1) presents the planned flow of patients through the study.

For peer review only

Recruitment, consent and randomisation:

The study team will screen all patients for eligibility. Recruitment began in June 2019 (and was temporarily suspended in March 2020 due to the COVID 19 pandemic). It is anticipated recruitment will continue until early 2022. The majority of patients will have diminished capacity when first eligible, therefore, the consent process is multi-layered and designed in accordance with the Mental Capacity Act (MCA) 2005[52] (Figure 2). *Patient Informed Consent*: Wherever possible, informed consent will be directly sought from the patient (see supplementary files 1 and 2). *Personal Consultee Informed Assent*: If the patient is unable to provide consent, informed assent will be sought from the patient's personal consultee, within 6 hours of confirmation of eligibility. If the personal consultee is not available in person, attempts will be made to contact them by telephone. They will be asked to provide written assent, at the earliest possible convenience (see supplementary files 3 and 4). *Professional Consultee Informed Assent:* Where both patient and personal consultee are not available to approve enrolment within 6 hours of confirmation of eligibility, assent will be sought from a professional consultee in accordance with the MCA. The professional consultee will be a consultant medical practitioner, independent from the study. The patient's personal consultee will be consulted at the earliest possible opportunity and assent requested to continue in the study.

In all cases, once the patient has regained capacity they will be informed of the study and consent continuation sought. Following consent or assent, patients will be registered on a bespoke electronic data collection tool (ALEA^{TM}) and randomly assigned to the protocolised early rehabilitation or usual care.

Staff training/ site set-up:

Participating sites will employ the equivalent of a full-time therapy technician to deliver the study intervention, under the supervision of a senior critical care therapist. Both senior critical care therapists and therapy technicians will complete a training package delivered by the primary institution (University Hospital Southampton NHS Foundation Trust), where early rehabilitation with cycling is well established and embedded in usual care. This package includes seminars on the delivery of the protocolised early rehabilitation, use of the bespoke electronic database and 5-days of clinical shadowing.

Interventions:

All patients will receive usual medical, nursing and physiotherapy care while in intensive care. Each bedside nurse will be asked at the start of the shift if they have been involved caring for a patient in the intervention arm of the study. The ICU physiotherapy team, who are not involved the study delivery of, will deliver all usual physiotherapy interventions in both groups. The physiotherapist delivering usual care will be asked to verify if they have delivered any of the study interventions. In the intervention arm the protocolised physiotherapy programme will commence within 72 hours of ICU

For peer review only

admission or as soon as possible thereafter and continue for 28 days or until ICU discharge, whichever occurs first. Patients respiratory support can range from full mandatory ventilation through to oxygen supplementation with no mechanical support following extubation. Sedation is targeted throughout the time that the patient is intubated and ventilation mode adjusted to patients' needs, compliance and comfort at discretion At the start of each physiotherapy intervention the participants level of sedation will be assessed using the Richmond Agitation-Sedation Scale (RASS)[53,54] and the Confusion Assessment Method for ICU (CAM-ICU)[55].will be undertaken. RASS will be targeted to a RASS between -1 and +1 by the bedside nurse. After 28 days of ICU admission, all patients will receive usual care physiotherapy interventions.

Group 1: Usual care control group

In this pragmatic study physiotherapy interventions will be guided by individual assessment and start in accordance with the usual care pathway within each institution. The focus of each session may be respiratory support, mobilisation or a combination of both. Interventions delivered will be determined by the physiotherapist in conjunction with the attending physician. Interventions include, where appropriate, passive or active range of movement, positioning and respiratory physiotherapy, and when able, sitting on the edge of the bed, standing (assisted or unassisted), standing to transfer to chair, marching on the spot and walking. (Figure 3). Usual interventions may occur at any time of day.

Group 2: Protocolised rehabilitation pathway

Patients will receive usual care physiotherapy, in addition to the two protocolised intervention within 72 hours of ICU admission or as soon as possible thereafter. Patients will be screened for safety criteria to withhold the intervention prior to each planned intervention session (Table 1).

For peer review only

Table 1 Safety criteria for delivery of physical therapy interventions

	**Criteria to commence physiotherapy**	**Criteria to stop / withhold physiotherapy intervention**
Blood pressure	MAP 60 – 100 mmHg, no change in vasopressor dose requirement for preceding 2 hours	Catecholamine resistant hypotension with MAP < 60 mmHg
Heart rate	Between 40-140 bpm	<50 or >140 bpm
Respiratory rate	Sustained < 40 breaths/min	Sustained >40 breaths/min
Temperature		>40 °C
Oxygen requirement	If FiO2 >0.8 for passive exercise only<br>FiO2 <0.8 and PEEP<15 cmH_2O	
Desaturation		Sats fall <85% for > 1 minute
Other		· Fall<br>· Unplanned extubation<br>· Acute bleeding<br>· New onset arrhythmia<br>· Signs/symptoms of acute myocardial ischaemia<br>· Patient pain/distress<br>· Clinical team decide therapy intervention not appropriate<br>· Refusal by patient or representative

Those meeting criteria to withhold interventions will have issues addressed and reassessed for interventions 2 hours later. The two additional rehabilitation sessions will be delivered by the research physiotherapy staff including a therapy technician. This will comprise of two mobility sessions the modality of the first, chosen at the discretion of the physiotherapist. The second session will be 30-minutes of supine cycling. delivered in the afternoon.

The first rehabilitation intervention each day will be delivered in the morning. Planned interventions include passive or active range of movements, passive cycling, active cycling, in bed exercises, sitting, mobilisation out of bed and walking. Daily assessment of the patient will be made to ensure the highest

level of activity possible is provided for each individual patient given safety considerations and capability of the patient.

The second session will be cycling based. An in-bed supine cycle ergometer (MotoMed Letto 2^{TM}) will be used to engage the participant in passive, assisted or active cycling, or a combination, depending on the degree of patient co-operation (Figure3). The aim is for the patient to have 30 minutes of cycling per day, following a standardised cycling programme. If cycling is in passive mode, patients will commence cycling at 5 revolutions per minute (RPM), building up to 20 RPM over 5 minutes and continue this for 20 minutes before 5-minute 5 RPM cool down. In the assisted or active mode, after the 5-minute warm up, cycling will continue for 20 minutes at patient selected RPM followed by a 5-minute cool down at 5 RPM. In-bed cycling sessions will stop when the patient is deemed to be able to stand and transfer from bed to chair for both mobility sessions for two consecutive days. If patients are considered unable to have concurrent mobility therapy and respiratory weaning, mobility therapy will take priority, in agreement with the senior clinical team. Individual participants will receive the trial intervention on five days per week (Monday to Friday) for the duration of their ICU stay or maximum of 28 days whichever comes first. Patients will be monitored for cardiovascular and respiratory stability and safety of indwelling lines, tubes and catheters with pre-determined criteria for termination of any session (Table 1). Deviations from the planned protocol will be reported to determine potential barriers to implementation. Patients will be able to decline any intervention or outcome assessment at any time without compromise to their care.

Primary Outcome: Feasibility to deliver the protocol as designed

Feasibility will be determined by measures of the recruitment process, intervention fidelity and outcome measurement completeness, specifically:

1. Study accrual rates: a minimum of 30% of eligible patients or 1-2 patients per site per month are enrolled
2. Protocol adherence: 75% of patients commencing intervention within 72 hours of ICU admission; minimum of 70% of planned interventions delivered
3. Blinded outcome assessment: functional assessment performed at 3 time-points in 80% of survivors by physiotherapists working within the hospital but not within the ICU

Secondary Outcomes:

The schedule of outcome assessments is detailed in Table 2

Strength and Function

We will measure the Physical Function ICU Test-scored (PFITs) at awakening as described by deJonghe [56] then weekly within ICU and on ICU discharge [57]. PFITs is a reliable and valid 4 item scale (arm strength, leg strength, ability to stand and step cadence), with a score range of 0-10 and is responsive to change and predictive of key outcomes [58]. Medical Research Council Manual Muscle Test

For peer review only

219 Sum Score (MRC-ss)[59, 60] and Handheld Dynamometry (HHD)[61] will be measured on awakening, weekly, on ICU discharge and hospital discharge. Chelsea Critical Care Physical Assessment tool (CPAX) [62] and ICU Mobility Scale [63] will be assessed three times during the first week within ICU, on awakening, weekly thereafter within the ICU and at ICU discharge. Timed Up and Go (TUG)[64, 65], Clinical Frailty Score (CFS)[66-68] and Barthel Index will be assessed at ICU discharge, hospital discharge and 3-months post-hospital discharge. Pre-admission Barthel Index and CFS will be assessed by proxy on admission from family member or next of kin. Six-minute walk test (6MWT)[69] will be performed, in accordance with American Thoracic Society guidelines, at hospital discharge and 3-months post-hospital discharge.

Health related quality of life Outcomes

The following will be measured at 3-months post-hospital discharge : WHODAS-2.0[70] , Hospital Anxiety and Depression Score (HADS)[71, 72], Euroqol-5 Dimension-5Level (EQ-5D-5L)[73], Impact of Event Score (IES)[74] and Client Service Receipt Inventory questionnaire (CSRI), designed for this study to evaluate costs that fall on patients and their carers. Resource use and costs including direct intervention costs of therapists and equipment and general hospital costs (per bed day) will be recorded for each patient

Health economic sub-study

We will also conduct an embedded health economic study to identify and define data collection for a future RCT where a full cost effectiveness analysis (CEA) can be conducted. Within the feasibility study we aim to address the following:

- what the quality of the data and what potential problems are there for reporting QoL (EQ-5D-5L), resource use and costs.
- the cost implications of the proposed intervention in terms of impact for the NHS (inpatient stay bed days) and identifying the main cost drivers.
- is the EQ-5D-5L appropriate for use in the future RCT.

The economic outcomes will include: secondary care resource use within hospitals during inpatient stay, primary care resource use following discharge up to 3months and resource use related to providing the intervention. The results will be reported in the form of descriptive statistics and will be used to inform a future CEA within a definitive RCT.

Additional data collection

For peer review only

We will collect baseline data including demographic information, Functional Comorbidity Index, ICU diagnosis, APACHE II score, ventilation duration, ventilator free days, ICU and hospital length of stay, within ICU drug history and duration and type of usual care physiotherapy.

Implementation Evaluation

We aim to investigate whether the protocolised early rehabilitation programme used in one NHS institution is transferable, as an RCT, into other similar NHS institutions. The design of a future multi-centre study will be informed by identified facilitators and barriers to implementation. Implementation assessment will be based on the measures described by Proctor[75]. A cross section of ICU staff and patients will be interviewed and complete questionnaires at trial completion to identify barriers impacting delivery of the study. Understanding of the integration and sustainability of the intervention are necessary to inform the design of a powered RCT. Acceptability will be measured at the beginning and end of the study from investigators and clinical staff by direct discussions and questionnaire. Our experience informs us that the introduction of this intervention is dependent on a cultural change within any unit for a pro-active focus on early mobilisation. We aim to explore measures to help optimise implementation. Adoption, feasibility, and fidelity measures will be monitored during the study by regular meetings with the investigators. Patient screening logs will identify the number of patients eligible for the study and barriers to enrolment. We will assess the degree to which it is possible to separate the staff caring for the intervention group from those caring for the patients in the control group.

We will report whether trial participation has influenced usual care within the participating units by pre- and post-study audits. Participating sites will collect data regarding number and seniority of therapy staff with dedicated time to work within the ICU; delirium and sedation protocols used; time, type and frequency of rehabilitation interventions delivered, who delivers the interventions and reasons why usual care may not be delivered.

The feasibility outcomes described above will be used to power a larger RCT.

Data entry and checks

Data will be entered into the secure electronic case report form (ALEA^{TM}) and data validation will take place according to the procedures set out in the data management plan and data validation plan, both developed apriori. Missing data will be assessed to identify any specific challenges with any items of data collected. Missing data level is expected to be less than 20%. Data loss and mortality will inform number of participants needed to design a larger RCT. As this is a feasibility study data imputation will not be undertaken. Prior to statistical analysis, variables will be checked for missing

For peer review only

and impossible and improbable values as defined by clinical opinion. Questions regarding the data will be directed to the data manager.

Sample size calculation

.

This is a feasibility study the results of which will be used to power a definitive study if appropriate, as such no formal sample size calculation for effectiveness of the intervention has been undertaken. 90 patients will be recruited aiming for 30-45 participants at each site. We anticipate a 30% in hospital mortality /loss to follow-up with an estimate of 60 patients completing the study. This sample size of 90 will allow the estimate of recruitment rate to be made with a 95% confidence interval of ± 5.2% if the rate is observed to be around 30%, and with a confidence interval of ± 7.3% if the recruitment rate is observed to be around 50%. In addition, the sample of 90 recruited patients will allow the estimate of the mortality rate to be made with a 95% confidence interval of ±9.5% assuming the mortality rate was around 30%. Finally, assuming the recruitment rate was around 30%, a sample of 300 patients approached to take part in the study leading to 90 enrolled patients would allow for the recruitment rate to be estimated with a 95% confidence interval of ± 5.2%. If the recruitment rate was nearer 50%, with 180 patients approached to recruit the 90 enrolled patients, the recruitment rate would be estimated with a 95% confidence interval of ± 7.3%.

Statistical analysis

The analysis will be reported in line with the feasibility studies extension to the CONSORT statement [76]. The aims of the study are to estimate the recruitment, compliance and retention rates to inform the design of a future study and is not powered for hypothesis testing regarding the effectiveness of the intervention. Feasibility outcomes (recruitment, compliance, and retention rates) will be presented with 95% confidence intervals across the whole study population. Compliance and retention rates will also be presented by treatment arm to ensure balanced recruitment, but no formal statistical comparison tests will be made. Mortality and participant dropout rates will be presented with 95% confidence intervals across the whole study population and within treatment arm. Clinical outcome data (secondary outcomes) will be presented as summary statistics using means and standard deviations or medians and ranges/interquartile ranges, as applicable, across the whole study population and by treatment arm. These data will be used

For peer review only

to inform the future trial but will not be used to draw conclusions about the effectiveness of the protocolised early rehabilitation intervention within this study

Trial management

The Chief Investigator (CI) will ensure all study personnel are appropriately orientated and trained, oversee recruitment and report to the trial safety monitoring committee. Training will occur across sites using competency-based training developed at the primary site (University Hospital Southampton NHS Foundation Trust). A study steering group, consisting of an independent chair, expert members and 2 lay advisors will meet every 3-months. Fortnightly teleconferences with trial sites will be held to monitor conduct and progress. Timing and intervals of visits and teleconferences will be reviewed at 3 months to ensure optimal time use.

The CI and Principal Investigators will facilitate local monitoring by the Research and Development quality manager, Research Ethics committee (REC) review and provide access to source data as required. A monitoring report will be produced, summarising the visit, documents and findings. The CI will ensure that all findings are addressed appropriately. The steering group will review all events in a timely manner. Additional monitoring will be scheduled where there is evidence or suspicion of non-compliance with the study protocol.

A Data Management and Safety Committee will be chaired by an independent expert. Quarterly reports will be given to the committee once recruitment has commenced.

Patient and Public Involvement

The study has been supported by patient advisory representatives. These represen3tatives are members of the trial steering committee. Patient advisors partnered with us for the design of the study, the informational material to support the intervention, the burden of the intervention from the patient's perspective and contributed to the dissemination plan

Ethics and dissemination

Ethical approval has been granted by South Central - Hampshire A Research Ethics Committee (REC reference 19/SC/0016). EMPRESS was registered with Clinical Ttrials.gov (ref: NCT03771014) on 10th December, 2018.

Results of this proposed feasibility study will be disseminated for four key audiences: i) patients and public; ii) Intensive care staff, healthcare workers and potential future research delivery partners; iii) service delivery organisations and iv) academic and potential future research collaborators.

Dissemination activities will include feedback to Patients and Public Involvement study focus group, feedback to study participants, presentations to local clinical teams and managers and commissioners

For peer review only

and presentation at conferences attended by appropriate healthcare professionals. Where appropriate, results will be published in peer reviewed journals.

Safety and adverse events

Early mobility within ICUs is safe. In a review of physiotherapy in a critical care rehabilitation programme, 1110 patients received 5267 rehabilitation sessions physiological abnormalities or potential adverse events occurred in only 6 per 1000 interventions [77]. Mobilisation interventions will only be delivered if patients fit the safety criteria defined in table 1. Similar safety criteria have been used in other ICU rehabilitation studies [78, 79].

All adverse events will be documented. Any intervention will cease according to stopping criteria detailed in Table 1. Any such event will be recorded as an adverse event. The CI will provide a monthly update to the safety monitoring committee. Serious adverse events are events that result in death, are life threatening or require prolonged hospitalisation. Any such event will be reported in accordance with the NHS Health Research Authority guidance.

Discussion:

EMPRESS is a feasibility study to assess if a randomised controlled trial of protocolised rehabilitation with supine cycling can be delivered in ventilated patients in ICUs with differing organisational structures with blinded follow-up assessments. A recent meta-analysis indicated that protocolised rehabilitation significantly reduces duration of mechanical ventilation and ICU length of stay[23]. This is consistent with our findings when we introduced the early rehabilitation programme outlined here in our intensive care unit[45]. Passive cycling commenced on ventilated patients may assist the recovery muscle strength in ICU patients[43] although the overall benefits of leg cycle ergometry in the critically ill is inconclusive[44]. We describe a protocolised rehabilitation programme with supine cycling delivered as close to intubation as possible, at an intensity according to the patients' highest performance capability.

Both patient and organisational issues are recognised to the delivery of early rehabilitation of the critically ill patients [35]. A frequently reported challenge is the lack of appropriately qualified staff [80]. This study evaluates the safety, feasibility, effectiveness of delivery and cost efficiency of using therapy technicians to deliver protocolised rehabilitation interventions. In addition to the clinical benefits, early physical rehabilitation can also be cost saving[49]. Even with the employment of additional therapy technicians specifically to assist in the delivery of we have found this early rehabilitation programme cost effective [81].

This study will collect data on the dose of intervention delivered to all patients, reasons for non-delivery of protocol interventions, and the level of experience of therapists delivering the interventions. A qualitative process evaluation is designed to identify both patient and organisational challenges that have potential to be addressed in a potential future study. Findings will inform refinement of trial design and evaluation of the intervention, clarifying causal mechanisms behind study outcomes and providing additional context not adequately captured by the quantitative data. The process evaluation will be consistent with Medical Research Council guidance for conducting process evaluations of complex healthcare interventions[82].

Targeted sedation is embedded within this protocol as oversedation is one of the more commonly cited barriers to mobilisation of the ventilated patient[35]. This study opened to recruitment prior to the publication of the recommended core outcome set for critical care ventilation trials [83] however three of the six outcomes listed (duration of mechanical ventilation, duration of stay and health related quality of life) are secondary outcomes in this study and the other 3 outcomes are included in the data collected This will be addressed should we proceed to a full RCT. Due to the nature of the intervention, it is not possible for this to be blinded however the follow-up assessments will be carried out by a blinded.

Results from EMPRESS will inform the design of a multi-centred RCT, both identifying barriers to the implementation of the designed protocol and exploring how these may be addressed from feedback from the therapy and nursing teams in addition to the feedback from patients and their next of kin.

Acknowledgements

Contributors: RJC and AB contributed equally to the preparation of the paper. RJC and ZvW had the original idea for the study, RJC, LD, IR, NH, AD, GS, ID, MPWG developed the trial protocol, IR devised the statistical analysis plan, MC developed the economic analysis. AB, GS, ID and RJC prepared and submitted documents for Research and Development and ethical approval. RJC, KM and AB wrote the manuscript. All authors reviewed the final version.

Funding: This is supported by the National Institute for Health Research (NIHR) under its Research for Patient Benefit (RfPB) Programme. Grant Reference Number PB-PG-0317-20045. KM is an NIHR Senior Nurse and Midwife Research Leader. The views expressed in this article are those of the author(s) and not necessarily those of the NIHR, or the Department of Health and Social Care

Competing Interests: None declared

For peer review only

Ethics Approval: The study has ethical approval from South Central Hampshire A Research Ethics Committee (19/SC/0016). Protocol Version 1.3 7th Feb 2019

Provenance and Peer review: Not commissioned. Protocol peer-reviewed for ethical and funding application

Disclaimer: The views expressed are those of the author(s) and not necessarily those of the NIHR, NHS or the Department of Health and Social Care.

For peer review only

Table 2 Schedule of assessments

	Randomisation	Day 1	Day 3	Day 7	Awakening	Weekly	ICU Discharge	Hospital Discharge	3 months post hospital discharge
**Demographic Data**	X								
**Muscle assessment:**									
MRCss [60,61]					X	X	X	X	
Grip strength [62]					X	X	X	X	
**Physical function:**									
CPAX [63]		X	X	X	X	X	X		
ICU mobility [64]		X	X	X	X	X	X		
PFITs [59]					X	X	X		
Timed-Up and Go (TUG)							X	X	X
Clinical Frailty Score [69]		(X)					X	X	X
Barthel Index		(X)					X	X	X
6-minute walk test [70]								X	X
**HRQL:**									
WHODAS 2 [71]									X
HADS [72,73]									X
EQ5D-5L [74]									X
Impact of Event Scale [75]									X
Health Economic Evaluation (CSRI)*									X

Physical Function ICU Test-scored (PFITs); Medical Research Council Manual Muscle Test Sum Score (MRC-ss); Chelsea Critical Care Assessment Tool (CPAX); World Health Organisation Disability Assessment; Euroqol 5-dimension 5 level health related quality of life questionnaire(EQ5D-5L); Hospital anxiety and depression scale (HADs); Client service receipt inventory (CSRI)

Figure 1: Study design

Figure 2: Consent pathway

Figure 3: Study intervention pathway

References

1. Hospital Admitted Patient Care Activity 2018-19: NHS England; 2019 [National stastics]. Available at: https://digital.nhs.uk/data-and-information/publications/statistical/hospital-admitted-patient-care-activity/2018-19.[Acessed 21st Aug 2021]
2. Esteban A, Frutos-Vivar F, Muriel A, et al. Evolution of Mortality over Time in Patients Receiving Mechanical Ventilation. *American Journal of Respiratory and Critical Care Medicine* 2013;188(2):220-30. doi: 10.1164/rccm.201212-2169oc
3. Kaukonen KM, Bailey M, Suzuki S, et al. Mortality Related to Severe Sepsis and Septic Shock Among Critically Ill Patients in Australia and New Zealand, 2000-2012. *Jama-J Am Med Assoc* 2014;311(13):1308-16. doi: 10.1001/jama.2014.2637
4. Iwashyna TJ, Netzer G. The Burdens of Survivorship: An Approach to Thinking about Long-Term Outcomes after Critical Illness. *Seminars in Respiratory and Critical Care Medicine* 2012;33(4):327-38. doi: 10.1055/s-0032-1321982
5. Cheung AM, Tansey CM, Tomlinson G, et al. Two-year outcomes, health care use, and costs of survivors of acute respiratory distress syndrome. *American Journal of Respiratory and Critical Care Medicine* 2006;174(5):538-44.
6. Cuthbertson BH, Roughton S, Jenkinson D, et al. Quality of life in the five years after intensive care: a cohort study. *Critical Care* 2010;14(1) doi: 10.1186/cc8848
7. Desai SV, Law TJ, Needham DM. Long-term complications of critical care. *Critical Care Medicine* 2011;39(2):371-79.
8. Griffiths J, Hatch RA, Bishop J, et al. An exploration of social and economic outcome and associated health-related quality of life after critical illness in general intensive care unit survivors: a 12-month follow-up study. *Critical Care* 2013;17(3) doi: 10.1186/cc12745
9. Herridge MS, Tansey CM, Matte A, et al. Functional Disability 5 Years after Acute Respiratory Distress Syndrome. *New England Journal of Medicine* 2011;364(14):1293-304. doi: 10.1056/NEJMoa1011802
10. Iwashyna TJ, Ely EW, Smith DM, et al. Long-term Cognitive Impairment and Functional Disability Among Survivors of Severe Sepsis. *Jama-J Am Med Assoc* 2010;304(16):1787-94. doi: 10.1001/jama.2010.1553
11. Lone NI, Gillies MA, Haddow C, et al. Five-Year Mortality and Hospital Costs Associated with Surviving Intensive Care. *American Journal of Respiratory and Critical Care Medicine* 2016;194(2):198-208. doi: 10.1164/rccm.201511-2234OC
12. Allen C, Glasziou P, Del Mar C. Bed rest: a potentially harmful treatment needing more careful evaluation. *Lancet* 1999;354(9186):1229-33. doi: 10.1016/s0140-6736(98)10063-6
13. Jolley SE, Bunnell AE, Hough CL. ICU-Acquired Weakness. *Chest* 2016;150(5):1129-40. doi: 10.1016/j.chest.2016.03.045 [published Online First: 2016/04/07]
14. Parry SM, Puthucheary Z. The impact of extended bed rest on the musculoskeletal system in the critical care environment. *Extreme Physiology and Experimental Medicine* 2015;4
15. Puthucheary ZA, Rawal J, McPhail M, et al. Acute Skeletal Muscle Wasting in Critical Illness. *Jama-J Am Med Assoc* 2013;310(15):1591-600. doi: 10.1001/jama.2013.278481

For peer review only

16. Levine S, Nguyen T, Taylor N, et al. Rapid Disuse Atrophy of Diaphragm Fibers in Mechanically Ventilated Humans. *New England Journal of Medicine* 2008;358(13):1327-35. doi: 10.1056/NEJMoa070447
17. Needham DM. Mobilizing patients in the intensive care unit - Improving neuromuscular weakness and physical function. *Jama-J Am Med Assoc* 2008;300(14):1685-90. doi: 10.1001/jama.300.14.1685
18. Kress JP, Hall JB. CRITICAL CARE MEDICINE ICU-Acquired Weakness and Recovery from Critical Illness. *New England Journal of Medicine* 2014;370(17):1626-35. doi: 10.1056/NEJMra1209390
19. Hodgson CL, Berney S, Harrold M, et al. Clinical review: early patient mobilization in the ICU. *Crit Care* 2013;17(1):207. doi: 10.1186/cc11820 [published Online First: 2013/05/16]
20. Schweickert WD, Pohlman MC, Pohlman AS, et al. Early physical and occupational therapy in mechanically ventilated, critically ill patients: a randomised controlled trial. *Lancet* 2009;373(9678):1874-82. doi: 10.1016/s0140-6736(09)60658-9
21. Schujmann DS, Gomes TT, Lunardi AC, et al. Impact of a Progressive Mobility Program on the Functional Status, Respiratory, and Muscular Systems of ICU Patients: A Randomized and Controlled Trial*. *Critical Care Medicine* 2020;48(4):491-97. doi: 10.1097/ccm.0000000000004181
22. Calvo-Ayala E, Khan BA, Farber MO, et al. Interventions to Improve the Physical Function of ICU Survivors A Systematic Review. *Chest* 2013;144(5):1469-80. doi: 10.1378/chest.13-0779
23. Waldauf P, Jiroutkova K, Krajcova A, et al. Effects of Rehabilitation Interventions on Clinical Outcomes in Critically Ill Patients: Systematic Review and Meta-Analysis of Randomized Controlled Trials*. *Critical Care Medicine* 2020;48(7):1055-65. doi: 10.1097/ccm.0000000000004382
24. Kayambu G, Boots R, Paratz J. Physical Therapy for the Critically Ill in the ICU: A Systematic Review and Meta-Analysis. *Critical Care Medicine* 2013;41(6):1543-54. doi: 10.1097/CCM.0b013e31827ca637
25. Berney SC, Harrold M, Webb SA, et al. Intensive care unit mobility practices in Australia and New Zealand: a point prevalence study. *Critical Care and Resuscitation* 2013;15(4):260-65.
26. Harrold ME, Salisbury LG, Webb SA, et al. Early mobilisation in intensive care units in Australia and Scotland: a prospective, observational cohort study examining mobilisation practises and barriers. *Critical Care* 2015;19 doi: 10.1186/s13054-015-1033-3
27. Nydahl P, Ruhl AP, Bartoszek G, et al. Early Mobilization of Mechanically Ventilated Patients: A 1-Day Point-Prevalence Study in Germany*. *Critical Care Medicine* 2014;42(5):1178-86. doi: 10.1097/ccm.0000000000000149
28. McWilliams D, Jones C, Atkins G, et al. Earlier and enhanced rehabilitation of mechanically ventilated patients in critical care: A feasibility randomised controlled trial. *J Crit Care* 2018;44:407-12. doi: 10.1016/j.jcrc.2018.01.001 [published Online First: 2018/01/15]
29. Wright SE, Thomas K, Watson G, et al. Intensive versus standard physical rehabilitation therapy in the critically ill (EPICC): a multicentre, parallel-group, randomised controlled trial. *Thorax* 2018;73(3):213-21. doi: 10.1136/thoraxjnl-2016-209858
30. Denehy L, Skinner EH, Edbrooke L, et al. Exercise rehabilitation for patients with critical illness: a randomized controlled trial with 12 months of follow-up. *Critical Care* 2013;17(4) doi: 10.1186/cc12835
31. Moss M, Nordon-Craft A, Malone D, et al. A Randomized Trial of an Intensive Physical Therapy Program for Patients with Acute Respiratory Failure. *Am J Respir Crit Care Med* 2016;193(10):1101-10. doi: 10.1164/rccm.201505-1039OC [published Online First: 2015/12/15]
32. Walsh TS, Salisbury LG, Boyd J, et al. A randomised controlled trial evaluating a rehabilitation complex intervention for patients following intensive care discharge: the RECOVER study. *Bmj Open* 2012;2(4) doi: ARTN e001475 10.1136/bmjopen-2012-001475

For peer review only

33. Cuthbertson BH, Rattray J, Campbell MK, et al. The PRaCTICaL study of nurse led, intensive care follow-up programmes for improving long term outcomes from critical illness: a pragmatic randomised controlled trial. *BMJ* 2009;339(oct16 1):b3723-b23. doi: 10.1136/bmj.b3723
34. Elliott D, McKinley S, Alison J, et al. Health-related quality of life and physical recovery after a critical illness: a multi-centre randomised controlled trial of a home-based physical rehabilitation program. *Critical Care* 2011;15(3) doi: 10.1186/cc10265
35. Dubb R, Nydahl P, Hermes C, et al. Barriers and Strategies for Early Mobilization of Patients in Intensive Care Units. *Ann Am Thorac Soc* 2016;13(5):724-30. doi: 10.1513/AnnalsATS.201509-586CME
36. Nydahl P, Sricharoenchai T, Chandra S, et al. Safety of Patient Mobilization and Rehabilitation in the Intensive Care Unit Systematic Review with Meta-Analysis. *Ann Am Thorac Soc* 2017;14(5):766-77. doi: 10.1513/AnnalsATS.201611-843SR
37. Kho ME, Martin RA, Toonstra AL, et al. Feasibility and safety of in-bed cycling for physical rehabilitation in the intensive care unit. *J Crit Care* 2015;30(6):1419.e1-5. doi: 10.1016/j.jcrc.2015.07.025 [published Online First: 2015/09/01]
38. Sricharoenchai T, Parker AM, Zanni JM, et al. Safety of physical therapy interventions in critically ill patients: A single-center prospective evaluation of 1110 intensive care unit admissions. *Journal of Critical Care* 2014;29(3):395-400. doi: 10.1016/j.jcrc.2013.12.012
39. Kho ME, Molloy AJ, Clarke FJ, et al. TryCYCLE: A Prospective Study of the Safety and Feasibility of Early In-Bed Cycling in Mechanically Ventilated Patients. *Plos One* 2016;11(12) doi: 10.1371/journal.pone.0167561
40. Camargo Pires-Neto R, Fogaça Kawaguchi YM, Sayuri Hirota A, et al. Very early passive cycling exercise in mechanically ventilated critically ill patients: physiological and safety aspects--a case series. *Plos One* 2013;8(9):e74182. doi: 10.1371/journal.pone.0074182 [published Online First: 2013/09/17]
41. Wilkinson OM, Bates A, Cusack R. An observational feasibility study - does early limb ergometry affect oxygen delivery and uptake in intubated critically ill patients - a comparison of two assessment methods. *BMC Anesthesiol* 2021;21(1):27. doi: 10.1186/s12871-020-01227-z [published Online First: 2021/01/27]
42. Hickmann CE, Castanares-Zapatero D, Deldicque L, et al. Impact of Very Early Physical Therapy During Septic Shock on Skeletal Muscle: A Randomized Controlled Trial. *Crit Care Med* 2018;46(9):1436-43. doi: 10.1097/ccm.0000000000003263 [published Online First: 2018/06/30]
43. Machado ADS, Pires-Neto RC, Carvalho MTX, et al. Effects that passive cycling exercise have on muscle strength, duration of mechanical ventilation, and length of hospital stay in critically ill patients: a randomized clinical trial. *J Bras Pneumol* 2017;43(2):134-39. doi: 10.1590/s1806-37562016000000170 [published Online First: 2017/05/26]
44. Takaoka A, Utgikar R, Rochwerg B, et al. The Efficacy and Safety of In-Intensive Care Unit Leg-Cycle Ergometry in Critically Ill Adults A Systematic Review and Meta-analysis. *Ann Am Thorac Soc* 2020;17(10):1289-307. doi: 10.1513/AnnalsATS.202001-059OC
45. van Willigen Z, Collings N, Richardson D, et al. Quality improvement: The delivery of true early mobilisation in an intensive care unit. *BMJ Qual Improv Rep* 2016;5(1):u211734.w4726. doi: 10.1136/bmjquality.u211734.w4726
46. Needham DM, Korupolu R, Zanni JM, et al. Early Physical Medicine and Rehabilitation for Patients With Acute Respiratory Failure: A Quality Improvement Project. *Arch Phys Med Rehab* 2010;91(4):536-42. doi: 10.1016/j.apmr.2010.01.002
47. Morris PE, Goad A, Thompson C, et al. Early intensive care unit mobility therapy in the treatment of acute respiratory failure. *Critical Care Medicine* 2008;36(8):2238-43. doi: 10.1097/CCM.0b013e318180b90e

For peer review only

48. McWilliams D, Weblin J, Atkins G, et al. Enhancing rehabilitation of mechanically ventilated patients in the intensive care unit: A quality improvement project. *Journal of Critical Care* 2015;30(1):13-18. doi: 10.1016/j.jcrc.2014.09.018
49. Lord RK, Mayhew CR, Korupolu R, et al. ICU Early Physical Rehabilitation Programs: Financial Modeling of Cost Savings. *Critical Care Medicine* 2013;41(3):717-24. doi: 10.1097/CCM.0b013e3182711de2
50. Chan A-W, Tetzlaff JM, Altman DG, et al. SPIRIT 2013 Statement: Defining Standard Protocol Items for Clinical Trials. *Annals of Internal Medicine* 2013;158(3):200-+. doi: 10.7326/0003-4819-158-3-201302050-00583
51. Hoffmann TC, Glasziou PP, Boutron I, et al. Better reporting of interventions: template for intervention description and replication (TIDieR) checklist and guide. *BMJ : British Medical Journal* 2014;348:g1687. doi: 10.1136/bmj.g1687
52. Mental Capacity Act, 2005.Availble at 2005. Legislation.gov.uk, Available at: http://www.legislation.gov.uk/ukpga/2005/9/contents [Accessed 7 Dec 2021].
53. Ely EW, Truman B, Shintani A, et al. Monitoring Sedation Status Over Time in ICU PatientsReliability and Validity of the Richmond Agitation-Sedation Scale (RASS). *JAMA* 2003;289(22):2983-91. doi: 10.1001/jama.289.22.2983
54. Sessler CN, Gosnell MS, Grap MJ, et al. The Richmond Agitation–Sedation Scale. *American Journal of Respiratory and Critical Care Medicine* 2002;166(10):1338-44. doi: 10.1164/rccm.2107138
55. Ely EW, Inouye SK, Bernard GR, et al. Delirium in mechanically ventilated patients - Validity and reliability of the Confusion Assessment Method for the intensive care unit (CAM-ICU). *Jama-J Am Med Assoc* 2001;286(21):2703-10. doi: 10.1001/jama.286.21.2703
56. De Jonghe B, Sharshar T, Lefaucheur JP, et al. Paresis acquired in the intensive care unit - A prospective multicenter study. *Jama-J Am Med Assoc* 2002;288(22):2859-67. doi: 10.1001/jama.288.22.2859
57. Denehy L, de Morton NA, Skinner EH, et al. A physical function test for use in the intensive care unit: validity, responsiveness, and predictive utility of the physical function ICU test (scored). *Phys Ther* 2013;93(12):1636-45. doi: 10.2522/ptj.20120310 [published Online First: 2013/07/28]
58. Skinner EH, Berney S, Warrillow S, et al. Development of a physical function outcome measure (PFIT) and a pilot exercise training protocol for use in intensive care. *Crit Care Resusc* 2009;11(2):110-5. [published Online First: 2009/06/03]
59. Fan E, Ciesla ND, Truong AD, et al. Inter-rater reliability of manual muscle strength testing in ICU survivors and simulated patients. *Intensive Care Med* 2010;36(6):1038-43. doi: 10.1007/s00134-010-1796-6 [published Online First: 2010/03/10]
60. Hermans G, Clerckx B, Vanhullebusch T, et al. Interobserver agreement of Medical Research Council sum-score and handgrip strength in the intensive care unit. *Muscle Nerve* 2012;45(1):18-25. doi: 10.1002/mus.22219 [published Online First: 2011/12/23]
61. Vanpee G, Segers J, Van Mechelen H, et al. The interobserver agreement of handheld dynamometry for muscle strength assessment in critically ill patients. *Crit Care Med* 2011;39(8):1929-34. doi: 10.1097/CCM.0b013e31821f050b [published Online First: 2011/05/17]
62. Corner EJ, Wood H, Englebretsen C, et al. The Chelsea critical care physical assessment tool (CPAx): validation of an innovative new tool to measure physical morbidity in the general adult critical care population; an observational proof-of-concept pilot study. *Physiotherapy* 2013;99(1):33-41. doi: 10.1016/j.physio.2012.01.003 [published Online First: 2012/12/12]
63. Hodgson C, Needham D, Haines K, et al. Feasibility and inter-rater reliability of the ICU Mobility Scale. *Heart Lung* 2014;43(1):19-24. doi: 10.1016/j.hrtlng.2013.11.003 [published Online First: 2014/01/01]
64. Bischoff HA, Stähelin HB, Monsch AU, et al. Identifying a cut-off point for normal mobility: a comparison of the timed 'up and go' test in community-dwelling and institutionalised elderly

For peer review only

women. *Age Ageing* 2003;32(3):315-20. doi: 10.1093/ageing/32.3.315 [published Online First: 2003/05/02]
65. Ng SS, Hui-Chan CW. The timed up & go test: its reliability and association with lower-limb impairments and locomotor capacities in people with chronic stroke. *Arch Phys Med Rehabil* 2005;86(8):1641-7. doi: 10.1016/j.apmr.2005.01.011 [published Online First: 2005/08/09]
66. Bagshaw M, Majumdar SR, Rolfson DB, et al. A prospective multicenter cohort study of frailty in younger critically ill patients. *Crit Care* 2016;20(1):175. doi: 10.1186/s13054-016-1338-x [published Online First: 2016/06/07]
67. Bagshaw SM, Stelfox HT, Johnson JA, et al. Long-term association between frailty and health-related quality of life among survivors of critical illness: a prospective multicenter cohort study. *Crit Care Med* 2015;43(5):973-82. doi: 10.1097/ccm.0000000000000860 [published Online First: 2015/02/11]
68. Rockwood K, Song X, MacKnight C, et al. A global clinical measure of fitness and frailty in elderly people. *Cmaj* 2005;173(5):489-95. doi: 10.1503/cmaj.050051 [published Online First: 2005/09/01]
69. ATS statement: guidelines for the six-minute walk test. *Am J Respir Crit Care Med* 2002;166(1):111-7. doi: 10.1164/ajrccm.166.1.at1102 [published Online First: 2002/07/02]
70. Ustun TB, Kostanjesek N, Chatterji S, et al. Measuring health and disability : manual for WHO Disability Assessment Schedule (WHODAS 2.0) / edited by T.B. Üstün, N. Kostanjsek, S. Chatterji, J.Rehm. Geneva: World Health Organization, 2010.
71. Bjelland I, Dahl AA, Haug TT, et al. The validity of the Hospital Anxiety and Depression Scale. An updated literature review. *J Psychosom Res* 2002;52(2):69-77. doi: 10.1016/s0022-3999(01)00296-3 [published Online First: 2002/02/08]
72. Zigmond AS, Snaith RP. The hospital anxiety and depression scale. *Acta Psychiatr Scand* 1983;67(6):361-70. doi: 10.1111/j.1600-0447.1983.tb09716.x [published Online First: 1983/06/01]
73. Herdman M, Gudex C, Lloyd A, et al. Development and preliminary testing of the new five-level version of EQ-5D (EQ-5D-5L). *Qual Life Res* 2011;20(10):1727-36. doi: 10.1007/s11136-011-9903-x [published Online First: 2011/04/12]
74. Schwarzwald J, Solomon Z, Weisenberg M, et al. Validation of the Impact of Event Scale for psychological sequelae of combat. *J Consult Clin Psychol* 1987;55(2):251-6. doi: 10.1037//0022-006x.55.2.251 [published Online First: 1987/04/01]
75. Proctor E, Silmere H, Raghavan R, et al. Outcomes for Implementation Research: Conceptual Distinctions, Measurement Challenges, and Research Agenda. *Administration and Policy in Mental Health and Mental Health Services Research* 2011;38(2):65-76. doi: 10.1007/s10488-010-0319-7
76. Eldridge SM, Chan CL, Campbell MJ, et al. CONSORT 2010 statement: extension to randomised pilot and feasibility trials. *BMJ* 2016;355:i5239. doi: 10.1136/bmj.i5239
77. Sricharoenchai T, Parker AM, Zanni JM, et al. Safety of physical therapy interventions in critically ill patients: a single-center prospective evaluation of 1110 intensive care unit admissions. *J Crit Care* 2014;29(3):395-400. doi: 10.1016/j.jcrc.2013.12.012 [published Online First: 2014/02/11]
78. Kho ME, Molloy AJ, Clarke F, et al. CYCLE pilot: a protocol for a pilot randomised study of early cycle ergometry versus routine physiotherapy in mechanically ventilated patients. *Bmj Open* 2016;6(4) doi: 10.1136/bmjopen-2016-011659
79. Kayambu G, Boots RJ, Paratz JD. Early rehabilitation in sepsis: a prospective randomised controlled trial investigating functional and physiological outcomes The i-PERFORM Trial (Protocol Article). *BMC Anesthesiol* 2011;11:21. doi: 10.1186/1471-2253-11-21 [published Online First: 2011/11/01]
80. Parry SM, Knight LD, Connolly B, et al. Factors influencing physical activity and rehabilitation in survivors of critical illness: a systematic review of quantitative and qualitative studies.

For peer review only

Intensive Care Med 2017;43(4):531-42. doi: 10.1007/s00134-017-4685-4 [published Online First: 2017/02/18]

81. van Willigen Z, Gallagher L, Cusack R, et al. NEW ROLES ON ICU: THE COST EFFECTIVENESS OF EMPLOYING THERAPY TECHNICIANS ON INTENSIVE CARE TO REDUCE LENGTH OF STAY AND IMPROVE FUNCTIONAL OUTCOMES. *Intens Care Med* 2013;39:S488-S88.
82. Skivington K, Matthews L, Simpson SA, et al. A new framework for developing and evaluating complex interventions: update of Medical Research Council guidance. *BMJ* 2021;374:n2061. doi: 10.1136/bmj.n2061
83. Blackwood B, Ringrow S, Clarke M, et al. A Core Outcome Set for Critical Care Ventilation Trials. *Critical care medicine* 2019;47(10):1324-31. doi: 10.1097/CCM.0000000000003904

For peer review only

Figure 1 Study design

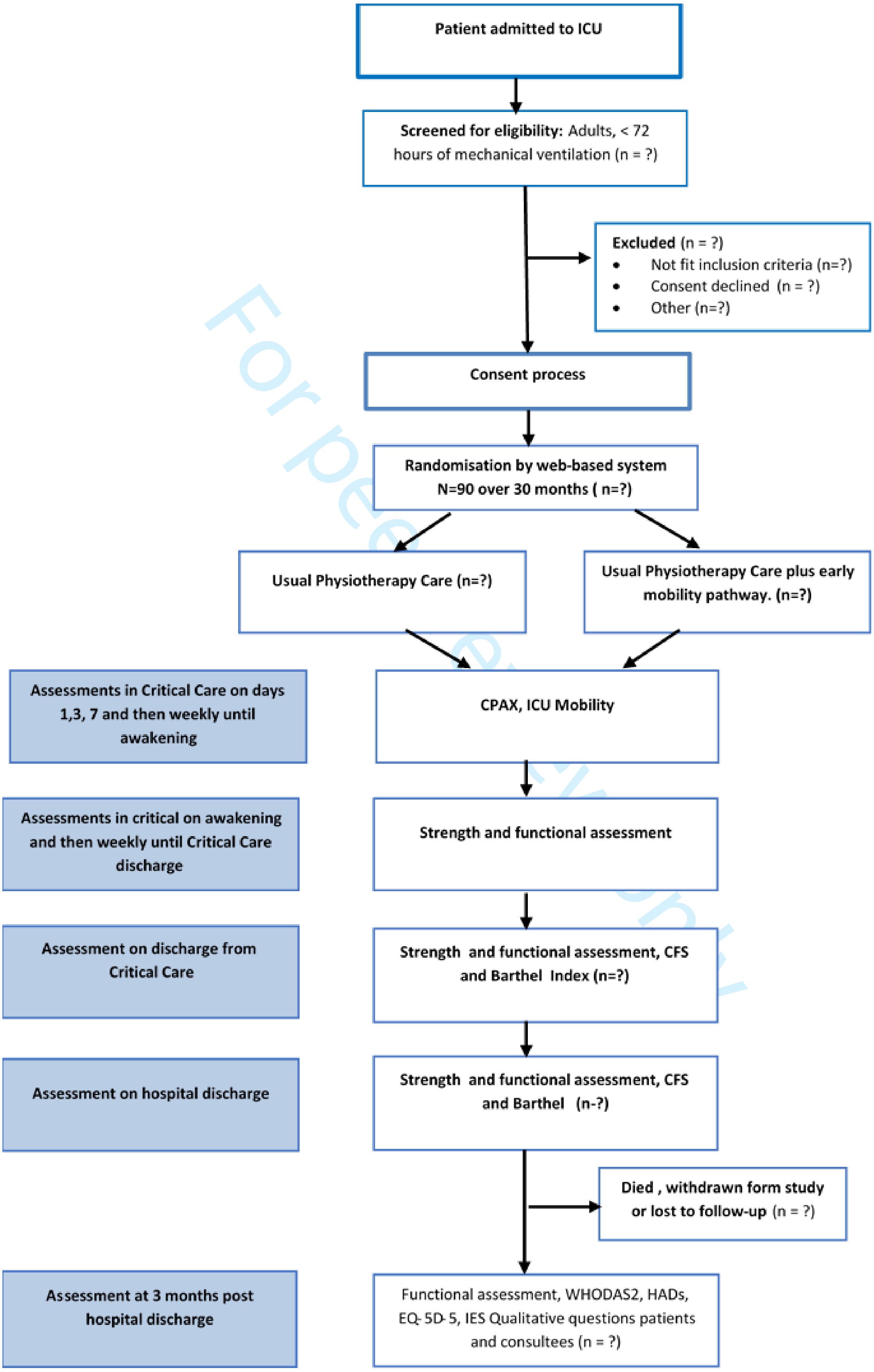

Figure 2 Consent pathway

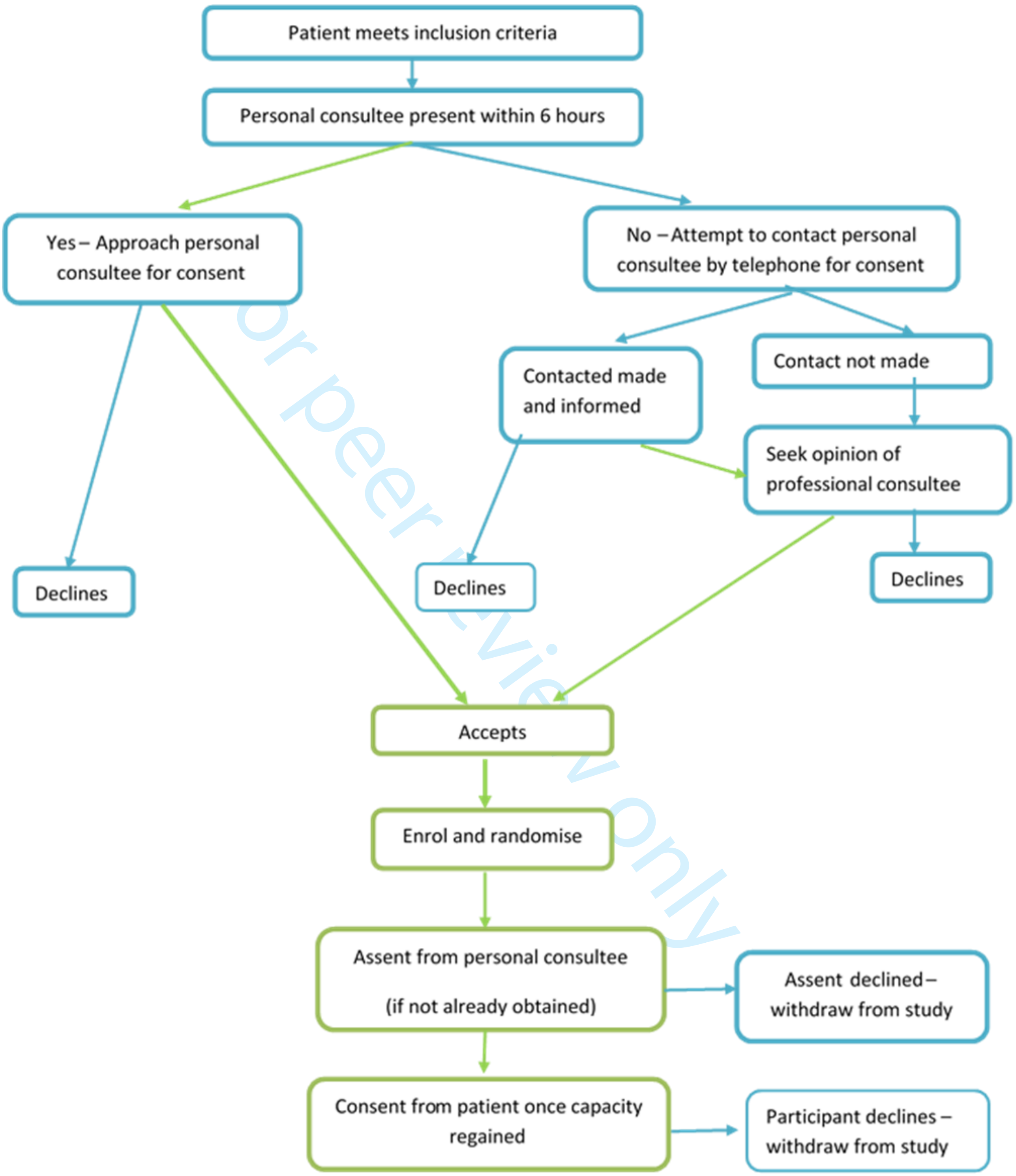

Figure 3 Study Intervention Pathway

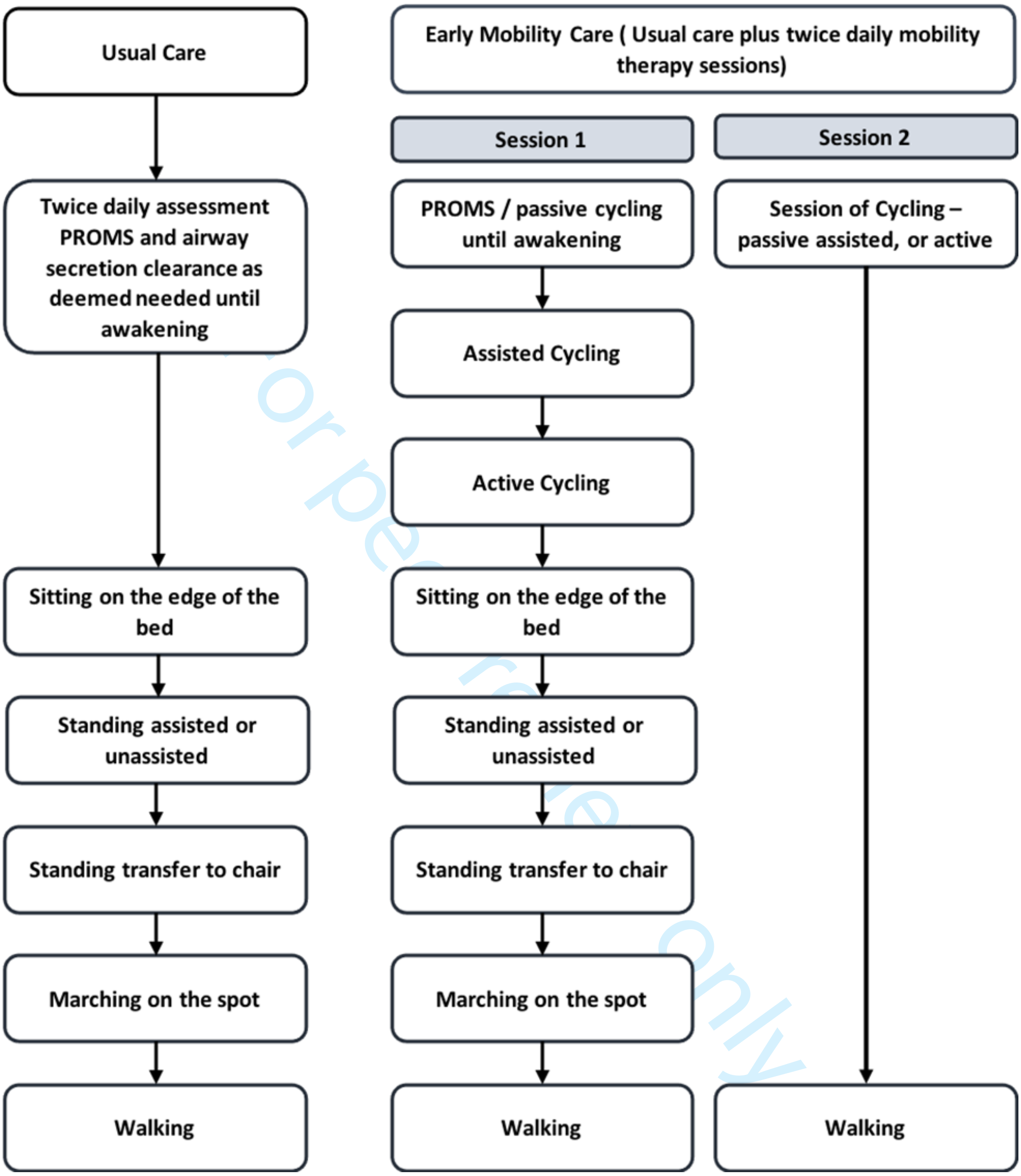

Site specific header to be inserted here

EMPRESS.

A feasibility study of early mobilisation in Critical Care.

Patient Information Sheet

Version 2: 29th January 2019

Introduction

Study Title: EMPRESS: A study of very early mobilisation in Critical Care.

Invitation:

Your consultee has agreed on your behalf, to your participation on a research study. We would like to invite you to confirm whether you wish to continue or withdraw your participation from this research study.

This hospital is taking part in a national research study to investigate whether starting rehabilitation in the Intensive Care Unit, as soon as possible, will improve patient's long-term physical ability and quality of life.

When patients are sedated in Intensive Care, muscle wasting and weakness can occur very quickly and this can take a long time to recover from. Because we feel that it may be important to deliver rehabilitation physiotherapy as early as possible, it was agreed by your doctor and / or your relative/ friend that you could be involved in this study. This research has been approved by Hampshire Research Ethics Committee (IRAS number: 250165).

This patient information sheet provides information about the study to help you decide if you would like to continue to participate in it. It is important that you understand why the research is being done and what it involves.

Knowing what is involved will help you decide if you want to continue to take part in the research, so this Information Sheet explains the tests and treatments involved.

- Part 1 tells you about why we are doing this study and what will happen to you if you continue to take part.
- Part 2 gives you more detailed information about how we will run the study.

If you have no objection to continue taking part, we will ask you to read and sign a form that records your permission, called the consent declaration. We'll then give you a copy to keep. We will keep you fully informed during the study so you can let us know if you have any concerns or if you would prefer to be withdrawn. Taking part in this research is entirely voluntary. If you decide not to continue, you will still be offered the best possible standard of care.

Please ask us if there is anything that is not clear or if you would like more information. Take time to decide whether or not you wish to take part.

For peer review only - http://bmjopen.bmj.com/site/about/guidelines.xhtml

PART ONE: Why are we doing this study and what will happen to you?

What is the purpose of this study?

We know that when patients are very unwell and need sedation in the Intensive Care Unit, they can lose muscle strength and size very quickly. It is normal to offer rehabilitation, but this often starts after the patient has woken up. By this time the muscles have already been affected. Previous studies have shown that this can take many months to recover from and may affect a patient's quality of life after leaving hospital.

In Southampton Hospital, researchers and physiotherapists started performing rehabilitation exercises much earlier than usual, even while the patient was sedated. They showed that this method reduced the patient's time on the ventilator and reduced the amount of time that they needed to be in Intensive Care.

We are now trying to discover whether this method will work in a number of different hospitals in the UK. We will also do some tests to see whether the patients who have this type of rehabilitation are stronger and able to engage in physical activity more easily, when they leave hospital and 3 months later.

Why have you been chosen?

You were enrolled in this study because during your admission to the Intensive Care you needed a ventilator (a machine to help you breathe) and sedation was needed to help keep you calm and comfortable. The treating doctor and physiotherapist thought that either very early rehabilitation or standard rehabilitation would be equally suitable. We may have given you very early physiotherapy already in the intensive care unit, because we are testing such early rehabilitation, but we would like to ask for your permission to continue.

Do I have to take part?

No. It is up to you to decide whether or not you would like to continue to take part. If you do, you will be given this information sheet to keep and be asked to sign the consent form. You are still free to withdraw at any time without giving a reason. The decision to withdraw at any time, or a decision not to take part, will not affect the standard of care you receive.

What will happen to me if I take part?

Participation in the study began in the Intensive Care Unit. The final tests will take place 3 months after you leave hospital.

In the Intensive Care Unit: Your treating doctor has assessed you to be eligible to take part in this study: EMPRESS. You were randomly allocated (like the flip of a coin) to receive either of the following:

- **Standard physiotherapy:** All patients on the trial will receive their normal physiotherapy. This will normally include activities to assist in keeping your airway

For peer review only - http://bmjopen.bmj.com/site/about/guidelines.xhtml

clear and activities to maintain limb flexibility. These will not be affected by being on the trial.

OR

- **Standard physiotherapy, as above, plus an extra 2 sessions of 30 minutes of rehabilitation from Monday to Friday.** For patients receiving extra, early rehabilitation, in addition to your normal physiotherapy, you have been using a cycle machine that is designed to work, in the bed, even with sedated patients. As you wake up you have started to pedal for yourself, do some more bed-based exercises and finally get out of bed and start moving. All of these sessions have been and will continue to be run by a well-trained physiotherapist and the bedside nurses. We have already tested this method in University Hospital Southampton and it has reduced the length of time on the ventilator and ICU stay. During these sessions, you have been and will continue to be very carefully monitored for your own safety and the safety of lines, tubes and catheters.

  These exercises will continue for a maximum of 28 days or less if you leave the Intensive care unit before then.

BOTH GROUPS

- **Additional assessments:** So that we can test whether our new method works, patients on the trial will undertake some extra assessments. These include a simple test of grip strength by using a hand held pressure monitor; a test of arm and leg strength, ability to stand and step and mobility and walking tests. There will also be quality of life and health questionnaires.

There was a 50/50 chance of being allocated to either group. Neither you nor your doctor can decide which. No samples of blood are required for this research study.

In the hospital ward: When you have been discharged to a normal hospital ward, you won't receive any extra physiotherapy. Just before you go home, you will be tested again for muscle strength and mobility, including how far you can walk in 6 minutes. These tests will be supervised by a trained and experienced physiotherapist

Following discharge from hospital: Regardless of which group you were allocated to, after going home, you should follow the advice given to you by your doctors and physiotherapists. We have designed our study so that this will not affect our results.

You will be contacted by one of the critical care research team 3 months after you have been discharged home. We will arrange to see you for approximately one hour. During this visit we will test your walking speed, strength and agility. We will also ask for some questionnaires to be completed, which will assess how you feel about your quality of life and recovery.

The researchers would also like to have access to your medical record to obtain information relevant to the study. This information would be anonymised and kept confidential.

If you have any questions regarding the trial procedures, please don't hesitate to ask the intensive care or research doctors, physiotherapists and nurses.

For peer review only - http://bmjopen.bmj.com/site/about/guidelines.xhtml

What do I have to do?

It is important to tell the doctor and the research staff about any treatments or medications you may have been taking, including over-the-counter medications, vitamins or herbal remedies, acupuncture or other alternative treatments. We would also like to know about any medical conditions which may affect the exercise.

Please let us know if you are involved in any other studies at this time.

What are the alternatives to participation?

Participation in this research is not the only option. You may decide to receive only standard or usual care. This is absolutely fine. Please feel free to discuss these options with your doctor before deciding whether or not to continue to take part in this research project.

What are the possible disadvantages of taking part?

Early mobility within ICU is safe. Potential risks may include, but not be limited to blood pressure or heart rate problems, breathing problems, problems with the tubes, lines and catheters.

In a review of physiotherapy within Intensive Care Units, involving over 1100 patients and 5267 episodes of physiotherapy in similar patients, there were 34 potential safety events (equivalent to 6 events in 1000 episodes of physiotherapy), Most of these were potentially related to changes in heart rate or blood pressure which settle quickly in stopping the physiotherapy.

In Southampton, over a four year period, we have treated over 500 patients in this way and had 2 non-serious adverse events.

The doctors, physiotherapists and nurses who will be caring for you while in the ICU, are trained to recognise the effects on the body associated with physical rehabilitation and will treat you accordingly. You will be continue to be monitored and assessed. Your safety is always our number one priority.

There may be side effects that the researchers do not expect or do not know about and that may be serious. Please tell the doctor immediately about any new or unusual symptoms that you get.

What are the possible benefits of taking part?

We cannot guarantee or promise that you will receive any benefits from this research. This study aims to further medical knowledge and may improve future treatment of patients who need to be on a ventilator, however it may not directly benefit you.

For how long will I be in the research study?

The final research assessment will take place 3 months after discharge from hospital. Once that is done, your participation in the study will end.

For peer review only - http://bmjopen.bmj.com/site/about/guidelines.xhtml

What happens if there is a problem?

We will keep you fully informed of any problems which may be related to the study.

Will taking part in the study be kept confidential?

Yes. All of the information about participation and the data collected will be kept confidential.

Information held by the NHS and records maintained by the NHS Information Centre and the NHS Central Register may be used to help contact you and provide information about your health status. This information may be obtained and stored by the study research team to enable long term follow-up.

University Hospital Southampton is the sponsor for this study based in the United Kingdom. We will be using information from you and/or your medical records in order to undertake this study and will act as the data controller for this study. This means that we are responsible for looking after your information and using it properly. University Hospital Southampton will keep information about you for 10 years after the study has finished.

Your rights to access, change or move your information are limited, as we need to manage your information in specific ways in order for the research to be reliable and accurate. If you withdraw from the study, we will keep the information about you that we have already obtained. To safeguard your rights, we will use the minimum personally-identifiable information possible.

You can find out more about how we use your information at https://www.hra.nhs.uk/information-about-patients/

[Local NHS site name] will collect information from you and/or your medical records for this research study in accordance with our instructions.

(Local NHS site name) will keep your name, NHS number and contact details confidential and will not pass this information to University Hospital Southampton. [Local NHS site name] will use this information as needed, to contact you about the research study, and make sure that relevant information about the study is recorded for your care, and to oversee the quality of the study. Certain individuals from University Hospital Southampton and regulatory organisations may look at your medical and research records to check the accuracy of the research study. University Hospital Southampton will only receive information without any identifying information. The people who analyse the information will not be able to identify you and will not be able to find out your name, NHS number or contact details.

[Local NHS site name] will keep identifiable information about you, including the consent form from this study for 10 years after the study has finished.

For peer review only - http://bmjopen.bmj.com/site/about/guidelines.xhtml

Contact Details:

Local PI

Consultant Critical Care,

Hospital address

Dr XXXX: 02381 XXXXXX

Research Nurse: 02381 XXXXXX

ICU: 02381 XXXXXX

PART 2: How we will run this study.

What if relevant new information becomes available?

During the research project, new information about the risks and benefits of the study may become known to the researchers. If this occurs, you will be told about this new information and the doctor will discuss whether this new information affects you.

If any information becomes available which could affect your participation in the study the research doctor will tell you about it and discuss whether you want to continue in the study. If you decide to not continue in the study, the research doctor will make arrangements for your care to continue as normal. If you decide to continue in the study you will be asked to sign an updated consent form.

Also, on receiving new information the research doctor might consider it to be in your best interests to withdraw you from the study. He/she will explain the reasons and arrange for your care to continue.

If the study is stopped for any other reason, you will be told why and your continuing care will be arranged.

What will happen if I don't want to carry on with the study?

You can withdraw from the study at any time without giving an explanation and be assured that it will not impact on any part of your further treatment.

If you decide to withdraw from the study, the researchers would like to keep your health information that has already been collected. This is to help them make sure that the results of the research can be measured properly. If you do not want them to do this, you can tell them when you withdraw from the research project.

What if there is a problem?

If you have any concerns regarding the study, please ask to speak to the ICU doctor in charge of your care or ask to speak to name of local PI, the consultant who is in charge of the study.

For peer review only - http://bmjopen.bmj.com/site/about/guidelines.xhtml

Complaints:

If you have a concern about any aspect of this study, you should ask to speak with the researchers or the Intensive Care doctors and nurses, who will do their best to answer your questions. If you remain unhappy and wish to complain formally, you can do this through the NHS Complaints Procedure. Please localise with your hospital PALS contact details.

Harm:

In the event that something does go wrong and you are harmed during the research study there are no special compensation arrangements. If you are harmed and this is due to someone's negligence then you may have grounds for a legal action for compensation against University Hospital Southampton, but you may have to pay the legal costs. The normal National Health Service complaints mechanisms will still be available to you.

Involvement of the General Practitioner/Family doctor (GP)

If you are agreeable we would like to inform your GP of your participation in the study. If you do not wish for your GP to be informed, please let us know and indicate on the consent form that you do not wish your GP to be informed.

Will taking part in this study be kept confidential?

If you continue with the study, some parts of your medical records and the data collected for the study will be looked at by authorised researchers from University Hospital Southampton and University of Southampton who are sponsoring and organising the research. They may also be looked at by authorised people to check that the study is being carried out correctly. All will have a strict duty of confidentiality to you, as a research participant and we will do our best to meet this duty.

All information that is collected about you during the course of the research will be kept strictly confidential. Any information about you that leaves the hospital will have your name and address removed so that you cannot be recognised from it.

Anonymised data collected during the study may be sent to associated researchers in other countries, where the laws don't protect your privacy to the same extent as the law in the UK but the study team will take all reasonable steps to protect your privacy.

You have the right to check the accuracy of data held about you and correct any errors.

What will happen to the results of the research study?

They will be published in a medical journal, presented at conferences and lay press where possible.

Who is organising and funding the research?

Dr Rebecca Cusack from University Hospital Southampton is the lead researcher, who is organising the research.

For peer review only - http://bmjopen.bmj.com/site/about/guidelines.xhtml

The research is funded by the NHS through the National Institute for Health Research, Research for Patient Benefit scheme.

Who has reviewed the study?

Hampshire Research Ethics Committee (IRAS number: 250165) have reviewed this study and given their approval.

Thank you very much for taking the time to read this information sheet at this very stressful time.

If you have any further questions please ask the doctors in Intensive Care, Dr (local PI) or one of the research team.

If you agree to continuing participation in this study, please keep this information sheet and you will be given a copy of the agreement form that you will be asked to sign.

For peer review only - http://bmjopen.bmj.com/site/about/guidelines.xhtml

FORM TO BE ON SITE SPECIFIC HEADED PAPER

University Hospital Southampton NHS
NHS Foundation Trust

Critical Care, Anaesthesia & Peri-operative Medicine Dept,
Research office CE 93. MP 24,
University Hospital Southampton,
Tremona Road,
Southampton
SO16 6YD

Tel: 023 8120 5308
Fax: 023 8120 5378

For peer review only

Consent form for patients participating in EMPRESS.
A feasibility study of early mobilisation programmes in Critical Care.

Name of Researcher: ___________________________

Please initial box

1. I confirm that I have read and understood the Patient Information Sheet (version ___ Dated ___________) for the EMPRESS study. I have had the opportunity to ask questions about the study and understand what is involved. ☐

2. I have no objection to taking part in the above study. ☐

3. I understand that my participation is voluntary and that I am free to withdraw from the study at any time, without giving any reason and without my care or legal rights being affected. ☐

4. I understand that relevant sections of my medical notes and data collected during the study, may be looked at by individuals from the research team, from regulatory authorities or from the NHS Trust, where it is relevant to my taking part in this research. I give permission for these individuals to have access to my records. ☐

5. I understand that information held by the NHS and records maintained by the NHS Information Centre and the NHS Central Register may be used to help contact me and provide information about my health status. I give permission for this information to be obtained and stored by the study research team to enable long term follow-up. ☐

6. I agree to my GP being informed of my participation in the study. ☐

7. I agree to take part in the above study ☐

For peer review only - http://bmjopen.bmj.com/site/about/guidelines.xhtml

Name of Participant:	Signature:	Date:
____________________	____________________	____________
**Person undertaking consultation (researcher):**	**Signature:**	**Date:**
____________________	____________________	____________

For peer review only

Original *Informed Consent form to be filed in the Investigator Site File.*

1 copy to be given to the patient

1 copy to be filed in the patients' hospital notes.

For peer review only - http://bmjopen.bmj.com/site/about/guidelines.xhtml

Site specific header to be inserted here

EMPRESS.

A feasibility study of early mobilisation in Critical Care.

Information for Consultee

Version 2: 29th January 2019

Introduction

Study Title: EMPRESS: A study of very early mobilisation in Critical Care.

Invitation: This hospital is taking part in a national research study to investigate whether starting rehabilitation in the Intensive Care Unit, as soon as possible, will improve patients' long-term physical ability and quality of life.

When patients are sedated in the Intensive Care unit, muscle wasting and weakness can occur very quickly and this can take a long time to recover from. Because we feel that it may be important to deliver rehabilitation physiotherapy as early as possible, we wish for your relative/friend to participate in the trial.

Because your relative/friend is unable to decide for himself/herself whether to participate in this research, we'd like to ask your opinion as to whether or not they would want to be involved. Please consider what you know about their wishes and feelings and what you think may be best for them.

If we have been unable to contact you, your relative/friend may have been enrolled as a participant in this research project with the approval of their treating doctor and the Hampshire Research Ethics Committee (IRAS number: 250165). If this is the case, then we seek to confirm that you are in agreement.

Knowing what is involved will help you decide if you want your relative/friend to continue to take part in the research, so this information sheet explains the tests and treatments involved.

- Part 1 tells you about why we are doing this study and what will happen to your relative if they take part.
- Part 2 gives you more detailed information about how we will run the study.

If you decide your relative/friend would have no objection to taking part, we will ask you to read and sign a form that records your permission, called the consultee declaration. We'll then give you a copy to keep. We will keep you fully informed during the study so you can let us know if you have any concerns or you think your relative/friend should be withdrawn. Taking part in this research is entirely voluntary. If you decide not to continue, they will still be offered the best possible standard of care.

Please ask us if there is anything that is not clear or if you would like more information. Take time to decide whether or not you wish your relative/friend to take part.

For peer review only

PART ONE: Why are we doing this study and what will happen to my friend / relative?

What is the purpose of this study?

We know that when patients are very unwell and need sedation in the Intensive Care Unit, they can lose muscle strength and size very quickly. It is normal to offer rehabilitation, but this often starts after the patient has woken up. By this time the muscles have already been affected. Previous studies have shown that this muscle weakness may take many months to recover from and may affect a patient's quality of life after leaving hospital.

In Southampton Hospital, researchers and physiotherapists started performing rehabilitation exercises much earlier than usual, even while the patient was sedated. They showed that this method reduced the patient's time on the ventilator and reduced the amount of time that they needed to be in Intensive Care.

We are now trying to discover whether this method will work in a number of different hospitals in the UK. We will also do some tests to see whether the patients who have this type of rehabilitation are stronger and able to engage in physical activity more easily, when they leave hospital and 3 months later.

Why has my relative been chosen?

Your relative has been enrolled in this study because during their admission to the Intensive Care Unit he/she needed a ventilator (a machine to help them breathe) and sedation to help keep them calm and comfortable. The treating doctor and physiotherapist thought that either very early rehabilitation or standard rehabilitation would be equally suitable. We may have made a start already, because we are testing such very early rehabilitation, but we would like to ask for your permission to continue.

Does my relative/ friend have to take part?

No. It is up to you to decide whether or not you would like him/her to continue to take part. If you decide they can, you will be given this information sheet to keep and be asked to sign a permission form. You are still free to withdraw your relative at any time without giving a reason. The decision to withdraw at any time, or a decision not to take part, will not affect the standard of care your relative receives.

At an appropriate time, when we hope your relative has recovered sufficiently, we will ask their permission to use the data we have collected. If they do not agree we will not collect any new data and ask if we may use the data already collected.

What will happen to my relative if they take part?

Participation in the study will begin in the Intensive Care Unit. The final tests will take place 3 months after they leave hospital.

In the Intensive Care Unit: Your friend/ relative's treating doctor has assessed them to be eligible to take part in this study: EMPRESS. They were randomly allocated (like the flip of a coin) to receive either of the following:

For peer review only - http://bmjopen.bmj.com/site/about/guidelines.xhtml

- **Standard physiotherapy:** All patients on the trial will receive their normal physiotherapy. This will normally include activities to assist in keeping their airway clear and activities to maintain limb flexibility. These will not be affected by being on the trial.

OR

- **Standard physiotherapy, as above, plus an extra 2 sessions of 30 minutes of rehabilitation from Monday to Friday.** For patients receiving extra, early rehabilitation, in addition to their normal physiotherapy, they will start using a cycle machine that is designed to work, in the bed, even with sedated patients. As your friend/ relative wakes up they will start to pedal for themselves, do some more bed-based exercises and finally get out of bed and start moving. All of these sessions will be run by a well-trained physiotherapist and the bedside nurses. We have already tested this method in University Hospital Southampton and it has reduced the length of time on the ventilator and ICU stay. During these sessions, they will be very carefully monitored for their own safety and the safety of their lines, tubes and catheters.

  These exercises will continue for a maximum of 28 days or less if they leave the Intensive care unit before then.

BOTH GROUPS

- **Additional assessments:** So that we can test whether our new method works, patients on the trial will undertake some extra assessments. These include a simple test of grip strength by using a hand held pressure monitor; a test of arm and leg strength, ability to stand and step and mobility and walking tests. There will also be quality of life and health questionnaires.

There was a 50/50 chance of being allocated to either group. Neither you nor their doctor can decide which. No samples of blood are required for this research study.

In the hospital ward: When your friend/ relative has been discharged to a normal hospital ward, they won't receive any extra physiotherapy. Just before they go home, they will be tested again for muscle strength and mobility, including how far they can walk in 6 minutes. These tests will be supervised by a trained and experienced physiotherapist

Following discharge from hospital: Regardless of which group your friend/ relative was allocated to, after going home, they should follow the advice given to them by their doctors and physiotherapists. We have designed our study so that this will not affect our results.

They will be contacted by one of the critical care research team 3 months after they have been discharged home. We will arrange to see them for approximately one hour. During this visit we will test their walking speed, strength and agility. We will also ask for some questionnaires to be completed, which will assess how they feel about their quality of life and recovery.

The researchers would also like to have access to your relative or friend's medical record to obtain information relevant to the study. This information would be anonymised and kept confidential.

For peer review only - http://bmjopen.bmj.com/site/about/guidelines.xhtml

If you have any questions regarding the trial procedures, please don't hesitate to ask the intensive care or research doctors, physiotherapists and nurses.

What do I have to do?

It is important to tell the doctor and the research staff about any treatments or medications you know your relative/friend may have been taking, including over-the-counter medications, vitamins or herbal remedies, acupuncture or other alternative treatments. We would also like to know about any medical conditions which may affect the exercise.

Please just let us know if your relative/friend is involved in any other studies at this time.

What are the alternatives to participation?

Participation in this research is not the only option. You may decide for your relative to receive only standard care physiotherapy. That is absolutely fine. Please feel free to discuss these options with your relative's doctor before deciding whether or not to continue to take part in this research project.

What are the possible disadvantages of taking part?

Early mobility within ICU is safe. Potential risks may include, but not be limited to blood pressure or heart rate problems, breathing problems, problems with the tubes, lines and catheters.

In a review of physiotherapy within Intensive Care Units, involving over 1100 patients and 5267 episodes of physiotherapy in similar patients, there were 34 potential safety events (equal to 6 events in 1000 episodes of physiotherapy). Most of these were related to changes in heart rate or blood pressure and settles quickly on stopping the physiotherapy.

In Southampton, over a 4 year period, we have treated over 500 patients in this way and had 2 events needing attention but neither resulted in harm to the patient..

The doctors, physiotherapists and nurses who will be caring for your relative or friend while in the ICU, are trained to recognise the effects on the body associated with physical rehabilitation and will treat accordingly. Your friend/ relative will be continually monitored and assessed. Their safety will always be our number one priority.

There may be side effects that the researchers do not expect or do not know about and that may be serious. Please tell the doctor immediately if you are worried about any new or unusual symptoms that your relative/friend gets.

What are the possible benefits of taking part?

We cannot guarantee or promise that your relative will receive any benefits from this research. This study aims to further medical knowledge and may improve future treatment of patients who need to be on a ventilator, however it may not directly benefit your relative/friend.

For peer review only - http://bmjopen.bmj.com/site/about/guidelines.xhtml

For how long will my relative/ friend be in the research study?

The final research assessment will take place 3 months after discharge from hospital. Once that is done, your friend/ relative's participation in the study will end.

What happens if there is a problem?

We will keep you and your friend/ relative, fully informed of any problems which may be related to the study.

Will taking part in the study be kept confidential?

Yes. All of the information about participation and the data collected will be kept confidential.

Information held by the NHS and records maintained by the NHS Information Centre and the NHS Central Register may be used to help contact your friend/ relative and provide information about their health status. This information may be obtained and stored by the study research team to enable long term follow-up.

University Hospital Southampton is the sponsor for this study based in the United Kingdom. We will be using information from their medical records in order to undertake this study and will act as the data controller for this study. This means that we are responsible for looking after their information and using it properly. University Hospital Southampton will keep information about them for 10 years after the study has finished.

Your friend/ relative's rights to access, change or move your information are limited, as we need to manage the information in specific ways in order for the research to be reliable and accurate. If they withdraw from the study, we will keep the information that we have already obtained. To safeguard their rights, we will use the minimum personally-identifiable information possible.

You can find out more about how we use information at https://www.hra.nhs.uk/information-about-patients/

[Local NHS site name] will collect information from their medical records for this research study in accordance with our instructions.

(Local NHS site name) will keep name, NHS number and contact details confidential and will not pass this information to University Hospital Southampton. [Local NHS site name] will use this information as needed, to contact your relative/ friend about the research study, and make sure that relevant information about the study is recorded for their care, and to oversee the quality of the study. Certain individuals from University Hospital Southampton and regulatory organisations may look at medical and research records to check the accuracy of the research study. University Hospital Southampton will only receive information without any identifying information. The people who analyse the information will not be able to identify patients and will not be able to find out their name, NHS number or contact details.

[Local NHS site name] will keep identifiable information, including the consent form from this study, for 10 years after the study has finished.

For peer review only - http://bmjopen.bmj.com/site/about/guidelines.xhtml

Contact Details:

Local PI details

Address

Dr local PI: 02381 XXXXXX

Research Nurse: 02381 XXXXXX

ICU: 02381 XXXXXX

PART 2: How we will run this study.

What if relevant new information becomes available?

During the research project, new information about the risks and benefits of the study may become known to the researchers. If this occurs, you will be told about this new information and the doctor will discuss whether this new information affects your relative.

If any information becomes available which could affect participation in the study the research doctor will tell you about it and discuss whether you want your relative to continue in the study. If you decide your relative should not continue in the study, the research doctor will make arrangements for your relative's care to continue as normal. If you decide to allow your relative to continue in the study you will be asked to sign an updated agreement form.

Also, on receiving new information the research doctor might consider it to be in your relative's best interests to withdraw them from the study. He/she will explain the reasons and arrange for their care to continue.

If the study is stopped for any other reason, you will be told why and your relative's continuing care will be arranged.

What will happen if I don't want my relative to carry on with the study?

You can withdraw your relative from the study at any time without giving an explanation and be assured that it will not impact on any part of your relative's further treatment.

If you decide to withdraw your relative from the study, the researchers would like to keep your relative's health information that has been collected. This is to help them make sure that the results of the research can be measured properly. If you do not want them to do this, you can tell them when you withdraw your relative from the research project.

What if there is a problem?

If you have any concerns regarding the study, please ask to speak to the ICU doctor in charge of your friend/ relative's care or ask to speak to (name of local PI), the consultant who is in charge of the study.

For peer review only - http://bmjopen.bmj.com/site/about/guidelines.xhtml

Complaints:

If you have a concern about any aspect of this study, you should ask to speak with the researchers or the Intensive Care doctors and nurses, who will do their best to answer your questions. If you remain unhappy and wish to complain formally, you can do this through the NHS Complaints Procedure. (Please localise with your hospital PALS contact details)

Harm:

In the event that something does go wrong and your relative is harmed during the research study there are no special compensation arrangements. If your relative is harmed and this is due to someone's negligence then your relative may have grounds for a legal action for compensation against University Hospital Southampton, but they may have to pay the legal costs. The normal National Health Service complaints mechanisms will still be available to them.

Involvement of the General Practitioner/Family doctor (GP)

If you are agreeable we would like to inform your friend/ relative's GP of their participation in the study. If you do not wish for their GP to be informed, please let us know and indicate on the consent form that you do not wish for their GP to be informed.

Will allowing my relative to take part in this study be kept confidential?

If your relative joins the study, some parts of their medical records and the data collected for the study will be looked at by authorised persons from University Hospital Southampton and University of Southampton who are sponsoring and organising the research. They may also be looked at by authorised people to check that the study is being carried out correctly. All will have a strict duty of confidentiality to your relative as a research participant and we will do our best to meet this duty.

All information that is collected about your relative during the course of the research will be kept strictly confidential. Any information about your relative that leaves the hospital will have their name and address removed so that they cannot be recognised from it.

Anonymised data collected during the study may be sent to associated researchers in other countries, where the laws don't protect your privacy to the same extent as the law in the UK but the study team will take all reasonable steps to protect your privacy.

Your relative has the right to check the accuracy of data held about them and correct any errors.

What will happen to the results of the research study?

They will be published in a medical journal, presented at conferences and lay press where possible.

For peer review only - http://bmjopen.bmj.com/site/about/guidelines.xhtml

Who is organising and funding the research?

Dr Rebecca Cusack from University Hospital Southampton is the lead researcher, who is organising the research.

The research is funded by the NHS through the National Institute for Health Research, Research for Patient Benefit scheme.

Who has reviewed the study?

Hampshire Research Ethics Committee (IRAS number: 250165) have reviewed this study and given their approval.

Thank you very much for taking the time to read this information sheet at this very stressful time.

If you have any further questions please ask the doctors in Intensive Care, Dr (local PI) or one of the research team.

If you agree to your relative participating in this study please keep this information sheet and you will be given a copy of the agreement form that you will be asked to sign.

For peer review only - http://bmjopen.bmj.com/site/about/guidelines.xhtml

FORM TO BE ON SITE SPECIFIC HEADED PAPER

University Hospital Southampton NHS
NHS Foundation Trust

Critical Care, Anaesthesia & Peri-operative Medicine Dept,
Research office CE 93. MP 24,
University Hospital Southampton,
Tremona Road,
Southampton
SO16 6YD

Tel: 023 8120 5308
Fax: 023 8120 5378

For peer review only

Consultee declaration form for patients participating in EMPRESS. A feasibility study of early mobilisation programmes in Critical Care.

Name of Researcher: ________________________ Please initial box

1. I ____________________ [name of consultee] have been consulted about ____________________ [name of potential participant]'s participation in this research project. I have read and understood the Consultee Information Sheet (version ___; Dated ____________). I have had the opportunity to ask questions about the study and understand what is involved. ☐

2. In my opinion he/she would have no objection to taking part in the above study. ☐

3. I understand that I can request he/she is withdrawn from the study at any time, without giving any reason and without his/her care or legal rights being affected. ☐

4. I understand that relevant sections of his/her medical notes and data collected during the study, may be looked at by individuals from the research team, from regulatory authorities or from the NHS Trust, where it is relevant to their taking part in this research. I give permission for these individuals to have access to their records. ☐

5. I understand that information held by the NHS and records maintained by the NHS Information Centre and the NHS Central Register may be used to help contact me or my friend / relative and provide information about their health status. I give permission for this information to be obtained and stored by the study research team to enable long term follow-up. ☐

6. I agree to their GP being informed of their participation in the study. ☐

When completed: 1 for participant; 1 (original) for researcher site file; 1 to be kept in medical notes.
Version 2: 29th January 2019 IRAS ID:250165

Please confirm either:

I confirm that I will act as the personal consultee for: ______________________

Relationship to participant: ______________________

Name of consultee:	Signature:	Date:
______________________	______________________	______________
Person undertaking consultation (researcher):	Signature:	Date:
______________________	______________________	______________

***Original** Informed Consent form to be filed in the Investigator Site File.*

1 copy to be given to the patient

1 copy to be filed in the patients' hospital notes.

For peer review only - http://bmjopen.bmj.com/site/about/guidelines.xhtml
For peer review only

SPIRIT

STANDARD PROTOCOL ITEMS: RECOMMENDATIONS FOR INTERVENTIONAL TRIALS

SPIRIT 2013 Checklist: Recommended items to address in a clinical trial protocol and related documents*

Section/item	Item No	Description
**Administrative information**		
Title	1	Descriptive title identifying the study design, population, interventions, and, if applicable, trial acronym Pg1 lines 1-2
Trial registration	2	Trial identifier and registry name. If not yet registered, name of intended registry Pg2 line 28
Protocol version	3	Date and version identifier Pg 14 line 410
Funding	4	Sources and types of financial, material, and other support Pg 14 lines 403-4
Roles and responsibilities	5a	Names, affiliations, and roles of protocol contributors Title page
	5b	Name and contact information for the trial sponsor Pg2 line 29
	5c	Role of study sponsor and funders, if any, in study design; collection, management, analysis, and interpretation of data; writing of the report; and the decision to submit the report for publication, including whether they will have ultimate authority over any of these activities N/A
	5d	Composition, roles, and responsibilities of the coordinating centre, steering committee, endpoint adjudication committee, data management team, and other individuals or groups overseeing the trial, if applicable (see Item 21a for data monitoring committee) Trial management group/ Data safety group/PPI group -- Pg12 Lines 315 to Pg 13 line 334
**Introduction**		
Background and rationale	6a	Description of research question and justification for undertaking the trial, including summary of relevant studies (published and unpublished) examining benefits and harms for each intervention See introduction Pg3 line 44-Pg line 80
	6b	Explanation for choice of comparators See introduction Pg3 line 44-Pg line 80
Objectives	7	Specific objectives or hypotheses Pg 3 lines 82-88

For peer review only - http://bmjopen.bmj.com/site/about/guidelines.xhtml

Trial design	8	Description of trial design including type of trial (eg, parallel group, crossover, factorial, single group), allocation ratio, and framework (eg, superiority, equivalence, noninferiority, exploratory) Study design Pg 4 lines 91-97

Methods: Participants, interventions, and outcomes

Study setting	9	Description of study settings (eg, community clinic, academic hospital) and list of countries where data will be collected. Reference to where list of study sites can be obtained Pg 4 Lines 93-95
Eligibility criteria	10	Inclusion and exclusion criteria for participants. If applicable, eligibility criteria for study centres and individuals who will perform the interventions (eg, surgeons, psychotherapists) Pg4 Lines 99 -108
Interventions	11a	Interventions for each group with sufficient detail to allow replication, including how and when they will be administered Pg 5- 8 lines 137 -198
	11b	Criteria for discontinuing or modifying allocated interventions for a given trial participant (eg, drug dose change in response to harms, participant request, or improving/worsening disease) Table 1
	11c	Strategies to improve adherence to intervention protocols, and any procedures for monitoring adherence (eg, drug tablet return, laboratory tests)N/A
	11d	Relevant concomitant care and interventions that are permitted or prohibited during the trial N/A
Outcomes	12	Primary, secondary, and other outcomes, including the specific measurement variable (eg, systolic blood pressure), analysis metric (eg, change from baseline, final value, time to event), method of aggregation (eg, median, proportion), and time point for each outcome. Explanation of the clinical relevance of chosen efficacy and harm outcomes is strongly recommended Primary outcomes Pg8 lines 200-208; Secondary outcomes lines 210-244
Participant timeline	13	Time schedule of enrolment, interventions (including any run-ins and washouts), assessments, and visits for participants. A schematic diagram is highly recommended (see Figure) Table 2
Sample size	14	Estimated number of participants needed to achieve study objectives and how it was determined, including clinical and statistical assumptions supporting any sample size calculations Pg 10 line 284-300
Recruitment	15	Strategies for achieving adequate participant enrolment to reach target sample sizePg5 Lines 110-127

Methods: Assignment of interventions (for controlled trials)

For peer review only - http://bmjopen.bmj.com/site/about/guidelines.xhtml

Allocation:

Sequence generation	16a	Method of generating the allocation sequence (eg, computer-generated random numbers), and list of any factors for stratification. To reduce predictability of a random sequence, details of any planned restriction (eg, blocking) should be provided in a separate document that is unavailable to those who enrol participants or assign interventions Pg10 Line 126-7
Allocation concealment mechanism	16b	Mechanism of implementing the allocation sequence (eg, central telephone; sequentially numbered, opaque, sealed envelopes), describing any steps to conceal the sequence until interventions are assigned Pg10 line 126-7
Implementation	16c	Who will generate the allocation sequence, who will enrol participants, and who will assign participants to interventions Pg10 line 126-7
Blinding (masking)	17a	Who will be blinded after assignment to interventions (eg, trial participants, care providers, outcome assessors, data analysts), and how Pg14 line 390
	17b	If blinded, circumstances under which unblinding is permissible, and procedure for revealing a participant's allocated intervention during the trial N/A

Methods: Data collection, management, and analysis

Data collection methods	18a	Plans for assessment and collection of outcome, baseline, and other trial data, including any related processes to promote data quality (eg, duplicate measurements, training of assessors) and a description of study instruments (eg, questionnaires, laboratory tests) along with their reliability and validity, if known. Reference to where data collection forms can be found, if not in the protocol Lines 278 - 286
	18b	Plans to promote participant retention and complete follow-up, including list of any outcome data to be collected for participants who discontinue or deviate from intervention protocols Lines 306-7
Data management	19	Plans for data entry, coding, security, and storage, including any related processes to promote data quality (eg, double data entry; range checks for data values). Reference to where details of data management procedures can be found, if not in the protocol Lines 279-86
Statistical methods	20a	Statistical methods for analysing primary and secondary outcomes. Reference to where other details of the statistical analysis plan can be found, if not in the protocol Lines 305- 317
	20b	Methods for any additional analyses (eg, subgroup and adjusted analyses) N/A

For peer review only - http://bmjopen.bmj.com/site/about/guidelines.xhtml

	20c	Definition of analysis population relating to protocol non-adherence (eg, as randomised analysis), and any statistical methods to handle missing data (eg, multiple imputation) Line 84
**Methods: Monitoring**		
Data monitoring	21a	Composition of data monitoring committee (DMC); summary of its role and reporting structure; statement of whether it is independent from the sponsor and competing interests; and reference to where further details about its charter can be found, if not in the protocol. Alternatively, an explanation of why a DMC is not needed Lines 332-333
	21b	Description of any interim analyses and stopping guidelines, including who will have access to these interim results and make the final decision to terminate the trial N/A
Harms	22	Plans for collecting, assessing, reporting, and managing solicited and spontaneously reported adverse events and other unintended effects of trial interventions or trial conduct Lines 351-361
Auditing	23	Frequency and procedures for auditing trial conduct, if any, and whether the process will be independent from investigators and the sponsor N/A
**Ethics and dissemination**		
Research ethics approval	24	Plans for seeking research ethics committee/institutional review board (REC/IRB) approval Line 24-27
Protocol amendments	25	Plans for communicating important protocol modifications (eg, changes to eligibility criteria, outcomes, analyses) to relevant parties (eg, investigators, REC/IRBs, trial participants, trial registries, journals, regulators) .Line 25
Consent or assent	26a	Who will obtain informed consent or assent from potential trial participants or authorised surrogates, and how (see Item 32)Lines 109-122
	26b	Additional consent provisions for collection and use of participant data and biological specimens in ancillary studies, if applicable N/A
Confidentiality	27	How personal information about potential and enrolled participants will be collected, shared, and maintained in order to protect confidentiality before, during, and after the trial Line 279
Declaration of interests	28	Financial and other competing interests for principal investigators for the overall trial and each study site Line 413
Access to data	29	Statement of who will have access to the final trial dataset, and disclosure of contractual agreements that limit such access for investigators Line 326-7 and 332-333

review only

For peer review only - http://bmjopen.bmj.com/site/about/guidelines.xhtml

Ancillary and post-trial care	30	Provisions, if any, for ancillary and post-trial care, and for compensation to those who suffer harm from trial participation Lines 360-361
Dissemination policy	31a	Plans for investigators and sponsor to communicate trial results to participants, healthcare professionals, the public, and other relevant groups (eg, via publication, reporting in results databases, or other data sharing arrangements), including any publication restrictions Lines 343-345
	31b	Authorship eligibility guidelines and any intended use of professional writersLines 402-406
	31c	Plans, if any, for granting public access to the full protocol, participant-level dataset, and statistical code Lines 348-
**Appendices**		
Informed consent materials	32	Model consent form and other related documentation given to participants and authorised surrogates Supplied
Biological specimens	33	Plans for collection, laboratory evaluation, and storage of biological specimens for genetic or molecular analysis in the current trial and for future use in ancillary studies, if applicable N/A

*It is strongly recommended that this checklist be read in conjunction with the SPIRIT 2013 Explanation & Elaboration for important clarification on the items. Amendments to the protocol should be tracked and dated. The SPIRIT checklist is copyrighted by the SPIRIT Group under the Creative Commons "Attribution-NonCommercial-NoDerivs 3.0 Unported" license.

For peer review only
